# Evaluating the cost of malaria elimination by *Anopheles gambiae* precision guided SIT in the Upper River region, The Gambia

**DOI:** 10.1371/journal.pgph.0004903

**Published:** 2025-07-18

**Authors:** William A. C. Gendron, Robyn Raban, Agastya Mondal, Héctor M. Sánchez C, Andrea L. Smidler, David Zilberman, Patrick G. C. Ilboudo, Umberto D’Alessandro, John M. Marshall, Omar S. Akbari

**Affiliations:** 1 School of Biological Sciences, Department of Cell and Developmental Biology, University of California, San Diego, La Jolla, California, United States of America; 2 Divisions of Epidemiology & Biostatistics, School of Public Health, University of California, Berkeley, California, United States of America; 3 Department of Agricultural and Resource Economics, University of California, Berkeley, California, United States of America; 4 Chronic Diseases Management Unit, African Population and Health Research Center, Nairobi, Kenya; 5 Medical Research Council Unit The Gambia at the London School of Hygiene and Tropical Medicine, Fajara, The Gambia; 6 Innovative Genomics Institute, University of California, Berkeley, California, United States of America; Federal University Birnin Kebbi, NIGERIA

## Abstract

Mosquito control has successfully reduced the burden of malaria globally, but current vector control technologies cannot achieve malaria elimination. Precision guided sterile insect technique (pgSIT) is one of the most promising interventions being developed for malaria elimination. Mass release of genetically sterile males can act as a chemical-free species-specific insecticide. Before translating pgSIT from the bench to the field, however, it is essential to understand the potential costs and capabilities of this technology in a malaria-endemic region to determine if further investment into research and development of this technology is worthwhile. Therefore, we estimated the health outcomes and costs of a pgSIT program working jointly with current interventions to control the *Anopheles gambiae* malaria vector in the Upper River region of The Gambia. The pgSIT intervention in this region is predicted to prevent approximately 230 deaths and about 48,000 sick days per year. We have provided a range of costs that include risks associated with research and development, the facility, mass rearing efficiency, and distribution. This intervention should save disability-adjusted life years (DALY) at 11–94 USD per year and will prevent cases at 10–86 USD per infection. These estimates predict that pgSIT will cost 0.36-3.03 USD per person in the treated region annually. The cost per DALY shows life-saving at a cost comparable to current interventions in the Upper River region.

## Significance statement

This study investigates pgSIT as a suppression technology for the malaria mosquito, *Anopheles gambiae,* in the Upper River region of The Gambia. We evaluate the pgSIT mosquito release requirements, develop a modified protocol from existing methods, estimate costs associated with the scale and distribution of this intervention, and calculate the expected lives saved and cases prevented. This evaluation shows that compared to current public health interventions, pgSIT can prevent more malaria cases and save lives at a competitive cost per DALY saved, cost per case prevented, and cost per person covered. The trials discussed throughout can act as time horizons to reevaluate the technology as more primary data is generated.

## Introduction

Malaria kills approximately 700,000 people a year, with African children under five years old accounting for most malaria-related deaths [1]. Progress has been made in disease prevention and treatment, which has reduced the burden of malaria significantly, but further efforts are required to eliminate this disease. Mosquito control is the cornerstone of mosquito-borne disease prevention. Long-lasting insecticidal nets (LLINs) and indoor residual spraying (IRS) are the primary tools currently used to reduce human-mosquito contact [2–4]. However, pervasive insecticide resistance in malaria vector populations has limited the capabilities of insecticides [5]. Malaria vaccines are now available, but despite the considerable effort in this space, they still require multiple boosters to achieve 66% efficacy [6].

Genetic strategies are the next generation of tools in development to support malaria elimination. Self-limiting technologies, such as the Release of Insects carrying a Lethal Dominant gene (RIDL), have been demonstrated to reduce populations of the dengue vector, *Aedes aegypti [*7*]*, but have been challenging to develop for malaria vector control. Gene drive technology, on the other hand, has been built in multiple malaria vector species to suppress [8–11] and modify vector populations so they cannot transmit malaria [12–16]. The current state of gene drive technology, however, is technically complicated with issues of drive resistance and uncertainties about their behavior in large-genetically diverse populations that need to be addressed before they transition to the field [17]. In many cases, gene drives are highly controversial, as they are engineered to persist and spread beyond their release site, so it is unknown whether these technologies will receive regulatory and community approval for wide-scale use [18].

Precision-guided sterile insect technique (pgSIT) combines the benefits of sterile insect technique (SIT) with the precision and adaptability of CRISPR-based genetic engineering. Despite success in some species [19–21], the radiation used to sterilize males in traditional SIT causes imprecise mutations, which results in fitness costs that impact the cost and scalability of SIT technologies [22]. PgSIT, on the other hand, uses genetic engineering tools to generate fit and competitive sterile males by precisely disrupting female viability and male fertility genes [23–28]. The homozygous Cas9 and gRNA lines are maintained separately and then crossed, resulting in the death and sterilization of female and male offspring, respectively. To enable scaling these crosses, pgSIT can be linked with a fluorescent sex-sorting technology like SEPARATOR, which has been used to accurately and efficiently sex sort various insects, including multiple species of mosquitoes [29–32]. This effect occurs in early embryogenesis, so pgSIT can be released at any life stage, which reduces the handling and transport of fragile adult stages. Eggs, for example, can be transported to the release site and hatched in rearing pools, and only sterile males will emerge. These distribution procedures require limited resources and training, enabling pgSIT sterile male releases in remote locations worldwide.

With any new malaria intervention, it is essential to determine feasibility by estimating the costs, lives saved, and cases prevented in a malaria-endemic region. We are conducting an initial cost assessment of pgSIT to determine the economic feasibility of scaling this technology to generate the data for field trials and wide-scale applications. We focused our analysis on pgSIT control of the *Anopheles gambiae* malaria vector in the Upper River region (URR), The Gambia, since *A. gambiae* and its sibling species are the primary malaria vectors in the region. This region has the highest per capita malaria rates in The Gambia, reliable cross-sectional survey data on malaria incidence and prevalence [33], comprehensive data on malaria-associated healthcare and prevention costs, and human demographics. Additionally, pgSIT is a novel intervention and will be treated as an additive measure to the standard of care of LLINs, IRS, and antimalarial drugs. While pgSIT may eventually replace these conventional methods, removing these established methods is unlikely until pgSIT has had an extensive track record.

A cost analysis of pgSIT technology is predicated on understanding how pgSIT will be implemented in the URR and its achievable vector and disease suppression. Mathematical modeling using laboratory efficacy and fitness data can predict outcomes of pgSIT sterile male release scenarios and their potential to suppress populations of the primary malaria vector in the region, *A. gambiae*. Models can also be used to predict impacts on malaria transmission.

The cost analysis includes estimated costs to continue the development of the pgSIT technology, and the equipment, construction, personnel, and other costs needed to establish a facility to produce and distribute pgSIT sterile males at the rate predicted to suppress the *A. gambiae* population in the URR completely. Herein, a range of cost estimates that capture the current technological and production uncertainties of pgSIT are used to quantify the economic benefits of pgSIT release programs. Additionally, each of these development trials sets a time horizon to evaluate the capability of the technology and gather data that will inform the efficiencies of this intervention [34]. These trials will help minimize investment risk by allowing for re-evaluation of the cost effectiveness of this technology at each step. These trials will also provide general information regarding *Anopheles gambiae* mass rearing, which is applicable to any other genetic control method for these mosquitoes.

## Methods

### Mathematical modeling

**Fig 1:**
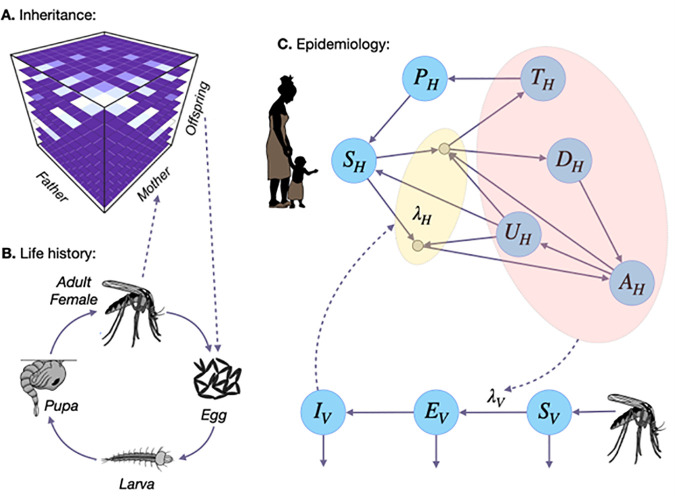
Modules of the MGDrivE 3 modeling framework. **(A)** Genetic inheritance is embodied by a three-dimensional tensor referred to as an “inheritance cube.” Maternal and paternal genotypes are depicted on the x and y-axes and offspring genotypes on the z-axis. **(B)** Mosquito life history is modeled according to an egg-larva-pupa-adult (female and male) life cycle in which density dependence occurs at the larval stage, and life cycle parameters may vary as a function of environmental variables over time. Genotypes are tracked across all life stages, and females obtain a composite genotype upon mating - their own and that of the male they mate with. Egg genotypes are determined by the inheritance cube. **(C)** The epidemiology module describes the reciprocal transmission of malaria between mosquitoes and humans. Adult female mosquitoes progress from susceptible (*S*_*V*_) to exposed/latently infected (*E*_*V*_) to infectious for malaria (*I*_*V*_). Transmission in the human population is modeled according to the Imperial College London malaria model, in which humans progress from susceptible (*S*_*H*_) to either symptomatic or asymptomatic infection. Humans who develop a symptomatic infection and are either treated (*T*_*H*_) or diseased and untreated (*D*_*H*_). Treated humans advance to a prophylactic protection state (*P*_*H*_) and eventually become susceptible again. Untreated symptomatic humans develop successively lower-density infections, from symptomatic to asymptomatic but detectable by rapid diagnostic test (RDT) (*A*_*H*_) to asymptomatic and undetectable by RDT (*U*_*H*_). Asymptomatic humans can also be super-infected. Communication between the two frameworks is facilitated by the forces of infection in humans (*λ*_*H*_) and vectors (*λ*_*V*_).

We used the MGDrivE 3 framework [35] to simulate releases of pgSIT *A. gambiae* mosquitoes to suppress malaria in the URR of The Gambia (Fig 1). MGDrivE 3 is a modular framework for simulating releases of genetic control systems in spatially-structured mosquito populations which includes modules for: i) inheritance (i.e., the dynamics of the pgSIT system) (Fig 1A), ii) life history (i.e., the development of mosquitoes from egg to larva to pupa to adult, including species-specific bionomic parameters) (Fig 1B), and iii) epidemiology (i.e., the reciprocal transmission of malaria parasites between mosquitoes and humans) (Fig 1C). We simulated the inheritance pattern of the pgSIT system within the inheritance module of MGDrivE [36], the life history of *A. gambiae* using standard bionomic parameters (S1 Table) and seasonality in larval carrying capacity [37] driven by rainfall data for The Gambia, and calibrated malaria transmission to incidence data from the URR of The Gambia [33]. While multiple release parameters will lead to vector elimination, the release conditions were selected to minimize cost by reducing the number of mosquitoes released weekly. Full details of the MGDrivE 3 modeling framework are described in Mondal *et al.* [35].

### PgSIT inheritance dynamics model

The inheritance pattern of the pgSIT system was modeled within the inheritance module of MGDrivE [36] (Fig 1A). Based on laboratory data in *A. gambiae*, we assumed the pgSIT system would induce complete male sterility and female inviability, with offspring of females mated to sterile males failing to develop as these eggs are not fertilized [23]. We assumed that pgSIT eggs would be introduced into the environment in cups with sufficient water volume and larval resources so that larval mortality would be density-independent. Survival of eggs released in cups was therefore determined by expected juvenile life stage durations and their daily mortality rates (S1 Table), leading to a viable emergence rate of 26% for male eggs. The pgSIT inheritance cube also allows impacts of the construct on adult lifespan and male mating competitiveness to be incorporated into the model. Based on laboratory data [23], we assumed a 50% reduction in pgSIT male mating competitiveness. To be conservative, we also assumed a 25% reduction in pgSIT male lifespan compared to wild-type males, as fitness costs sometimes emerge in the field [7], although no reductions in lifespan have been observed for pgSIT in the laboratory [23,27,38].

#### A. gambiae life history model.

The MGDrivE 3 framework [35] models the development of mosquitoes from egg to larva to pupa to adult with overlapping generations, larval mortality increasing with larval density [37], and a mating structure in which females retain the genetic material of the adult male with whom they mate for the duration of their adult lifespan (Fig 1B). Species-specific bionomic parameters for *A. gambiae* are listed in the S1 Table. The life history module of MGDrivE 3 permits life history parameters to change with time. Given the dependence of *A. gambiae* populations on recent rainfall to provide egg-laying and juvenile development sites, we utilized rainfall data from the URR sourced from Climate Hazards Group InfraRed Precipitation with Station data (CHIRPS, https://www.chc.ucsb.edu/data/chirps) to calibrate a seasonal time-series for larval carrying capacity, which is a major driver for their population dynamics. To smooth the seasonal profile of the raw rainfall data, we leveraged a Fourier analysis-based approach that involves fitting a mixture of sinusoids to the raw data (https://github.com/mrc-ide/umbrella) to depict general seasonal trends without a level of daily detail that is not replicated from year to year. The fitted seasonality profile for the URR is shown in S1 Fig.

### Malaria transmission model

To model epidemiological outcomes associated with pgSIT mosquito releases, we linked the MGDrivE 3 framework [35] to the Imperial College London (ICL) malaria model [39,40]. Linking the two models was achieved by allowing forces of infection (i.e., the probability of infection from mosquito-to-human and human-to-mosquito per individual per unit time) to be exchanged between the two models. We chose the ICL malaria model as it provides a validated and parsimonious framework to describe transmission in human populations, including important features such as acquired and maternal immunity, symptomatic and asymptomatic infection, superinfection, age structure, biting heterogeneity, antimalarial drug therapy, and prophylaxis. The model describes humans moving through various infection states (susceptible, treated and untreated symptomatic disease, asymptomatic patent and subpatent infection, and prophylactic protection) and includes various forms of immunity (maternal, acquired, and pre-erythrocytic), which reduce the probability of severe and clinical disease (Fig 1C). The model is age-structured, allowing outcomes to be estimated for chosen age groups. Full details of the ICL malaria model are described in Griffin *et al.* [40].

The ICL malaria model was calibrated using malaria prevalence data from a randomized-control trial of mass drug intervention in the URR [33]. The study found a baseline malaria prevalence in the URR of ~18% at the beginning of the rainy season, which we aligned with the simulation output. Furthermore, entomological data from the URR [41] suggested vector breeding sites in this region are abundant in the rainy season and minimal in the dry season, so for modeling purposes, we assumed that larval carrying capacity in the dry season was 10% that of the peak rainy season. Malaria model calibration occurred in the context of existing levels of coverage of artemisinin-based combination therapy drugs (ACTs), LLINs, and IRS. For the URR, data from the Malaria Atlas Project [42] was used to parameterize an LLIN coverage of 55%, an IRS coverage of 52%, and 50% of symptomatic malaria cases treated by antimalarial drugs. The remaining parameters of the malaria model were taken from the ICL implementation [39,40], including a mortality rate of severe malaria cases of 21.5%, based on hospital case data collected in Tanzania [39].

In sum, strengths of the modeling framework are that: i) inheritance dynamics of the pgSIT system and its impacts of mosquito bionomic parameters can be flexibly modeled within MGDrivE [35,36], ii) the model of mosquito life history is detailed and responsive to local rainfall data, and iii) the ICL malaria model incorporates many important features of malaria transmission, has been validated against datasets throughout sub-Saharan Africa [39,40], and is calibrated to local epidemiological data. Limitations of the framework are that: i) spatial structure has been ignored, ii) movement of humans and mosquitoes in and out of the URR has been ignored, and iii) the intervention model has been calibrated to laboratory data, but not to field data, which if collected, would allow for much more reliable predictions.

### Anopheles gambiae mass rearing facility cost assessment

Numerous options were considered for the facility design. The production facility and protocol were modified from the mass rearing protocol developed by the International Atomic Energy Agency (IAEA) [43] to enable the crossing of the two pgSIT strains and to accommodate delivery tools for pgSIT. To determine the required rearing numbers to suppress mosquitoes in the URR, IAEA, and laboratory estimates for fecundity, fertility, and survival were used to provide a production range, which informed the facility and equipment requirements. When available, we obtained local cost estimates, and when necessary, international prices were adjusted to account for currency conversion, importation, shipping, and other associated acquisition costs. The estimated costs to further develop pgSIT in field trials, facility start-up, and annual operations are integral to the cost-benefit analysis. The costs are divided into four sections to distinguish between cost calculations: research and development costs (Section 1.1 in S1 Text, S2–S6, S9, S10, S22 Tables), initial facility and equipment costs (Section 1.2 in S1 Text, S9–S12, S15, S16, S18–S20, S23, S24, S28, S29 Tables), annual production, distribution, and maintenance costs (Section 1.3 in S1 Text, S2, S11–S19, S24–S27, S30 Tables), and rearing costs at the release site (Section 1.4 in S1 Text, S20 Table). Research and development costs were estimated based on previous trial costs and similar research projects within The Gambia. There was limited data for several key costs, so in these instances, high and low estimates from available data were used to capture the cost-associated risk. These estimates were then used directly, or the average cost was applied (Table 2). To be more directly comparable to current or upcoming interventions, costs were divided into total costs, post research and development costs, and annual costs. Total costs include research and development and facility construction costs, each with a high, average, or low cost estimate. Post research and development costs are more comparable to current interventions and only include the facility construction and initial training. To capture the time preference, a discount rate of 3% was applied, as this is typical for medical interventions [44]. A discount rate was applied for 10 years to the initial investment, then averaged across 20 years of the annual facility production costs [45]. This cost assessment is described in depth in Section 1 in S1 Text and previously as a preprint but has been iterated on within the supplemental [46]. We have included all known costs, but there may be unforeseen costs that cannot be estimated. Costs are estimated in 2022 USD.

### Cost of intervention per person estimations

To estimate the per person cost of pgSIT, we divided the annual facility cost by the expected population of the URR in 2030 (the earliest expected year for large-scale release). The 2030 population size in the URR was estimated from the average population growth in the region over 10 years extrapolated to the population from the last census year (2013) until 2040 (S41 Table) [47]. If the population continues to grow as predicted, this is an underestimate of the cost per person. The mosquito population is not expected to change in a predictable way in relation to the increased human population, as the availability of aquatic larval sites is the primary limitation of the mosquito population.

### Utilizing cases prevented and lives saved to derive cost per life year estimations

The malaria transmission model previously estimated the reduction in malaria cases and deaths after pgSIT implementation (Table 1). This model includes pgSIT implementation with current interventions, so the calculated reduction in malaria cases is beyond the standard of care treatment. The deaths are matched to age categories, and the estimated life years saved are determined by subtracting the average age in each category from the life expectancy of The Gambia, which is 62.6 years (S31–S32 Tables). For the 60 years or older category, we assume one life year is saved per death prevented. The total life years saved and cases prevented annually can then be used to determine a cost per life year saved or per malaria case prevented. To compare pgSIT to current vector control interventions, we applied the highest costs in each category to estimate the costs per disability-adjusted life year (DALY) and case prevented. Each cost category estimates the intervention cost at a pivotal stage of development. The total facility cost takes the initial investment for research and development and facility construction and averages that cost over the first 20 years of the intervention. This 20 year average is then added to the annual production costs for each condition The costs are divided into the initial research and development investment, facility construction, and egg rearing at the release sites. This approach facilitates more accurate comparisons to other interventions that do not involve research and development. The total costs include research and development, facility construction, and annual costs. The initial costs of research and development and facility construction were then averaged across the first 10 years of production to get the cost per life year saved or case prevented (Table 2, Section 1 in S1 Text, S9, S10, S25, S26 and S27 Tables). The post-research and development estimate is the total cost, excluding the pgSIT research and development costs (S10 Table). The annual costs include only producing the sterile male eggs, maintaining the facility, and rearing sterile males in the field post-production (S30 Table). The last estimate is the annual production costs, which include sterile male production and facility maintenance costs and exclude rearing costs at the release site. These rearing costs at the release sites are variable and dependent on local resources and capabilities but may be negligible if water and larval food can be easily sourced at the release sites (S25–S27 Tables).

**Table 1:** Model-predicted annual malaria cases and deaths averted for 12 releases of 32 pgSIT eggs per wild adult in the URR of The Gambia.

Intervention Year	0-5 years		5-17 years		17-40 years		40-60 years		≥60 years		Total	
**Cases**	**Deaths**	**Cases**	**Deaths**	**Cases**	**Deaths**	**Cases**	**Deaths**	**Cases**	**Deaths**	**Cases**	**Deaths**
**0**	1	0	5	0	6	0	1	0	1	0	13	0
**1**	0	0	-3	0	-4	0	-1	0	0	0	-9	0
**2**	452	48	3,777	114	4,877	16	973	3	594	2	10,673	184
**3**	546	57	4,567	141	5,915	20	1,182	4	723	3	12,933	225
**4**	544	57	4,550	143	5,904	21	1,182	4	724	3	12,903	228
**5**	545	57	4,562	146	5,928	21	1,189	4	729	3	12,953	232

**Table 2:** Costs per disability adjusted life year saved and cost per case averted.

Costs	Initial Costs	Discount Rate Applied to Initial Costs (10 Years, 3%)	Annual Production Costs	Total Cost Averaged over the First 20 years of Production	Cost per Person Covered by Intervention	Cost per DALY Averted	Cost per Case Averted	Cost per Death Averted
Total Costs High Estimate	11,657,634	15,666,885	325,337	1,108,681	3.03	94.25	85.59	4,779
Total Costs Average Estimate	8,721,488	11,720,951	221,217	807,265	2.20	68.63	62.32	2,833
Total Costs Low Estimate	5,826,842	7,940,710	133,412	530,447	1.45	45.09	40.95	2,286
Post Research and Development Costs High Estimate	5,855,979	7,869,946	325,337	718,834	1.96	61.11	55.50	3,098
Post Research and Development Costs Average Estimate	4,419,833	5,939,886	221,217	518,211	1.41	44.05	40.01	2,234
Post Research and Development Costs Low Estimate	3,025,187	4,065,598	133,412	336,692	0.92	28.62	25.99	1,451
Annual Costs High Estimate	0	0	325,337	325,337	0.89	27.66	25.12	1,402
Annual Costs Average Estimate	0	0	221,217	221,217	0.60	18.81	17.08	954
Annual Costs Low Estimate	0	0	133,412	133,412	0.36	11.34	10.30	575

## Results

### Predicting epidemiological outcomes of pgSIT release

**Fig 2:**
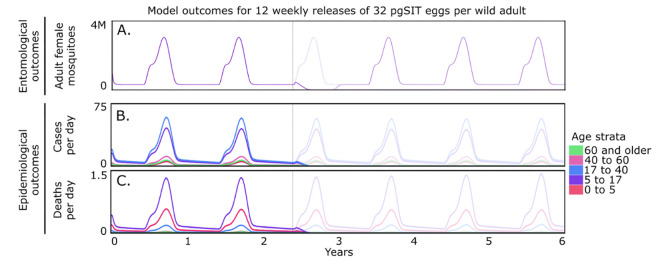
Model-predicted impact of pgSIT on entomological and epidemiological outcomes. 12 consecutive weekly releases of 32 pgSIT eggs per wild adult were simulated in a mosquito population resembling that of the Upper River region of The Gambia. Releases begin on June 1st of the third year and are denoted by a vertical line. Full model details are provided in the Methods section, and model parameters are provided in S1 Table. Model outcomes depicted include: (A) adult female mosquito density (purple); (B) age-stratified daily malaria cases (colors represent age strata as depicted in key), and (C) age-stratified daily deaths averted (age strata as depicted in key). Solid lines represent modeled pre and post-intervention dynamics, and low-opacity lines represent the case of no intervention (used to calculate cases and deaths averted).

Epidemiological modeling can predict the intervention-averted malaria cases and mortality needed to understand the impact of pgSIT on malaria transmission in the URR of The Gambia. With the parameterized modeling framework (described in the Methods section) in place, weekly releases of pgSIT *A. gambiae* eggs were simulated from the beginning of the rainy season (June 1st) for a variable number of weeks and release sizes. This exploratory analysis predicted a release scheme of ≥12 weekly releases of 32 pgSIT eggs per adult mosquito would effectively eliminate the URR *A. gambiae* population (Fig 2). In addition, in the first year following the beginning of releases (June 1st - May 31st), this led to 157 fewer cases and three fewer deaths (Table 1). The first year includes the period of releases, as well as cases and deaths averted that are not fully realized until the following years. In subsequent years (years 2–5 of the simulation), ~ 13,000 cases and ~230 deaths were simulated to have been averted per year across the URR (Table 1).

### Quantifying the costs of implementing pgSIT in The Gambia

We utilize a range (low, average, and high) of potential initial costs and annual costs to capture the full range of predicted costs and vary what is included in the costs, only annual costs (133,412–325,337 USD) or annual costs inclusive of all research and development costs (530,447–1,108,681 USD) (Table 2). The full methodology of the costs is elaborated on in Section 1 in S1 Text.

### Evaluating cost effectiveness in DALY, death, and cases averted

Cost per DALY averted ranges from 11.34 to 94.25 USD, and cost per case prevented ranges from 10.30 to 85.59 USD (Table 2). These averaged annual costs are also divided by the total deaths prevented to get the cost per life saved. The range of costs is included to provide several options to compare the cost per DALY saved and case averted to a variety of interventions at different stages of development.

### Quantifying the per person pgSIT coverage costs in the Upper River region of The Gambia

Dividing the annual estimated costs by the expected population in 2030, pgSIT is expected to cost 0.36 to 3.03 USD per person or less as the population increases.

## Discussion

By all evaluated measures, pgSIT is predicted to be cost-effective for preventing clinical malaria cases and malaria-associated deaths in URR, The Gambia. This program has a net gain within six years of operation and, by most metrics, much sooner (S30–S37 Tables). For example, the value of statistical life with income elasticity of 1.0-1.5, quality adjusted life years, or GDP-associated calculations show the investment is paid off by the second year of operation (S34–S37 Tables). While pgSIT is expected to provide increased coverage compared to current interventions, the annual cost of pgSIT is comparable to the current cost of LLINs and IRS interventions in the URR (S30 and S38 Tables), with the current interventions costing approximately 372,000 USD annually. Additionally, when considering the annual costs alone, pgSIT is currently expected to save lives and prevent disease at similar costs to current interventions (Table 2, S38 Table). Current vector control interventions cost 1.83 (LLINs) to 7.52 (IRS) USD per person, which is higher than pgSIT’s expected range (Table 2, S43 Table). These interventions save one life per 1,692.23 USD for LLINs and approximately 1,109.39 USD for IRS [48]. The cost per DALY averted is 58.75 USD for LLINs and 33.20 USD for IRS, comparable to pgSIT’s expected costs [48]. The cost per case averted for LLINs is 7.72 USD, which is lower than the lowest predicted cost per case averted for pgSIT. These costs to avert cases are likely underestimated, however, as the LLIN and IRS cost references had strict selection criteria requiring primary costing data and a focus on two papers with data for treating the most vulnerable individuals and, therefore, the most cost effective to treat [48–50]. As pgSIT cost is associated with the size of the mosquito population but is independent of human density, it is expected to be even more cost effective in regions with a low mosquito-to-human population ratio. In particular, urban areas typically have a lower mosquito-to-human ratio than rural areas, such as the URR [51–53].

Current interventions are cost-effective, but it is highly unlikely that these approaches could eliminate malaria in the URR, even with increased investment in these technologies [54–57]. Insecticide resistance will inevitably cause a decline in effectiveness. Alternative insecticides in the development pipeline are often more costly and, without proper management, may also rapidly become ineffective for vector control due to insecticide resistance [58]. Malaria vaccines have been developed for decades and have yet to reach efficacy that could lead to elimination [59]. Gene drive, while a promising technology, has several technical hurdles and safety concerns due to the release of self-replicating transgenes into the environment [60, 61].

Barring significant progress in other approaches, pgSIT is the genetic technology closest to field readiness in terms of technical and safety considerations. PgSIT also has a minimal environmental impact compared to conventional mosquito suppression technologies. PgSIT is species-specific or species complex-specific and will only target and suppress *A. gambiae*, or its species complex [62]. Many current control methods use insecticides, which can have off-target effects on beneficial insects and local biodiversity. Additionally, as mosquitoes become increasingly resistant to insecticides and require more intensive and rotating applications of insecticides to maintain the same effect, these negative off-target effects will only increase. After exhaustive testing and wide-scale implementation, pgSIT may be able to replace insecticidal methods if it meets the standard of care standards set by the WHO.

The pgSIT technology, while containing similar components to the archetypical CRISPR-based gene drive, is a safer alternative that may serve as a low-risk step toward implementing more invasive genetic technologies for mosquito control. PgSIT is a dead end for synthetic transgenes, whereby the sterile males do not produce viable offspring, and therefore, simply halting their release will rapidly eliminate their synthetic genes from the population. If a gene drive technology that spreads and persists in the environment is released in the area in the future, there will also be no pgSIT genes in the population to complicate the introduction of these technologies.

There are notable limitations to this analysis. Since there has not been a previous large-scale pgSIT production program and no similar program in The Gambia, there are unknown costs excluded from our estimates. To minimize unknowns, we based our estimated costs on other mosquito mass production programs [43,63,64] and used a range of cost estimates elaborated on in the supplementary information, but further scaling of pgSIT in field trials would better estimate these costs. These field trials will provide an opportunity to integrate the fluorescent sex sorting technology, SEPARATOR, with pgSIT and to assess its efficacy at scale [29–32]. It is important to note that the purpose of this early analysis is to estimate if this technology has the potential to be cost effective based on current available data, as further investment into this technology will be costly.

The current pgSIT suppression estimates are based on laboratory cage trials and fitness experiments [23], which impacts extrapolation to a large scale release program. However, this development pipeline fits the WHO guidelines for testing genetically modified mosquitoes intended for field release [34]. The next step is to conduct confined/small field population studies to assess pgSIT efficacy against wild populations, which will support more robust, predictive models of pgSIT performance in real-world scenarios. Similar technologies in development will also take this WHO approach, as it is a key stakeholder in recommending technologies for vector-borne disease control. Our cost estimates further derisk pgSIT development by facilitating step-wise investment even early in development with clearly defined time horizons for pgSIT performance, safety, and cost throughout the field trials and scaling of pgSIT. The series of trials required to approve genetic biocontrol technologies could guide investment time horizons. These trials have clear points to evaluate the technology’s success, and data from these trials could be used to re-evaluate the expected cost effectiveness [34]. These trials will additionally provide mass rearing and other insights that will apply to other genetically engineered mosquito interventions. Additionally, the model omitted the geography of the URR, assuming a closed, well-mixed population without migration. In the first years of the intervention, mosquitoes from outside the URR are likely to recolonize the treatment area and would require subsequent pgSIT releases for maintenance of suppression, albeit at reduced release sizes. Subsequent modeling efforts would be strengthened by data generated from pgSIT field trials and other mosquito suppression efforts in the URR. While this model is likely to change with further data, this preliminary assessment is important to evaluate the range of expected costs to determine if this technology is cost effective and, therefore, worth pursuing.

Localized suppression of the primary malaria vector in The Gambia, *A. gambiae*, should have the greatest impact on malaria transmission *Anopheles gambiae* is part of a species complex, several of which are secondary vectors for malaria and may assume niches left in the absence of *A. gambiae*. Members of this species complex have been shown to hybridize, so pending testing to confirm pgSIT males may mate and suppress other species in this complex [65–68]. Notably, with the similar genetics of species in this complex and the flexibility of pgSIT design, pgSIT can easily be developed to control other malaria vectors in this species complex if the species is amenable to colonization and scaling. PgSIT, therefore, could be an important strategy to control more outdoor biting malaria vectors in this complex, such as *Anopheles arabiensis*, that rarely encounter conventional interventions such as LLINS and IRS [69].

A pgSIT *A. gambiae* mass production facility would only be at full capacity for approximately 12–18 weeks [46]. The remaining 34–40 weeks of the year can be leveraged for other purposes, including producing different mosquito species or control technologies. This could support the mass rearing of pgSIT technologies to control the dengue vector, *Aedes aegypti*, which has eggs that can be stockpiled to release in The Gambia or for export elsewhere to curtail future dengue outbreaks and provide additional program revenue. Previous pgSIT modeling studies [27,38] suggested that local *Aedes* mosquito populations could be eliminated by ~10–24 consecutive weekly releases of ~40–400 pgSIT eggs per wild adult. Therefore, a dual *Aedes* and *Anopheles* production program could easily be managed in one facility. The facility could also be used to produce more invasive interventions, such as gene drives. Therefore, while cost-effective and beneficial to malaria prevention in the URR, the pgSIT program could also support the prevention of other diseases in the URR and beyond.

Overall, this initial cost assessment based on the available data suggests that the use of pgSIT technology against *Anopheles gambiae* in the URR of The Gambia could be a cost effective intervention. The current estimates are on par with the cost of current interventions and are based on preventing more malaria infections and deaths beyond the current interventions used in the region. This estimate suggests that investment into the development and implementation of field trials could be worthwhile as the technology is expected to be an affordable method to further prevent malaria in this region. While we expect further research and development, especially implementation in field trials, to provide new data that will refine the estimate, providing this early estimate is a tool to ensure that technologies have the potential to be viable upon implementation.

## Supporting information

S1 Fig Seasonal rainfall profile for Upper River region, The Gambia. Points represent mean daily rainfall measurements (in mm) for the three years between January 1st, 2017 and December 31st, 2019. The solid line represents the seasonal rainfall profile, fitted using the umbrella package in R (https://github.com/mrc-ide/umbrella). This is used to calculate the time-varying environmental carry capacity for larvae in the life history module of MGDrivE 3. (TIF)

S2 Fig **Phased testing pathway for genetically modified mosquitoes.** This figure was based on guidelines and a figure by WHO [60]. Figure generated in BioRender.com.
(TIF)

S3 Fig **Mass rearing during** **the facility’s active phase.** The general process of mass rearing Anopheles gambiae mosquitoes when the facility is actively producing mosquitoes for release is shown. Generating pgSIT sterile males (Factory Stages)- This begins with hatching the Cas9 and gRNA parent lines (A-B). Assuming that COPAS is used, sex sorting occurs at the L1 larval stage by sex-specific fluorescent markers (C). If Senecio Robotics (or the Verily method) is used, sorting occurs at early adult emergence (pupal isolation and adult cage D-E). Following the crossing of these lines, offspring larvae are mass-reared in trays for seven to nine days. On days seven to nine, pupae are isolated from the trays and transferred to adult-rearing cages (or to a screening cage for the Senecio Robotics technology sex sorting approach) (D-E). Males from the Cas9 line and females from the gRNA line will mate ad libitum and acclimate for three days (E). Mosquitoes are then blood-fed by an artificial Hemotek feeder or by a similar method (Section 2.1.3.9) (F). Two days post blood feeding, water is added to the cage trough for egg laying. The following day, the eggs are harvested and distributed to the field (G). The pure-bred lines are used to create the next generation of the parental line, and this repeats the cycle at the facility (G). Maintenance and Ramping Phases have the same Factory Stages and do not have Release Stages. (Release Stages)- The egg delivery to the release sites will be done by drone or other vehicles (H). Once distributed in the field, the larvae will be raised in shallow trays to adulthood, when they mate with wild female mosquitoes (I). This Active Phase production is continued for 12 weeks whereby modeling predicts localized extinction of A. gambiae (J). Figure generated in BioRender.com.
(TIF)

S1 Table Parameters used in mathematical modeling.
(DOCX)

S2 Table **Annual cost of introgression experiments. **Cost sourcing in Section 1.3.9a.
(DOCX)

S3 Table **Total budget estimate of introgression and initial cage trials.** Costing source discussed in Section 1.1.3.
(DOCX)

S4 Table **Total budget estimate of** **small scale trial.** Utilizes the same cost sources as Table S3.
(DOCX)

S5 Table **Total budget estimate for large scale field trials.** Utilizes the same cost sources as Table S3.
(DOCX)

S6 Table **Monitoring costs for the first 5 years of mosquito releases.** Utilizes the same cost sources as Table S3.
(DOCX)

S7 Table **Total adult mosquitoes required for egg production.** The first column shows the daily mosquito egg production requirements needed for releases in the URR. The daily calculations are derived from the 60.8 million egg per week estimate divided daily across the week. The second column accounts for fecundity variations in mass rearing conditions. We expect to see at least 300 eggs produced per female. If we have a 30% decrease in production, we expect 210 eggs per female. The number of adult females needed to meet the daily requirement is calculated by dividing the daily eggs by fecundity. At a 1:1 male:female mating ratio [15], we double the adult female number to get the total number of adult mosquitoes.
(DOCX)

S8 Table **COPAS FP 500 larvae daily rearing requirements.** The larvae rearing numbers are based on the assumption that sex sorting is done on newly emerged L1 larvae.
(DOCX)

S9 Table **Total initial costs with least expensive trials.** This cost assessment is based on the direct cost estimates of trials based on assumed reagent requirements, wages for local employees in The Gambia and other expected expenses. The Upper River Rearing Costs assumes the need to prepare these sites with a trial run. A high cost can be seen in Table S10.
(DOCX)

S10 Table **Total initial costs with more expensive trials.** This table includes additional costs associated with the larger and more expensive field trials and additional machines for sex sorting. The additional field trial cost are: 1) 500,000 USD per year for the five year monitoring period, 2) an additional 2.5 million USD for the larger scale cluster randomized trial (approximate cost of a previous large scale trial [6] as per communication with Umberto D’Alessandro) and 3) 500,000 USD to pay for a social science team to manage public communication of pgSIT technology. As a facility of this scale has not been developed, and extensive testing has not yet been conducted with these machines, this may need to be tested. These estimates should represent the upper limit of initial costs, but there are significant uncertainties on whether these expenditures will be necessary. Cost data provided in Supporting Text.
(DOCX)

S11 Table **Cost of COPAS FP 500 and annual service fee.** Cost data provided as preliminary quotes from Union Biometrica.
(DOCX)

S12 Table **COPAS sex sorting rack and tray numbers, cost and expected maintenance fees.** Cost data provided as preliminary quotes from Wolbaki Ltd and Vienna Scientific.
(DOCX)

S13 Table **Annual water usage and cost. **Cost data provided in Supporting Text.
(DOCX)

S14 Table Cost per liter of larval food.
(DOCX)

S15 Table **Larval food requirements and cost. **Utilizes Cost Data from Table S14.
(DOCX)

S16 Table **Adult mosquito cage costs.** Cost data from Maïga et al.
(DOCX)

S17 Table **Fresh blood estimate: utilizing locally sourced blood.** Pricing is based on blood obtained from food markets in the US(Supplier: B&R Food Service, Product: “BEEF BLOOD FROZEN 6 GALLON CASE AMERICAN”). It is expected that fresh blood costs in The Gambia will be less than US prices due to lack of a market for it in The Gambia.
(DOCX)

S18 Table **Hemotek device cost and annual fees.** Cost data from Hemotek website (Accessed 2023).
(DOCX)

S19 Table **Drone costs and annual fees.** Cost data provided from a preliminary quote from Arda Impact.
(DOCX)

S20 Table **The costs of rearing larvae in The Upper River region. **Cost data based on current costs of training and public outreach in The Gambia for current interventions, water and food costs described in Section 1.3.9a and Table S14, and Labor Costs are described in Section 1.2.13b and were considered to be sufficient to maintain part time managers.
(DOCX)

S21 Table **Mutation rates and quality control. **The background mutation rate is based on estimates from another member of the Anopheles gambiae species complex, Anopheles coluzzi [38]. To estimate the expected mutation rate per generation, we use estimates of the genetic element base lengths and the total number of mosquitoes per generation. These calculations can then be used to determine the mutation rate over time in the maintenance and active phase, and the expected mutation rate in the Cas9-gRNA pgSIT offspring.
(DOCX)

S22 Table **Monitoring costs for the first 5 years of mosquito releases. **Cost data was based on salaries discussed with Umberto D’Alesandro and budgeting of minimal equipment. A larger budget was estimated for this work and applied for the high cost.
(DOCX)

S23 Table **Banjul Land Cost Estimate.** Survey of available property on AccessGambia near Banjul with costs converted to USD to determine average cost per square meter.
(DOCX)

S24 Table **Minimum facility size, land, and cost. **Land requirements were estimated as described in Section 1.2.16a. Cost data was estimated by averaging cost per square meter of land based on available plots of land on The Gambian government’s website near Banjul.
(DOCX)

S25 Table **High wage annual estimate.** GMD- Gambian dalasi. Wages estimated via communication with Umberto D’Alesandro and PayLab survey information retrieved in 2023. High, average and low estimates from these surveys were utilized below in Tables S25-S27 respectively.
(DOCX)

S26 Table Medium wage annual estimate.
(DOCX)

S27 Table Low wage annual estimate.
(DOCX)

S28 Table **Total initial costs with least expensive trials. **This cost assessment is based on the direct cost estimates of trials based on assumed reagent requirements, wages for local employees in The Gambia and other expected expenses. The Upper River Rearing Costs assumes the need to prepare these sites with a trial run. A high cost can be seen in Table S29.
(DOCX)

S29 Table **Total initial costs with more expensive trials.** This table includes additional costs associated with the larger and more expensive field trials and additional machines for sex sorting. The additional field trial cost are: 1) 500,000 USD per year for the five year monitoring period, 2) an additional 2.5 million USD for the larger scale cluster randomized trial (approximate cost of a previous large scale trial [6] as per communication with Umberto D’Alessandro) and 3) 500,000 USD to pay for a social science team to manage public communication of pgSIT technology. As a facility of this scale has not been developed, and extensive testing has not yet been conducted with these machines, this may need to be tested. These estimates should represent the upper limit of initial costs, but there are significant uncertainties on whether these expenditures will be necessary.
(DOCX)

S30 Table **Total annual costs. **The total annual cost includes facility costs, including maintenance fees for the equipment and the high estimated cost in The Gambia, for labor, local resources and rearing in the URR. Rearing costs may be overestimated, however, as other malaria interventions rely on some volunteer labor. Maintenance of larvae at sites in the URR may also utilize other more affordable, local resources rather than imported mosquito feed.
(DOCX)

S31 Table Estimated age stratified life years saved.
(DOCX)

S32 Table Life years saved annually.
(DOCX)

S33 Table Sick days prevented by age over four years of pgSIT interventions.
(DOCX)

S34 Table Value of statistical life calculations.
(DOCX)

S35 Table Quality adjusted life year calculations.
(DOCX)

S36 Table GDP growth estimate.
(DOCX)

S37 Table Value of sick days saved.
(DOCX)

S38 Table Annualized mean costs of the interventions against malaria in the Upper River Region (in 2021 USD).
(DOCX)

S39 Table **Costs associated with malaria treatment seeking.** Adapted from [55] with conversions from British pound (GBP) to USD.
(DOCX)

S40 Table Treatment seeking costs saved from preventing malaria cases.
(DOCX)

S41 Table Population Estimate and Willingness-to-pay for malaria prevention in the URR.
(DOCX)

S42 Table Current malaria intervention costs in The Gambia.
(DOCX)

S43 Table **Cost per Case, DALY and Death Averted and Cost per Person Covered with current Interventions.** These values are derived from Conteh et al and converted to 2022 USD to be directly comparable.
(DOCX)

S1 Text (DOCX)
