# Supplementary figures and images for: Evaluating the cost of malaria elimination by Anopheles gambiae precision guided SIT in the Upper River region, The Gambia
Source: PLOS Glob Public Health. 2025 Jul 18;5(7):e0004903. doi: 10.1371/journal.pgph.0004903 (PMC12273942; doi:10.1371/journal.pgph.0004903)

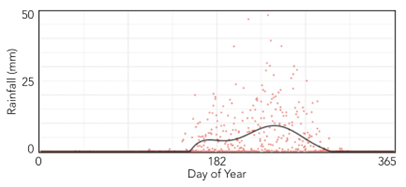

Supplement: S1 Fig — (TIF) [file pgph.0004903.s001.tif]

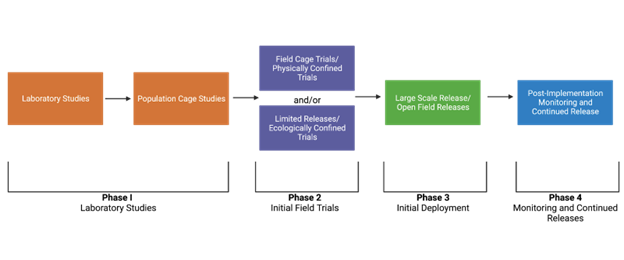

Supplement: S2 Fig — Phased testing pathway for genetically modified mosquitoes. This figure was based on guidelines and a figure by WHO [60]. Figure generated in BioRender.com. (TIF) [file pgph.0004903.s002.tif]

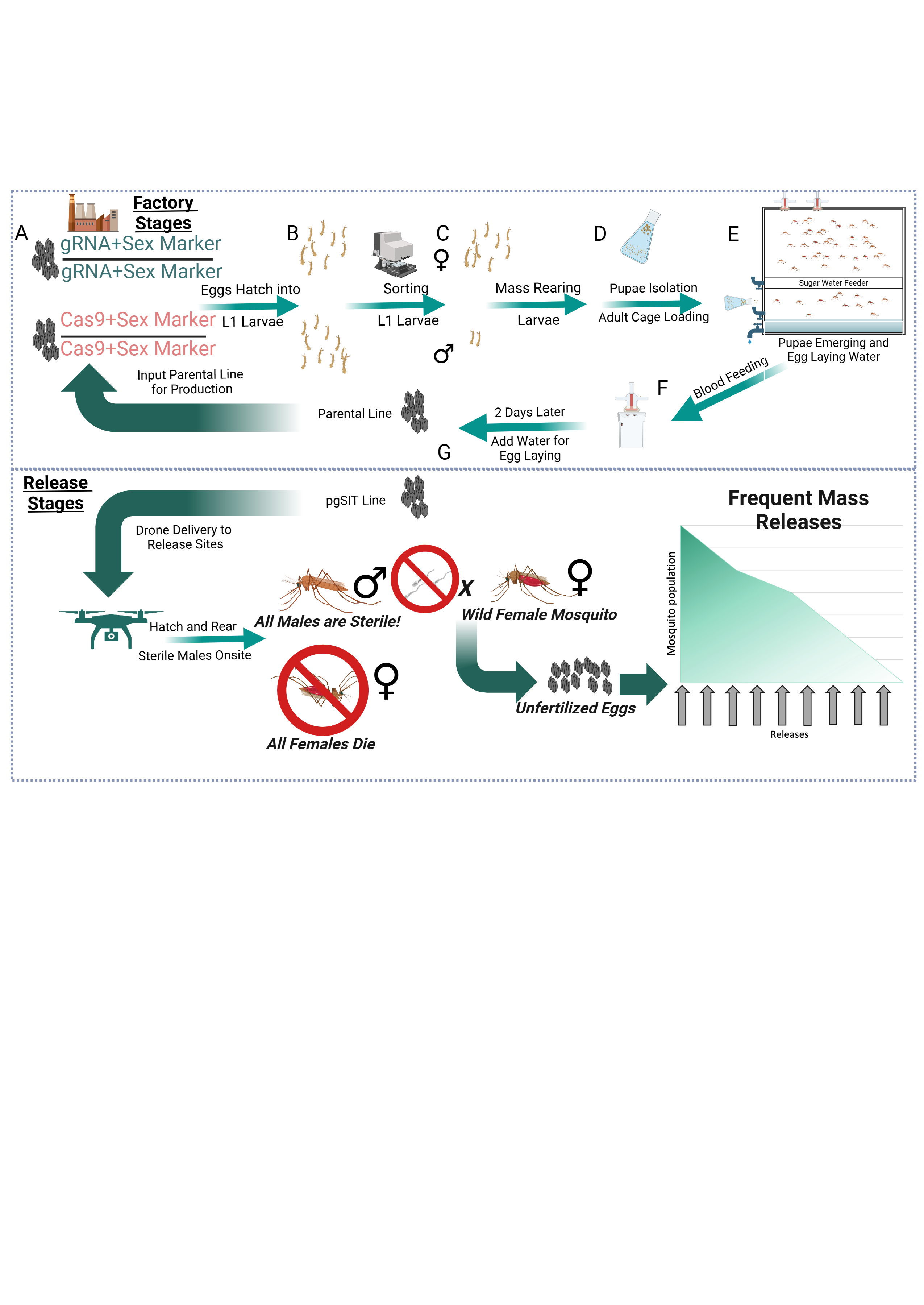

Supplement: S3 Fig — Mass rearing during the facility’s active phase. The general process of mass rearing Anopheles gambiae mosquitoes when the facility is actively producing mosquitoes for release is shown. Generating pgSIT sterile males (Factory Stages)- This begins with hatching the Cas9 and gRNA parent lines (A-B). Assuming that COPAS is used, sex sorting occurs at the L1 larval stage by sex-specific fluorescent markers (C). If Senecio Robotics (or the Verily method) is used, sorting occurs at early adult emergence (pupal isolation and adult cage D-E). Following the crossing of these lines, offspring larvae are mass-reared in trays for seven to nine days. On days seven to nine, pupae are isolated from the trays and transferred to adult-rearing cages (or to a screening cage for the Senecio Robotics technology sex sorting approach) (D-E). Males from the Cas9 line and females from the gRNA line will mate ad libitum and acclimate for three days (E). Mosquitoes are then blood-fed by an artificial Hemotek feeder or by a similar method (Section 2.1.3.9) (F). Two days post blood feeding, water is added to the cage trough for egg laying. The following day, the eggs are harvested and distributed to the field (G). The pure-bred lines are used to create the next generation of the parental line, and this repeats the cycle at the facility (G). Maintenance and Ramping Phases have the same Factory Stages and do not have Release Stages. (Release Stages)- The egg delivery to the release sites will be done by drone or other vehicles (H). Once distributed in the field, the larvae will be raised in shallow trays to adulthood, when they mate with wild female mosquitoes (I). This Active Phase production is continued for 12 weeks whereby modeling predicts localized extinction of A. gambiae (J). Figure generated in BioRender.com. (TIF) [file pgph.0004903.s003.tif]
