# Supplementary material for: Evaluating the cost of malaria elimination by Anopheles gambiae precision guided SIT in the Upper River region, The Gambia
Source: PLOS Glob Public Health. 2025 Jul 18;5(7):e0004903. doi: 10.1371/journal.pgph.0004903 (PMC12273942; doi:10.1371/journal.pgph.0004903)
Supplement: S1 Table — Parameters used in mathematical modeling. (DOCX) [file pgph.0004903.s004.docx]

#### S1 Table Parameters used in mathematical modeling

| **Parameter** | **Value** | **Reference** |
| --- | --- | --- |
| **pgSIT construct:** | | |
| pgSIT male fertility | 0.0 | [[1]](https://paperpile.com/c/JoQtIv/ZftFg) |
| pgSIT female viability | 0.0 | [[1]](https://paperpile.com/c/JoQtIv/ZftFg) |
| pgSIT male mating competitiveness (relative to wild-type) | 0.75 | [[1]](https://paperpile.com/c/JoQtIv/ZftFg) |
| pgSIT lifespan (relative to wildtype) | 0.75 | [[1]](https://paperpile.com/c/JoQtIv/ZftFg) |
| ***Anopheles gambiae* bionomics:** | | |
| Egg production per adult female (day^-1^) | 21 | [[57]](https://paperpile.com/c/JoQtIv/vTwZM) |
| Mean duration of egg stage (days) | 3 | [[58]](https://paperpile.com/c/JoQtIv/lSyJJ) |
| Mean duration of larval stage (days) | 7 | [[59–61]](https://paperpile.com/c/JoQtIv/Whjyf%2B2l9vD%2BjaYn1) |
| Mean duration of pupa stage (days) | 1 | [[59]](https://paperpile.com/c/JoQtIv/Whjyf) |
| Egg stage mortality rate (day^-1^) | 0.05 | [[62]](https://paperpile.com/c/JoQtIv/MiB4O) |
| Larval stage mortality rate (day^-1^) | 0.15 | [[62]](https://paperpile.com/c/JoQtIv/MiB4O) |
| Pupal stage mortality rate (day^-1^) | 0.05 | [[62]](https://paperpile.com/c/JoQtIv/MiB4O) |
| Carrying capacity of environment for larvae | (time-varying) | [[59,63]](https://paperpile.com/c/JoQtIv/Whjyf%2BiRkOI) |
| Mortality rate of adult mosquitoes (day^-1^) | 0.132 | [[64]](https://paperpile.com/c/JoQtIv/6t8p) |
| **Malaria transmission (Upper River region, The Gambia):** | | |
| Malaria prevalence at peak of rainy season | 18% | [[6]](https://paperpile.com/c/JoQtIv/rcYb) |
| Proportion of larval breeding sites available in dry season | 10% | [[65]](https://paperpile.com/c/JoQtIv/HZZ7c) |
| LLIN coverage | 55% | [[66]](https://paperpile.com/c/JoQtIv/wcAyh) |
| IRS coverage | 52% | [[66]](https://paperpile.com/c/JoQtIv/wcAyh) |
| ACT coverage | 50% | [[66]](https://paperpile.com/c/JoQtIv/wcAyh) |
