# Supplementary material for: Evaluating the cost of malaria elimination by Anopheles gambiae precision guided SIT in the Upper River region, The Gambia
Source: PLOS Glob Public Health. 2025 Jul 18;5(7):e0004903. doi: 10.1371/journal.pgph.0004903 (PMC12273942; doi:10.1371/journal.pgph.0004903)
Supplement: S2 Table — Annual cost of introgression experiments. Cost sourcing in Section 1.3.9a. (DOCX) [file pgph.0004903.s005.docx]

#### S2 Table: Annual cost of introgression experiments

Cost sourcing in Section 1.3.9a.

| **Introgression Materials** | **Liters per week** | **Cost per Week USD** | **Annual Cost USD** |
| --- | --- | --- | --- |
| **Water** | 250 | 63 | 3,250 |
| **Larvae Feed** | 27 | 0.69 | 36 |
| **Blood** | 0.3 | 0.75 | 39 |
| **Total Cost** |  |  | 3,325 |
