# Supplementary material for: Evaluating the cost of malaria elimination by Anopheles gambiae precision guided SIT in the Upper River region, The Gambia
Source: PLOS Glob Public Health. 2025 Jul 18;5(7):e0004903. doi: 10.1371/journal.pgph.0004903 (PMC12273942; doi:10.1371/journal.pgph.0004903)
Supplement: S3 Table — Total budget estimate of introgression and initial cage trials. Costing source discussed in Section 1.1.3. (DOCX) [file pgph.0004903.s006.docx]

#### S3 Table: Total budget estimate of introgression and initial cage trials

Costing source discussed in Section 1.1.3.

| **Cost Category** | **Annual Cost** | **Total** | **Cost Source** |
| --- | --- | --- | --- |
| **Facility Rental** | 100,000 | 300,000 | Per communication with Umberto D’Alesandro |
| **Research Fellow** | 71,000 | 213,000 | Per communication with Umberto D’Alesandro |
| **Technicians** | 13,500 | 121,500 | Per communication with Umberto D’Alesandro |
| **Cages** | 250 | 1,000 | [[5]](https://paperpile.com/c/JoQtIv/UsiJ) |
| **Wolbaki Mass Rearing Racks** | 22,500  (Initial Purchase only) | 22,500 | Quote Provided by Wolbaki |
| **Fluorescent Microscope** | 10,000 | 10,000 | Range of microscopes reviewed, sub 10,000 USD viable when looking at refurbished models through Microscope Central |
| **Experiment Budget** | 150,000 | 300,000 | Per communication with Umberto D’Alesandro |
| **Mosquito Rearing Input** | 3,325 | 9,975 | Table S2 |
| **Total** | 370,575 | 977,975 |  |
