# Supplementary material for: Evaluating the cost of malaria elimination by Anopheles gambiae precision guided SIT in the Upper River region, The Gambia
Source: PLOS Glob Public Health. 2025 Jul 18;5(7):e0004903. doi: 10.1371/journal.pgph.0004903 (PMC12273942; doi:10.1371/journal.pgph.0004903)
Supplement: S4 Table — Total budget estimate of small scale trial. Utilizes the same cost sources as Table S3. (DOCX) [file pgph.0004903.s007.docx]

#### S4 Table: Total budget estimate of small scale trial

Utilizes the same cost sources as Table S3.

| **Cost Category** | **Annual Cost** | **Total** |
| --- | --- | --- |
| **Facility Rental** | 100,000 | 200,000 |
| **Research Fellow** | 71,000 | 142,000 |
| **Technicians** | 13,500 | 81,000 |
| **COPAS FP 500** | 308,000* | 344,960 |
| **Part time Workers for Release Season** | 4,500 | 9,000 |
| **Experiment Budget** | 100,000 | 200,000 |
| **Mosquito Rearing Input** | 3,325 | 6,650 |
| **Total** | 600,325 | 983,610 |

* One time purchase of a COPAS sorting machine. The total costs include maintenance fees that are expected.
