# Supplementary material for: Evaluating the cost of malaria elimination by Anopheles gambiae precision guided SIT in the Upper River region, The Gambia
Source: PLOS Glob Public Health. 2025 Jul 18;5(7):e0004903. doi: 10.1371/journal.pgph.0004903 (PMC12273942; doi:10.1371/journal.pgph.0004903)
Supplement: S6 Table — Monitoring costs for the first 5 years of mosquito releases. Utilizes the same cost sources as Table S3. (DOCX) [file pgph.0004903.s009.docx]

#### Table S6: Monitoring costs for the first 5 years of mosquito releases

Utilizes the same cost sources as Table S3.

| **Monitoring Staff and Costs** | **Annual** | **Total** |
| --- | --- | --- |
| **Research Fellow** | 71,000 | 355,000 |
| **Technicians** | 13,500 | 202,500 |
| **Seasonal Workers** | 9,000 | 45,000 |
| **Monitoring Budget** | 30,000 | 150,000 |
| **Total** | 123,500 | 752,500 |
