# Supplementary material for: Evaluating the cost of malaria elimination by Anopheles gambiae precision guided SIT in the Upper River region, The Gambia
Source: PLOS Glob Public Health. 2025 Jul 18;5(7):e0004903. doi: 10.1371/journal.pgph.0004903 (PMC12273942; doi:10.1371/journal.pgph.0004903)
Supplement: S7 Table — Total adult mosquitoes required for egg production. The first column shows the daily mosquito egg production requirements needed for releases in the URR. The daily calculations are derived from the 60.8 million egg per week estimate divided daily across the week. The second column accounts for fecundity variations in mass rearing conditions. We expect to see at least 300 eggs produced per female. If we have a 30% decrease in production, we expect 210 eggs per female. The number of adult females needed to meet the daily requirement is calculated by dividing the daily eggs by fecundity. At a 1:1 male:female mating ratio [15], we double the adult female number to get the total number of adult mosquitoes. (DOCX) [file pgph.0004903.s010.docx]

#### S7 Table: Total adult mosquitoes required for egg production.

The first column shows the daily mosquito egg production requirements needed for releases in the URR. The daily calculations are derived from the 60.8 million egg per week estimate divided daily across the week. The second column accounts for fecundity variations in mass rearing conditions. We expect to see at least 300 eggs produced per female. If we have a 30% decrease in production, we expect 210 eggs per female. The number of adult females needed to meet the daily requirement is calculated by dividing the daily eggs by fecundity. At a 1:1 male:female mating ratio [[15]](https://paperpile.com/c/JoQtIv/JPcz), we double the adult female number to get the total number of adult mosquitoes.

| **Daily Mosquito Eggs Required** | **Fecundity per Female Lifespan** | **Female Adult Mosquitoes Required Daily** | **Total Adult Mosquitoes Required Daily** |
| --- | --- | --- | --- |
| 8,686,000 | 300 | 28,953 | 57,906 |
| 8,686,000 | 210 | 41,362 | 82,723 |
