# Supplementary material for: Evaluating the cost of malaria elimination by Anopheles gambiae precision guided SIT in the Upper River region, The Gambia
Source: PLOS Glob Public Health. 2025 Jul 18;5(7):e0004903. doi: 10.1371/journal.pgph.0004903 (PMC12273942; doi:10.1371/journal.pgph.0004903)
Supplement: S8 Table — COPAS FP 500 larvae daily rearing requirements. The larvae rearing numbers are based on the assumption that sex sorting is done on newly emerged L1 larvae. (DOCX) [file pgph.0004903.s011.docx]

#### S8 Table: COPAS FP 500 larvae daily rearing requirements.

The larvae rearing numbers are based on the assumption that sex sorting is done on newly emerged L1 larvae.

| **Conditions** | **Fecundity** | **Survival** | **Mosquito Larvae Required for Production** | **Total Mosquito Larvae Required** |
| --- | --- | --- | --- | --- |
| **High Fecundity, High Survival** | 300 | 75% | 77,208 | 81,840 |
| **Low Fecundity, High Survival** | 210 | 75% | 110,297 | 116,915 |
| **High Fecundity, Low Survival** | 300 | 50% | 115,812 | 122,761 |
| **Low Fecundity, Low Survival** | 210 | 50% | 165,446 | 175,394 |
