# Supplementary material for: Evaluating the cost of malaria elimination by Anopheles gambiae precision guided SIT in the Upper River region, The Gambia
Source: PLOS Glob Public Health. 2025 Jul 18;5(7):e0004903. doi: 10.1371/journal.pgph.0004903 (PMC12273942; doi:10.1371/journal.pgph.0004903)
Supplement: S11 Table — Cost of COPAS FP 500 and annual service fee. Cost data provided as preliminary quotes from Union Biometrica. (DOCX) [file pgph.0004903.s014.docx]

#### S11 Table: Cost of COPAS FP 500 and annual service fee

Cost data provided as preliminary quotes from Union Biometrica.

| **Conditions** | **Daily Number of Larvae Required** | **Number of COPAS**  **Machines Needed** | **Total COPAS**  **Needed (with spare)** | **Equipment Cost (Machines + Laser Spares) USD** | **Annual Service Fee* USD** | **Total Upfront Cost^#^ USD** |
| --- | --- | --- | --- | --- | --- | --- |
| **High Fecundity, High Survival** | 81,840 | 0.15 | 2 | 759,072 | 91,089 | 948,840 |
| **Low Fecundity, High Survival** | 116,915 | 0.21 | 2 | 759,072 | 91,089 | 948,840 |
| **High Fecundity, Low Survival** | 122,761 | 0.22 | 2 | 759,072 | 91,089 | 948,840 |
| **Low Fecundity, Low Survival** | 175,394 | 0.31 | 2 | 759,072 | 91,089 | 948,840 |

* Annual maintenance/service fee is 12% of initial machine costs. Additional spare laser costs are excluded from the annual maintenance/service fee calculations.

^#^ Total upfront costs = equipment cost + annual service fee + 25% estimated import/taxes on the equipment.
