# Supplementary material for: Evaluating the cost of malaria elimination by Anopheles gambiae precision guided SIT in the Upper River region, The Gambia
Source: PLOS Glob Public Health. 2025 Jul 18;5(7):e0004903. doi: 10.1371/journal.pgph.0004903 (PMC12273942; doi:10.1371/journal.pgph.0004903)
Supplement: S12 Table — COPAS sex sorting rack and tray numbers, cost and expected maintenance fees. Cost data provided as preliminary quotes from Wolbaki Ltd and Vienna Scientific. (DOCX) [file pgph.0004903.s015.docx]

#### S12 Table: COPAS sex sorting rack and tray numbers, cost and expected maintenance fees

Cost data provided as preliminary quotes from Wolbaki Ltd and Vienna Scientific.

| **Conditions** | **Daily Number of Larvae Sorted** | **Daily Racks** | **Total Racks in Use** | **Total Racks (Round Up)** | **Wolbaki Cost USD** | **Wolbaki Annual 1% Maintenance Cost USD** | **Vienna Scientific Cost USD** | **Vienna Scientific Annual 1% Maintenance Cost USD** |
| --- | --- | --- | --- | --- | --- | --- | --- | --- |
| **High Fecundity, High Survival Calculation** | 81,840 | 0.15 | 1.2 | 2 | 45,000 | 450 | 76,000 | 760 |
| **Low Fecundity, High Survival Calculation** | 116,915 | 0.21 | 1.7 | 2 | 45,000 | 450 | 76,000 | 760 |
| **High Fecundity, Low Survival Calculation** | 122,761 | 0.23 | 1.8 | 3 | 67,500 | 675 | 114,000 | 1,140 |
| **Low Fecundity, Low Survival Calculation** | 175,394 | 0.32 | 2.61 | 3 | 67,500 | 675 | 114,000 | 1,140 |
