# Supplementary material for: Evaluating the cost of malaria elimination by Anopheles gambiae precision guided SIT in the Upper River region, The Gambia
Source: PLOS Glob Public Health. 2025 Jul 18;5(7):e0004903. doi: 10.1371/journal.pgph.0004903 (PMC12273942; doi:10.1371/journal.pgph.0004903)
Supplement: S13 Table — Annual water usage and cost. Cost data provided in Supporting Text. (DOCX) [file pgph.0004903.s016.docx]

#### S13 Table: Annual water usage and cost

Cost data provided in Supporting Text.

| **Conditions** | **Maintenance Phase Water Used Liters** | **Total Racks Used** | **Water Usage per Active+ Ramping Phase Liters** | **Annual Water Usage Liters** | **Annual Cost USD** |
| --- | --- | --- | --- | --- | --- |
| **COPAS Sorting, High**  **Fecundity, High Survival** | 18,020 | 2 | 19,080 | 37,100 | 371 |
| **COPAS**  **Sorting, Low Fecundity, High Survival** | 18,020 | 2 | 19,080 | 37,100 | 371 |
| **COPAS**  **Sorting, High Fecundity, Low Survival** | 18,020 | 3 | 28,620 | 46,640 | 466 |
| **COPAS**  **Sorting, Low Fecundity, Low Survival** | 18,020 | 3 | 28,620 | 46,640 | 466 |
