# Supplementary material for: Evaluating the cost of malaria elimination by Anopheles gambiae precision guided SIT in the Upper River region, The Gambia
Source: PLOS Glob Public Health. 2025 Jul 18;5(7):e0004903. doi: 10.1371/journal.pgph.0004903 (PMC12273942; doi:10.1371/journal.pgph.0004903)
Supplement: S14 Table — Cost per liter of larval food. (DOCX) [file pgph.0004903.s017.docx]

#### S14 Table: Cost per liter of larval food

| **Raw Material Costs** | **USD** | **Cost per Liter USD** | **Citation** |
| --- | --- | --- | --- |
| **Tuna Meal cost per 100 kg** | 80 | 0.0056 | [[19,67]](https://paperpile.com/c/JoQtIv/ex1X%2BzfZK) (Supplier:  T.C. Union Agrotech,  Product: Tuna Meal) |
| **Brewer's Yeast per 100 kg** | 1000 | 0.015 | [[19,22]](https://paperpile.com/c/JoQtIv/ex1X%2BM7OD)(Supplier MP Biomedicals, Model: 0290331280) |
| **Chickpea flour per 100 kg** | 328.26 | 0.0049 | [[19]](https://paperpile.com/c/JoQtIv/ex1X)(Supplier: Bulk Foods, Model: 40042) |
| **Total:** |  | 0.0255 |  |
