# Supplementary material for: Evaluating the cost of malaria elimination by Anopheles gambiae precision guided SIT in the Upper River region, The Gambia
Source: PLOS Glob Public Health. 2025 Jul 18;5(7):e0004903. doi: 10.1371/journal.pgph.0004903 (PMC12273942; doi:10.1371/journal.pgph.0004903)
Supplement: S15 Table — Larval food requirements and cost. Utilizes Cost Data from Table S14. (DOCX) [file pgph.0004903.s018.docx]

#### S15 Table: Larval food requirements and cost

Utilizes Cost Data from Table S14.

| **Conditions** | **Maintenance Phase Food Used Liters** | **Total Racks Used** | **Food Usage per Active+ Ramping Phase Liters** | **Annual Food Usage Liters** | **Cost USD** |
| --- | --- | --- | --- | --- | --- |
| **COPAS**  **Sorting, High Fecundity, High Survival** | 5,100 | 2 | 3,600 | 8,700 | 222 |
| **COPAS Sorting, Low Fecundity, High Survival** | 5,100 | 2 | 3,600 | 8,700 | 222 |
| **COPAS Sorting, High Fecundity, Low Survival** | 5,100 | 3 | 5,400 | 10,500 | 268 |
| **COPAS Sorting, Low Fecundity, Low Survival** | 5,100 | 3 | 5,400 | 10,500 | 268 |
