# Supplementary material for: Evaluating the cost of malaria elimination by Anopheles gambiae precision guided SIT in the Upper River region, The Gambia
Source: PLOS Glob Public Health. 2025 Jul 18;5(7):e0004903. doi: 10.1371/journal.pgph.0004903 (PMC12273942; doi:10.1371/journal.pgph.0004903)
Supplement: S16 Table — Adult mosquito cage costs. Cost data from Maïga et al. (DOCX) [file pgph.0004903.s019.docx]

#### S16 Table: Adult mosquito cage costs

Cost data from Maïga et al.

| **Fecundity Rate** | **Daily Adults loaded into Cages** | **Daily Cage Usage Round-Up** | **Total Cages Needed** | **Maïga et al. Unit Cost Total** | **Maïga et al. Unit Annual Maintenance Fees Total** | **Commercial Unit Cost Total** | **Commercial Unit Annual Maintenance Fees Total** |
| --- | --- | --- | --- | --- | --- | --- | --- |
| **High** | 57,906 | 4 | 60 | 15,000 | 750 | 150,000 | 7,500 |
| **Low** | 82,723 | 6 | 90 | 22,500 | 1,125 | 225,000 | 11,250 |
