# Supplementary material for: Evaluating the cost of malaria elimination by Anopheles gambiae precision guided SIT in the Upper River region, The Gambia
Source: PLOS Glob Public Health. 2025 Jul 18;5(7):e0004903. doi: 10.1371/journal.pgph.0004903 (PMC12273942; doi:10.1371/journal.pgph.0004903)
Supplement: S17 Table — Fresh blood estimate: utilizing locally sourced blood. Pricing is based on blood obtained from food markets in the US(Supplier: B&R Food Service, Product: “BEEF BLOOD FROZEN 6 GALLON CASE AMERICAN”). It is expected that fresh blood costs in The Gambia will be less than US prices due to lack of a market for it in The Gambia. (DOCX) [file pgph.0004903.s020.docx]

**S17 Table: Fresh blood estimate: utilizing locally sourced blood.** Pricing is based on blood obtained from food markets in the US(Supplier: B&R Food Service, Product: “BEEF BLOOD FROZEN 6 GALLON CASE AMERICAN”). It is expected that fresh blood costs in The Gambia will be less than US prices due to lack of a market for it in The Gambia.

| **Fecundity** | **Total Liters of Blood Per Year** | **Cost Per Liter (USD)** | **Total Cost (USD) Per Year** |
| --- | --- | --- | --- |
| **High** | 20.88 | <2.24 | <47 |
| **Low** | 25.2 | <2.24 | <56 |
