# Supplementary material for: Evaluating the cost of malaria elimination by Anopheles gambiae precision guided SIT in the Upper River region, The Gambia
Source: PLOS Glob Public Health. 2025 Jul 18;5(7):e0004903. doi: 10.1371/journal.pgph.0004903 (PMC12273942; doi:10.1371/journal.pgph.0004903)
Supplement: S18 Table — Hemotek device cost and annual fees. Cost data from Hemotek website (Accessed 2023). (DOCX) [file pgph.0004903.s021.docx]

#### S18 Table: Hemotek device cost and annual fees

Cost data from Hemotek website (Accessed 2023).

| **Requirements and Cost** | **High Fecundity** | **Low Fecundity** |
| --- | --- | --- |
| **Daily Cages** | 4 | 6 |
| **Daily Feeds** | 12 | 18 |
| **Hemoteks Required** | 2 | 3 |
| **Hemotek Cost** | 1,181 | 1,181 |
| **Total Hemotek Cost** | 2,362 | 3,543 |
| **Annual Repair Costs** | 118 | 177 |
| **Active Phase Feeds** | 144 | 216 |
| **Maintenance and Ramping Phase Feeds** | 204 | 204 |
| **Total Feeds** | 348 | 420 |
| **Membrane cost per feed** | 0.03 | 0.03 |
| **Annual Hemotek Costs** | 10.44 | 12.6 |
