# Supplementary material for: Evaluating the cost of malaria elimination by Anopheles gambiae precision guided SIT in the Upper River region, The Gambia
Source: PLOS Glob Public Health. 2025 Jul 18;5(7):e0004903. doi: 10.1371/journal.pgph.0004903 (PMC12273942; doi:10.1371/journal.pgph.0004903)
Supplement: S19 Table — Drone costs and annual fees. Cost data provided from a preliminary quote from Arda Impact. (DOCX) [file pgph.0004903.s022.docx]

#### S19 Table: Drone costs and annual fees

Cost data provided from a preliminary quote from Arda Impact.

| **Total Drones Used** | **Initial Drone Cost USD** | **Gallons of Gas per Flight** | **Annual Flight Numbers** | **Total Fuel Used Annually** | **Fuel Cost Annually USD** | **Maintenance Fees Annually USD** |
| --- | --- | --- | --- | --- | --- | --- |
| 4 | 100,000 | 0.75 | 196 | 147 | 657 | 10,000 |
