# Supplementary material for: Evaluating the cost of malaria elimination by Anopheles gambiae precision guided SIT in the Upper River region, The Gambia
Source: PLOS Glob Public Health. 2025 Jul 18;5(7):e0004903. doi: 10.1371/journal.pgph.0004903 (PMC12273942; doi:10.1371/journal.pgph.0004903)
Supplement: S20 Table — The costs of rearing larvae in The Upper River region. Cost data based on current costs of training and public outreach in The Gambia for current interventions, water and food costs described in Section 1.3.9a and Table S14, and Labor Costs are described in Section 1.2.13b and were considered to be sufficient to maintain part time managers. (DOCX) [file pgph.0004903.s023.docx]

#### S20 Table: The costs of rearing larvae in The Upper River region

Cost data based on current costs of training and public outreach in The Gambia for current interventions, water and food costs described in Section 1.3.9a and Table S14, and Labor Costs are described in Section 1.2.13b and were considered to be sufficient to maintain part time managers.

| **Upper River Region Rearing Costs** | **Training, Public Outreach and Delivery** | **Upper River Rearing Water Cost USD** | **Upper River Rearing Food Cost USD** | **Upper River Rearing Labor Cost USD** | **Total USD** |
| --- | --- | --- | --- | --- | --- |
| **Annual Cost** | 60,000 | 3,600 | 2,600 | 78,750 | 144,950 |
