# Supplementary material for: Evaluating the cost of malaria elimination by Anopheles gambiae precision guided SIT in the Upper River region, The Gambia
Source: PLOS Glob Public Health. 2025 Jul 18;5(7):e0004903. doi: 10.1371/journal.pgph.0004903 (PMC12273942; doi:10.1371/journal.pgph.0004903)
Supplement: S21 Table — Mutation rates and quality control. The background mutation rate is based on estimates from another member of the Anopheles gambiae species complex, Anopheles coluzzi [38]. To estimate the expected mutation rate per generation, we use estimates of the genetic element base lengths and the total number of mosquitoes per generation. These calculations can then be used to determine the mutation rate over time in the maintenance and active phase, and the expected mutation rate in the Cas9-gRNA pgSIT offspring. (DOCX) [file pgph.0004903.s024.docx]

#### S21 Table: Mutation rates and quality control:

The background mutation rate is based on estimates from another member of the *Anopheles gambiae* species complex, *Anopheles coluzzi* [[34]](https://paperpile.com/c/JoQtIv/qkzCN). To estimate the expected mutation rate per generation, we use estimates of the genetic element base lengths and the total number of mosquitoes per generation. These calculations can then be used to determine the mutation rate over time in the maintenance and active phase, and the expected mutation rate in the Cas9-gRNA pgSIT offspring.

| **Generation Specificity** | **Genetic Line** | **Background Mutation Rate per base per Generation** | **Transgene Bases Estimate** | **Mutation Rate per Mosquito per Generation** | **Mosquitoes per Generation** | **Mutations per Generation** |
| --- | --- | --- | --- | --- | --- | --- |
| **Active Phase- Released** | **Cas9- gRNA Progeny** | 1.00E-09 | 4,104 | 4.10E-6 | 8,686,000 | 35.65* |
| **Active Phase** | **Cas9 Line** | 1.00E-09 | 8,208 | 8.208E-6 | 87,697 | 0.72 |
| **Active Phase** | **gRNA Line** | 1.00E-09 | 2,400 | 2.58E-06 | 87,697 | 0.23^#^ |
| **Maintenance Phase** | **Cas9 Line** | 1.00E-09 | 8,208 | 8.208E-6 | 7,500 | 0.06 |
| **Maintenance Phase** | **gRNA Line** | 1.00E-09 | 2,400 | 2.58E-06 | 7,500 | 0.02^#^ |

***** gRNA-Cas9 progeny are not returned to the colony and if pgSIT function is maintained, should be primarily sterile males, so these mutations are lost to the next generation.

^#^ This is the chance of a single mutation in the gRNA line. If considering inactivation of these lines, the gRNA lines would require a minimum of 3 mutations across each of the gRNAs for one of the gene targets. This means that the Active Phase would have less than 0.012 chance per

generation and the Maintenance Phase would have less than 0.000008 chance per generation, requiring approximately 125,000 generations for a set of deactivating mutations to occur that would deactivate the gRNA genes. For this facility, producing mosquitoes at these amounts, we would require about 6,250 years before we would expect to see one set of these mutations. These chances are an overestimate as they do not account for the shrinking target as this would have to mutate unmutated gRNAs to have an additional effect. This also does not account for some percentage of mutations not having a deactivating effect on all of the gRNAs if they occur in specific parts of the gRNA.
