# Supplementary material for: Evaluating the cost of malaria elimination by Anopheles gambiae precision guided SIT in the Upper River region, The Gambia
Source: PLOS Glob Public Health. 2025 Jul 18;5(7):e0004903. doi: 10.1371/journal.pgph.0004903 (PMC12273942; doi:10.1371/journal.pgph.0004903)
Supplement: S23 Table — Banjul Land Cost Estimate. Survey of available property on AccessGambia near Banjul with costs converted to USD to determine average cost per square meter. (DOCX) [file pgph.0004903.s026.docx]

#### S23 Table: Banjul Land Cost Estimate

Survey of available property on AccessGambia near Banjul with costs converted to USD to determine average cost per square meter.

|  | **Property 1** | **Property 2** | **Property 3** | **Property 4** | **Property 5** | **Property 6** | **Property 7** | **Average** |
| --- | --- | --- | --- | --- | --- | --- | --- | --- |
| **Price (USD)** | 7,000 | 2,112 | 20,207 | 15,155 | 4,929 | 7,042 | 4,225 |  |
| **Area (Square Meter)** | 500 | 960 | 14,700 | 1,500 | 900 | 500 | 400 |  |
| **Price per square meter** | 14 | 2.2 | 1.37 | 10.1 | 5.48 | 14.08 | 10.56 | 8.25 |
