# Supplementary material for: Evaluating the cost of malaria elimination by Anopheles gambiae precision guided SIT in the Upper River region, The Gambia
Source: PLOS Glob Public Health. 2025 Jul 18;5(7):e0004903. doi: 10.1371/journal.pgph.0004903 (PMC12273942; doi:10.1371/journal.pgph.0004903)
Supplement: S24 Table — Minimum facility size, land, and cost. Land requirements were estimated as described in Section 1.2.16a. Cost data was estimated by averaging cost per square meter of land based on available plots of land on The Gambian government’s website near Banjul. (DOCX) [file pgph.0004903.s027.docx]

#### S24 Table: Minimum facility size, land, and cost

Land requirements were estimated as described in Section 1.2.16a. Cost data was estimated by averaging cost per square meter of land based on available plots of land on The Gambian government’s website near Banjul.

| **Conditions** | **Racks Required** | **m^2 of Space for Racks** | **Cages Required** | **m^2 of Space for Cages** | **Facility Size** | **Land Required m^2** | **Facility Cost (USD)** | **Land Cost (USD)** | **Architect Fee Estimate USD** | **Total (USD)** | **Total Cost with 10% Contingency Cost (USD)** | **Maintenance Fee (5% RAV)** |
| --- | --- | --- | --- | --- | --- | --- | --- | --- | --- | --- | --- | --- |
| **COPAS Sorting, High Fecundity, High Survival** | 2 | 4 | 4 | 16 | 20 | 40 | 32,000 | 350 | 4,500 | 36,850 | 40,535 | 2,027 |
| **COPAS Sorting, Low Fecundity, High Survival** | 2 | 4 | 4 | 16 | 20 | 40 | 32,000 | 350 | 4,500 | 36,850 | 40,535 | 2,027 |
| **COPAS Sorting, High Fecundity, Low Survival** | 3 | 9 | 6 | 36 | 45 | 90 | 72,000 | 780 | 10,080 | 82,860 | 91,146 | 4,557 |
| **COPAS Sorting, Low Fecundity, Low Survival** | 3 | 9 | 6 | 36 | 45 | 90 | 72,000 | 780 | 10,080 | 82,860 | 91,146 | 4,557 |
