# Supplementary material for: Evaluating the cost of malaria elimination by Anopheles gambiae precision guided SIT in the Upper River region, The Gambia
Source: PLOS Glob Public Health. 2025 Jul 18;5(7):e0004903. doi: 10.1371/journal.pgph.0004903 (PMC12273942; doi:10.1371/journal.pgph.0004903)
Supplement: S25 Table — High wage annual estimate. GMD- Gambian dalasi. Wages estimated via communication with Umberto D’Alesandro and PayLab survey information retrieved in 2023. High, average and low estimates from these surveys were utilized below in Tables S25-S27 respectively. (DOCX) [file pgph.0004903.s028.docx]

#### S25 Table: High wage annual estimate

GMD- Gambian dalasi. Wages estimated via communication with Umberto D’Alesandro and PayLab survey information retrieved in 2023. High, average and low estimates from these surveys were utilized below in Tables S25-S27 respectively.

| **Personnel** | **GMD per year** | **USD per year** | **USD per Month** | **GMD/month** | **Number of Employees** | **Total Annual Cost (USD)** |
| --- | --- | --- | --- | --- | --- | --- |
| **Engineering Manufacturing Manager/Repair Technician** | 487,200 | 7,795 | 650 | 40,600 | 2 | 15,590 |
| **Logistics Manager** | 336,000 | 5,376 | 448 | 28,000 | 2 | 10,752 |
| **Lead factory manager:** | 487,200 | 7,795 | 650 | 40,600 | 1 | 7,795 |
| **Supervisor** | 167,000 | 2,672 | 223 | 13,917 | 2 | 5,344 |
| **Egg Harvesting laborer** | 115,000 | 1,840 | 153 | 9,583 | 1 | 1,840 |
| **Egg Water Preparer** | 115,000 | 1,840 | 153 | 9,583 | 1 | 1,840 |
| **Blood Feeding** | 115,000 | 1,840 | 153 | 9,583 | 2 | 1,840 |
| **Mosquito Cage Cleaning** | 115,000 | 1,840 | 153 | 9,583 | 2 | 3,680 |
| **Cage Set Up** | 115,000 | 1,840 | 153 | 9,583 | 1 | 1,840 |
| **Pupae separation** | 115,000 | 1,840 | 153 | 9,583 | 1 | 1,840 |
| **Cage Feeding** | 115,000 | 1,840 | 153 | 9,583 | 1 | 1,840 |
| **New Rack Set Up** | 115,000 | 1,840 | 153 | 9,583 | 1 | 1,840 |
| **Engineering Manufacturing Manager/Repair Technician** | 487,200 | 7,795 | 650 | 40,600 | 2 | 15,590 |
| **Logistics Manager** | 336,000 | 5,376 | 448 | 28,000 | 2 | 10,752 |
| **Lead factory manager:** | 487,200 | 7,795 | 650 | 40,600 | 1 | 7,795 |
| **Supervisor** | 167,000 | 2,672 | 223 | 13,917 | 2 | 5,344 |
| **Egg Harvesting laborer** | 115,000 | 1,840 | 153 | 9,583 | 1 | 1,840 |
| **Egg Water Preparer** | 115,000 | 1,840 | 153 | 9,583 | 1 | 1,840 |
| **Blood Feeding** | 115,000 | 1,840 | 153 | 9,583 | 2 | 1,840 |
| **COPAS Sorting Technician** | 115,000 | 1,840 | 153 | 9,583 | 3 | 5,520 |
| **Total** |  |  |  |  | 20 | 61,561 |
