# Supplementary material for: Evaluating the cost of malaria elimination by Anopheles gambiae precision guided SIT in the Upper River region, The Gambia
Source: PLOS Glob Public Health. 2025 Jul 18;5(7):e0004903. doi: 10.1371/journal.pgph.0004903 (PMC12273942; doi:10.1371/journal.pgph.0004903)
Supplement: S26 Table — Medium wage annual estimate. (DOCX) [file pgph.0004903.s029.docx]

#### S26 Table: Medium wage annual estimate

| **Personnel** | **GMD per year** | **USD per year** | **USD per Month** | **Gambia Dalise/month** | **Number of Employees** | **Total Annual Cost (USD)** |
| --- | --- | --- | --- | --- | --- | --- |

| **Engineering Manufacturing Manager/Repair Technician** | 286,000 | 4,576 | 381 | 23,833 | 2 | 9,152 |
| --- | --- | --- | --- | --- | --- | --- |
| **Logistics Manager** | 214,000 | 3,424 | 285 | 17,833 | 2 | 6,848 |
| **Lead factory manager:** | 286,000 | 4,576 | 381 | 23,833 | 1 | 4,576 |
| **Supervisor** | 83,500 | 1,336 | 111 | 6,958 | 2 | 2,672 |
| **Egg Harvesting laborer** | 57,300 | 917 | 76 | 4,775 | 1 | 917 |
| **Egg Water Preparer** | 57,300 | 917 | 76 | 4,775 | 1 | 917 |
| **Blood Feeding** | 57,300 | 917 | 76 | 4,775 | 2 | 1,834 |
| **Mosquito Cage Cleaning** | 57,300 | 917 | 76 | 4,775 | 2 | 1,834 |
| **Cage Set Up** | 57,300 | 917 | 76 | 4,775 | 1 | 917 |
| **Pupae separation** | 57,300 | 917 | 76 | 4,775 | 1 | 917 |
| **Cage Feeding** | 57,300 | 917 | 76 | 4,775 | 1 | 917 |
| **New Rack Set Up** | 57,300 | 917 | 76 | 4,775 | 1 | 9172 |
| **COPAS Sorting Technician** | 115,000 | 1,840 | 153 | 9,583 | 3 | 5,520 |
| **Total** | **1,442,900** | **23,086** | **1,924** | **120,242** | **20** | **37,938** |
