# Supplementary material for: Evaluating the cost of malaria elimination by Anopheles gambiae precision guided SIT in the Upper River region, The Gambia
Source: PLOS Glob Public Health. 2025 Jul 18;5(7):e0004903. doi: 10.1371/journal.pgph.0004903 (PMC12273942; doi:10.1371/journal.pgph.0004903)
Supplement: S27 Table — Low wage annual estimate. (DOCX) [file pgph.0004903.s030.docx]

#### S27 Table: Low wage annual estimate

| **Personnel** | **GMD per year** | **USD per year** | **USD per Month** | **Gambia Dalise/month** | **Number of Employees** | **Total Annual Cost (USD)** |
| --- | --- | --- | --- | --- | --- | --- |
| **Engineering Manufacturing Manager/Repair Technician** | 214,500 | 3,432 | 286 | 17,875 | 2 | 6,864 |
| **Logistics Manager** | 160,500 | 2,568 | 214 | 13,375 | 2 | 5,136 |
| **Lead factory manager:** | 214,500 | 3,432 | 286 | 17,875 | 1 | 3,432 |
| **Supervisor** | 62,625 | 1,002 | 84 | 5,219 | 2 | 2,004 |
| **Egg Harvesting laborer** | 42,975 | 688 | 57 | 3,581 | 1 | 688 |
| **Egg Water Preparer** | 42,975 | 688 | 57 | 3,581 | 1 | 688 |
| **Blood Feeding** | 42,975 | 688 | 57 | 3,581 | 2 | 1,376 |
| **Mosquito Cage Cleaning** | 42,975 | 688 | 57 | 3,581 | 2 | 1,376 |
| **Cage Set Up** | 42,975 | 688 | 57 | 3,581 | 1 | 688 |

| **Pupae separation** | 42,975 | 688 | 57 | 3,581 | 1 | 688 |
| --- | --- | --- | --- | --- | --- | --- |
| **Cage Feeding** | 42,975 | 688 | 57 | 3,581 | 1 | 688 |
| **New Rack Set Up** | 42,975 | 688 | 57 | 3,581 | 1 | 688 |
| **COPAS Sorting Technician** | 86,250 | 1,380 | 115 | 7,188 | 3 | 4,140 |
| **Total** | 1,082,175 | 17,315 | 1,443 | 90,181 | 20 | 28,456 |
