# Supplementary material for: Evaluating the cost of malaria elimination by Anopheles gambiae precision guided SIT in the Upper River region, The Gambia
Source: PLOS Glob Public Health. 2025 Jul 18;5(7):e0004903. doi: 10.1371/journal.pgph.0004903 (PMC12273942; doi:10.1371/journal.pgph.0004903)
Supplement: S28 Table — Total initial costs with least expensive trials. This cost assessment is based on the direct cost estimates of trials based on assumed reagent requirements, wages for local employees in The Gambia and other expected expenses. The Upper River Rearing Costs assumes the need to prepare these sites with a trial run. A high cost can be seen in Table S29. (DOCX) [file pgph.0004903.s031.docx]

#### S28 Table: Total initial costs with least expensive trials:

This cost assessment is based on the direct cost estimates of trials based on assumed reagent requirements, wages for local employees in The Gambia and other expected expenses. The Upper River Rearing Costs assumes the need to prepare these sites with a trial run. A high cost can be seen in Table 29.

| **Conditions** | **Rack Cost** | **Cage Cost** | **Facility Cost** | **Hemotek Cost** | **Sorting Machine** | **Tech Dev. Costs** | **Drone, Infra. & Refrig. Cost** | **Upper River Rearing Sites** | **Initial Monitoring Costs** | **Initial Training** | **Total** |
| --- | --- | --- | --- | --- | --- | --- | --- | --- | --- | --- | --- |
| **COPAS**  **Sorting, High Fecundity, High Survival** | 45,000 | 15,000 | 40,535 | 2,362 | 948,840 | 2,801,655 | 140,000 | 144,950 | 752,500 | 936,000 | 5,826,842 |
| **COPAS**  **Sorting, Low Fecundity, High Survival** | 45,000 | 22,500 | 40,535 | 3,543 | 948,840 | 2,801,655 | 140,000 | 144,950 | 752,500 | 936,000 | 5,835,523 |
| **COPAS Sorting, High Fecundity, Low Survival** | 67,500 | 15,000 | 91,146 | 2,362 | 948,840 | 2,801,655 | 140,000 | 144,950 | 752,500 | 936,000 | 5,899,953 |
| **COPAS Sorting, Low Fecundity, Low Survival** | 67,500 | 22,500 | 91,146 | 3,543 | 948,840 | 2,801,655 | 140,000 | 144,950 | 752,500 | 936,000 | 5,908,634 |
