# Supplementary material for: Evaluating the cost of malaria elimination by Anopheles gambiae precision guided SIT in the Upper River region, The Gambia
Source: PLOS Glob Public Health. 2025 Jul 18;5(7):e0004903. doi: 10.1371/journal.pgph.0004903 (PMC12273942; doi:10.1371/journal.pgph.0004903)
Supplement: S29 Table — Total initial costs with more expensive trials. This table includes additional costs associated with the larger and more expensive field trials and additional machines for sex sorting. The additional field trial cost are: 1) 500,000 USD per year for the five year monitoring period, 2) an additional 2.5 million USD for the larger scale cluster randomized trial (approximate cost of a previous large scale trial [6] as per communication with Umberto D’Alessandro) and 3) 500,000 USD to pay for a social science team to manage public communication of pgSIT technology. As a facility of this scale has not been developed, and extensive testing has not yet been conducted with these machines, this may need to be tested. These estimates should represent the upper limit of initial costs, but there are significant uncertainties on whether these expenditures will be necessary. (DOCX) [file pgph.0004903.s032.docx]

#### S29 Table: Total initial costs with more expensive trials

This table includes additional costs associated with the larger and more expensive field trials and additional machines for sex sorting. The additional field trial cost are: 1) 500,000 USD per year for the five year monitoring period, 2) an additional 2.5 million USD for the larger scale cluster randomized trial (approximate cost of a previous large scale trial[[6]](https://paperpile.com/c/JoQtIv/rcYb) as per communication with Umberto D’Alessandro) and 3) 500,000 USD to pay for a social science team to manage public communication of pgSIT technology. As a facility of this scale has not been developed, and extensive testing has not yet been conducted with these machines, this may need to be tested. These estimates should represent the upper limit of initial costs, but there are significant uncertainties on whether these expenditures will be necessary.

| **Initial Cost** | **Rack Cost** | **Cage Cost** | **Facility Cost** | **Hemotek Cost** | **Sorting Machine** | **Development Costs** | **Drone, Generator & Refrigerato r Cost** | **Upper River Rearing Sites** | **Initial Monitorin g Costs** | **Initial Training** | **Total** |
| --- | --- | --- | --- | --- | --- | --- | --- | --- | --- | --- | --- |
| **COPAS**  **Sorting, High Fecundity, High Survival** | 76,000 | 150,000 | 40,535 | 2,362 | 948,840 | 5,801,655 | 140,000 | 144,950 | 3,252,500 | 936,000 | 11,492,842 |
| **COPAS**  **Sorting, High Fecundity, High Survival** | 76,000 | 150,000 | 40,535 | 2,362 | 948,840 | 5,801,655 | 140,000 | 144,950 | 3,252,500 | 936,000 | 11,492,842 |
| **COPAS**  **Sorting, Low Fecundity, High Survival** | 76,000 | 225,000 | 40,535 | 3,543 | 948,840 | 5,801,655 | 140,000 | 144,950 | 3,252,500 | 936,000 | 11,569,023 |
| **COPAS**  **Sorting, High Fecundity, Low Survival** | 114,000 | 150,000 | 91,146 | 2,362 | 948,840 | 5,801,655 | 140,000 | 144,950 | 3,252,500 | 936,000 | 11,581,453 |
| **COPAS**  **Sorting, Low Fecundity, Low Survival** | 114,000 | 225,000 | 91,146 | 3,543 | 948,840 | 5,801,655 | 140,000 | 144,950 | 3,252,500 | 936,000 | 11,657,634 |
