# Supplementary material for: Evaluating the cost of malaria elimination by Anopheles gambiae precision guided SIT in the Upper River region, The Gambia
Source: PLOS Glob Public Health. 2025 Jul 18;5(7):e0004903. doi: 10.1371/journal.pgph.0004903 (PMC12273942; doi:10.1371/journal.pgph.0004903)
Supplement: S30 Table — Total annual costs. The total annual cost includes facility costs, including maintenance fees for the equipment and the high estimated cost in The Gambia, for labor, local resources and rearing in the URR. Rearing costs may be overestimated, however, as other malaria interventions rely on some volunteer labor. Maintenance of larvae at sites in the URR may also utilize other more affordable, local resources rather than imported mosquito feed. (DOCX) [file pgph.0004903.s033.docx]

#### S30 Table: Total annual costs:

The total annual cost includes facility costs, including maintenance fees for the equipment and the high estimated cost in The Gambia, for labor, local resources and rearing in the URR. Rearing costs may be overestimated, however, as other malaria interventions rely on some volunteer labor. Maintenance of larvae at sites in the URR may also utilize other more affordable, local resources rather than imported mosquito feed.

| **Conditions** | **Maintenance Fees*** | **Labor Costs** | **Water Cost** | **Larval Feed Costs** | **Blood Feed Cost** | **Upper River Rearing Site Cost** | **Total** |
| --- | --- | --- | --- | --- | --- | --- | --- |
| **High Estimate Wages/Maintenance, COPAS Sorting: High Fecundity, High Survival** | 113,906 | 61,561 | 371 | 222 | 47 | 144,950 | 315,168 |
| **High Estimate Wages/Maintenance, COPAS Sorting: Low Fecundity, High Survival** | 117,656 | 61,561 | 371 | 222 | 56 | 144,950 | 321,057 |
| **High Estimate Wages/Maintenance, COPAS Sorting: High Fecundity, Low Survival** | 114,286 | 61,561 | 466 | 268 | 47 | 144,950 | 324,816 |
| **High Estimate Wages/Maintenance, COPAS Sorting: Low Fecundity, Low Survival** | 118,036 | 61,561 | 466 | 268 | 56 | 144,950 | 321,578 |
| **High Estimate Wages/Maintenance, COPAS Sorting: High Fecundity, High Survival, No URR Cost** | 113,906 | 61,561 | 371 | 222 | 47 | 0 | 325,337 |
| **High Estimate Wages/Maintenance, COPAS Sorting: Low Fecundity, High Survival, No URR Cost** | 117,656 | 61,561 | 371 | 222 | 56 | 0 | 176,107 |
| **High Estimate Wages/Maintenance, COPAS Sorting: High Fecundity, Low Survival, No URR**  **Cost** | 114,286 | 61,561 | 466 | 268 | 47 | 0 | 179,866 |
| **High Estimate Wages/Maintenance, COPAS Sorting: Low Fecundity, Low Survival, No URR Cost** | 118,036 | 61,561 | 466 | 268 | 56 | 0 | 180,387 |
| **COPAS Sorting, High**  **Fecundity, High Survival** | 109,111 | 37,938 | 371 | 222 | 47 | 144,950 | 292,639 |
| **COPAS**  **Sorting, Low Fecundity, High Survival** | 111,174 | 37,938 | 371 | 222 | 56 | 144,950 | 295,711 |
| **COPAS**  **Sorting, High Fecundity, Low Survival** | 109,414 | 37,938 | 466 | 268 | 47 | 144,950 | 293,083 |
| **COPAS**  **Sorting, Low Fecundity, Low Survival** | 111,476 | 37,938 | 466 | 268 | 56 | 144,950 | 295,154 |
| **COPAS Sorting, High**  **Fecundity, High Survival, No URR Cost** | 109,111 | 37,938 | 371 | 222 | 47 | 0 | 147,689 |
| **COPAS**  **Sorting, Low Fecundity, High Survival, No URR Cost** | 111,174 | 37,938 | 371 | 222 | 56 | 0 | 149,761 |
| **COPAS**  **Sorting, High Fecundity, Low Survival, No URR Cost** | 109,414 | 37,938 | 466 | 268 | 47 | 0 | 148,133 |
| **COPAS**  **Sorting, Low Fecundity, Low Survival, No URR Cost** | 111,476 | 37,938 | 466 | 268 | 56 | 0 | 150,204 |
| **COPAS Sorting, High**  **Fecundity, High Survival** | 104,316 | 28,456 | 371 | 222 | 47 | 144,950 | 278,362 |
| **COPAS**  **Sorting, Low Fecundity, High Survival** | 104,691 | 28,456 | 371 | 222 | 56 | 144,950 | 278,746 |
| **COPAS**  **Sorting, High Fecundity, Low Survival** | 104,541 | 28,456 | 466 | 268 | 47 | 144,950 | 278,728 |
| **COPAS**  **Sorting, Low Fecundity, Low Survival** | 104,916 | 28,456 | 466 | 268 | 56 | 144,950 | 279,112 |
| **COPAS Sorting, High**  **Fecundity, High Survival, No URR Cost** | 104,316 | 28,456 | 371 | 222 | 47 | 0 | 133,412 |
| **COPAS**  **Sorting, Low Fecundity, High Survival, No URR Cost** | 104,691 | 28,456 | 371 | 222 | 56 | 0 | 133,796 |
| **COPAS**  **Sorting, High Fecundity, Low Survival, No URR Cost** | 104,541 | 28,456 | 466 | 268 | 47 | 0 | 133,778 |
| **COPAS**  **Sorting, Low Fecundity, Low Survival, No URR Cost** | 104,916 | 28,456 | 466 | 268 | 56 | 0 | 134,162 |
