# Supplementary material for: Evaluating the cost of malaria elimination by Anopheles gambiae precision guided SIT in the Upper River region, The Gambia
Source: PLOS Glob Public Health. 2025 Jul 18;5(7):e0004903. doi: 10.1371/journal.pgph.0004903 (PMC12273942; doi:10.1371/journal.pgph.0004903)
Supplement: S31 Table — Estimated age stratified life years saved. (DOCX) [file pgph.0004903.s034.docx]

#### S31 Table: Estimated age stratified life years saved

| **Age Bracket** | **0 to 5 Years** | **5 to 17 Years** | **17 to 40 Years** | **40 to 60 Years** | **60 Years and Older** |
| --- | --- | --- | --- | --- | --- |
| **Average Age** | 2.5 | 11 | 28.5 | 50 | N/A |
| **Years Saved** | 60.1 | 51.6 | 34.1 | 12.6 | 1 |
