# Supplementary material for: Evaluating the cost of malaria elimination by Anopheles gambiae precision guided SIT in the Upper River region, The Gambia
Source: PLOS Glob Public Health. 2025 Jul 18;5(7):e0004903. doi: 10.1371/journal.pgph.0004903 (PMC12273942; doi:10.1371/journal.pgph.0004903)
Supplement: S32 Table — Life years saved annually. (DOCX) [file pgph.0004903.s035.docx]

#### S32 Table: Life years saved annually

| **Intervention Year** | **0-5 years** | **5-17 years** | **17-40 years** | **40-60 years** | **≥60 years** | **Total** |
| --- | --- | --- | --- | --- | --- | --- |
| **0*** | 3 | 6 | 1 | 0 | 0 | -5 |
| **1** | -2 | -5 | 0 | 0 | 0 | 133 |
| **2**# | 2,855 | 5,897 | 556 | 43 | 2 | 9,353 |
| **3** | 3,446 | 7,2790 | 689 | 53 | 3 | 11,461 |
| **4** | 3,433 | 7,389 | 705 | 54 | 3 | 11,584 |
| **5** | 3,443 | 7,538 | 725 | 55 | 3 | 11,763 |

* Year 0 has no intervention.

^#^ Year 2 the intervention begins part way through the year and this model assumes an accumulative suppressive effect, year to year.
