# Supplementary material for: Evaluating the cost of malaria elimination by Anopheles gambiae precision guided SIT in the Upper River region, The Gambia
Source: PLOS Glob Public Health. 2025 Jul 18;5(7):e0004903. doi: 10.1371/journal.pgph.0004903 (PMC12273942; doi:10.1371/journal.pgph.0004903)
Supplement: S33 Table — Sick days prevented by age over four years of pgSIT interventions. (DOCX) [file pgph.0004903.s036.docx]

| **Intervention Year** | **0-5 years** | **5-17 years** | **17-40 years** | **40-60 years** | **≥60 years** | **Annual Total** |
| --- | --- | --- | --- | --- | --- | --- |
| **0*** | 2 | 17 | 21 | 4 | 3 | 47 |
| **1** | -1 | -11 | -14 | -3 | -2 | -31 |
| **2#** | 1,673 | 13,974 | 18,045 | 3,599 | 2,198 | 39,488 |
| **3** | 2,020 | 16,897 | 21,887 | 4,374 | 2,673 | 47,851 |
| **4** | 2,012 | 16,835 | 21,844 | 4,374 | 2,678 | 47,743 |
| **5** | 2,016 | 16,879 | 21,934 | 4,400 | 2,698 | 47,928 |

#### S33 Table: Sick days prevented by age over four years of pgSIT interventions

* Year 0 has no intervention.

^#^ Year 2 the intervention begins part way through the year and this model assumes an accumulative suppressive effect, year to year.
