# Supplementary material for: Evaluating the cost of malaria elimination by Anopheles gambiae precision guided SIT in the Upper River region, The Gambia
Source: PLOS Glob Public Health. 2025 Jul 18;5(7):e0004903. doi: 10.1371/journal.pgph.0004903 (PMC12273942; doi:10.1371/journal.pgph.0004903)
Supplement: S34 Table — Value of statistical life calculations. (DOCX) [file pgph.0004903.s037.docx]

#### S34 Table: Value of statistical life calculations

| **Intervention Year** | **1.0 Elasticity** | | | | | | **1.5 Elasticity** | **2.0 Elasticity** |
| --- | --- | --- | --- | --- | --- | --- | --- | --- |
|  | **0-5 years (USD)** | **5-17 years (USD)** | **17-40 years (USD)** | **40-60 years (USD)** | **≥60 years (USD)** | **Total USD** | **Total USD** | **Total USD** |
| **2** | 18,725,706 | 45,040,845 | 6,425,994 | 1,338,332 | 895,350 | 72,426,227 | 12,877,608 | 2,286,752 |
| **3** | 22,596,422 | 55,531,736 | 7,966,466 | 1,650,749 | 1,097,969 | 88,843,364 | 15,747,075 | 2,796,300 |
| **4** | 22,515,580 | 56437,526 | 8,153,552 | 1,682,077 | 1,112,464 | 89,901,200 | 15,957,036 | 2,833,584 |
| **5** | 22,576,631 | 57,581,558 | 8,384,564 | 1,723,547 | 1,133,911 | 91,400,211 | 16,236,984 | 2,883,296 |
