# Supplementary material for: Evaluating the cost of malaria elimination by Anopheles gambiae precision guided SIT in the Upper River region, The Gambia
Source: PLOS Glob Public Health. 2025 Jul 18;5(7):e0004903. doi: 10.1371/journal.pgph.0004903 (PMC12273942; doi:10.1371/journal.pgph.0004903)
Supplement: S35 Table — Quality adjusted life year calculations. (DOCX) [file pgph.0004903.s038.docx]

| **Intervention Year** | **0 to 5 Years USD** | **5 to 17 Years USD** | **17 to 40 Years USD** | **40 to 60 Years USD** | **60 Years and Older USD** | **Total USD by QALY Saved Per Year** |
| --- | --- | --- | --- | --- | --- | --- |
| **2** | 9,365,633 | 19,341,077 | 1,823,556 | 140,333 | 7,451 | 30,678,050 |
| **3** | 11,301,565 | 23,845,992 | 2,260,714 | 173,092 | 9,137 | 37,590,500 |
| **4** | 11,261,132 | 24,234,948 | 2,313,799 | 176,377 | 9,258 | 37,995,514 |
| **5** | 11,291,666 | 24,726,210 | 2,379,355 | 180,725 | 9,436 | 38,587,393 |

#### S35 Table: Quality adjusted life year calculations
