# Supplementary material for: Evaluating the cost of malaria elimination by Anopheles gambiae precision guided SIT in the Upper River region, The Gambia
Source: PLOS Glob Public Health. 2025 Jul 18;5(7):e0004903. doi: 10.1371/journal.pgph.0004903 (PMC12273942; doi:10.1371/journal.pgph.0004903)
Supplement: S36 Table — GDP growth estimate. (DOCX) [file pgph.0004903.s039.docx]

#### S36 Table: GDP growth estimate

| **Year** | **GDP without Intervention (USD)** | **GDP with Intervention (USD)** | **Annual Effect of Intervention (USD)** |
| --- | --- | --- | --- |
| **2021 GDP of The Gambia** | 2,038,000,000 | 2,038,000,000 | Pre-intervention |
| **Predicted 2022** | 2,110,349,000 | 2,110,349,000 | Pre-intervention |
| **Predicted 2023** | 2,185,266,390 | 2,185,266,390 | Pre-intervention |
| **Predicted 2024** | 2,262,843,346 | 2,262,843,346 | Pre-intervention |
| **Predicted 2025** | 2,343,174,285 | 2,343,174,285 | Pre-intervention |
| **Predicted 2026** | 2,426,356,972 | 2,426,356,972 | Pre-intervention |
| **Predicted 2027** | 2,512,492,645 | 2,512,492,645 | Pre-intervention |
| **Predicted 2028** | 2,601,686,134 | 2,601,686,134 | Pre-intervention |
| **Predicted 2029** | 2,694,045,991 | 2,694,045,991 | Pre-intervention |
| **Predicted 2030** | 2,789,684,624 | 2,797,766,762 | 8,082,138 |
| **Predicted 2031** | 2,888,718,428 | 2,905,480,782 | 16,762,354 |
| **Predicted 2032** | 2,991,267,932 | 3,017,341,793 | 26,073,860 |
| **Predicted 2033** | 3,097,457,944 | 3,133,509,452 | 36,051,507 |
| **Predicted 2034** | 3,207,417,701 | 3,254,149,565 | 46,731,864 |
| **Predicted 2035** | 3,321,281,029 | 3,379,434,324 | 58,153,294 |
| **Predicted 2036** | 3,439,186,506 | 3,509,542,545 | 70,356,039 |
| **Predicted 2037** | 3,561,277,627 | 3,644,659,933 | 83,382,306 |
| **Predicted 2038** | 3,687,702,983 | 3,784,979,341 | 97,276,358 |
| **Predicted 2039** | 3,818,616,439 | 3,930,701,045 | 112,084,607 |
| **Predicted 2040** | 3,918,057,685 | 4,044,807,585 | 126,749,900 |
| **Predicted 2041** | 4,055,189,704 | 4,198,510,273 | 143,320,569 |
| **Predicted 2042** | 4,197,121,343 | 4,358,053,663 | 160,932,320 |
| **Predicted 2043** | 4,344,020,590 | 4,523,659,703 | 179,639,112 |
| **Predicted 2044** | 4,496,061,311 | 4,695,558,771 | 199,497,460 |
| **Predicted 2045** | 4,653,423,457 | 4,873,990,005 | 220,566,548 |
