# Supplementary material for: Evaluating the cost of malaria elimination by Anopheles gambiae precision guided SIT in the Upper River region, The Gambia
Source: PLOS Glob Public Health. 2025 Jul 18;5(7):e0004903. doi: 10.1371/journal.pgph.0004903 (PMC12273942; doi:10.1371/journal.pgph.0004903)
Supplement: S37 Table — Value of sick days saved. (DOCX) [file pgph.0004903.s040.docx]

#### S37 Table: Value of sick days saved

| **Intervention Year** | **0-5 years** | **5-17 years** | **17-40 years** | **40-60 years** | **≥60 years** | **Total** |
| --- | --- | --- | --- | --- | --- | --- |
| **2** | 21,688 | 181,100 | 233,858 | 46,644 | 28,480 | 511,770 |
| **3** | 26,174 | 218,991 | 283,652 | 56,684 | 34,646 | 620,147 |
| **4** | 26,070 | 218,187 | 283,101 | 56,684 | 34,703 | 618,745 |
| **5** | 26,133 | 218,749 | 284,267 | 57,025 | 34,971 | 621,144 |
