# Supplementary material for: Evaluating the cost of malaria elimination by Anopheles gambiae precision guided SIT in the Upper River region, The Gambia
Source: PLOS Glob Public Health. 2025 Jul 18;5(7):e0004903. doi: 10.1371/journal.pgph.0004903 (PMC12273942; doi:10.1371/journal.pgph.0004903)
Supplement: S38 Table — Annualized mean costs of the interventions against malaria in the Upper River Region (in 2021 USD). (DOCX) [file pgph.0004903.s041.docx]

#### S38 Table: Annualized mean costs of the interventions against malaria in the Upper River Region (in 2021 USD)

| **Annualized mean costs of the interventions against malaria in the Upper River Region, in 2022 USD** |  | | | | |  |
| --- | --- | --- | --- | --- | --- | --- |
| **Intervention Category** | **SMC** | **LLINs** | **IRS** | **IPTp** | **Case management** | **Total** |
| Medicines & supplies | 3,640 | 0 | 0 | 0 | 24,283 | 27,923 |
| Insecticides & insecticide treated nets | 0 | 180,423 | 24,890 | 0 | 0 | 205,313 |
| Distribution remuneration | 12,223 | 31,841 | 20,267 | 0 | 0 | 64,331 |
| Management | 4,555 | 24,302 | 0 | 0 | 0 | 28,857 |
| Supervision | 4,139 | 6,510 | 3,804 | 0 | 48 | 14,500 |
| Meetings | 0 | 2,386 | 0 | 121 | 0 | 2,507 |
| Trainings | 2,230 | 2,193 | 1,968 | 348 | 1,290 | 8,029 |
| Social mobilization | 222 | 0 | 1,453 | 0 | 0 | 1,676 |
| Start-up costs (phones, solar systems) | 0 | 9,020 | 2,053 | 239 | 0 | 11,312 |
| Other (maintenance, etc) | 0 | 2,722 | 2,255 | 0 | 3,298 | 8,275 |
| **Total** | **27,009** | **259,397** | **56,690** | **708** | **28,919** | **372,723** |

URR Seasonal Malaria Chemoprevention (SMC) costs : based on total number of children under five in the URR in proportion of total population of under five living in The Gambia

URR LLIN, IRS & case management costs : based on total number of population living in the URR in proportion of total population of The Gambia

URR Intermittent preventive treatment in pregnant women (IPTp) costs : based on total female population in the URR in proportion of total female population living in The Gambia
