# Supplementary material for: Evaluating the cost of malaria elimination by Anopheles gambiae precision guided SIT in the Upper River region, The Gambia
Source: PLOS Glob Public Health. 2025 Jul 18;5(7):e0004903. doi: 10.1371/journal.pgph.0004903 (PMC12273942; doi:10.1371/journal.pgph.0004903)
Supplement: S39 Table — Costs associated with malaria treatment seeking. Adapted from [55] with conversions from British pound (GBP) to USD. (DOCX) [file pgph.0004903.s042.docx]

#### S39 Table: Costs associated with malaria treatment seeking

Adapted from[[51]](https://paperpile.com/c/JoQtIv/sivAB) with conversions from British pound (GBP) to USD.

| **Years** | **Complicated Cases** | **Uncomplicated Cases** | **Household Costs for seeking treatment GBP** | **Household Costs for seeking treatment USD** | **Average Per Household per Case USD** | **Average GBP to USD by Year** |
| --- | --- | --- | --- | --- | --- | --- |
| **2013** | 1,515 | 45,479 | 145,021 | 226,233 | 4.81 | 1.56 |
| **2014** | 933 | 26,806 | 85,613 | 141,261 | 5.09 | 1.65 |
| **2015** | 1,273 | 52,007 | 164,288 | 251,361 | 4.72 | 1.53 |
| **2016** | 1,262 | 34,167 | 109,368 | 147,647 | 4.17 | 1.35 |
| **2017** | 526 | 13,821 | 44,293 | 57,138 | 3.98 | 1.29 |
| **Average** | 1,102 | 34,456 | 109,717 | 164,728 | 4.55 | 1.48 |
