# Supplementary material for: Evaluating the cost of malaria elimination by Anopheles gambiae precision guided SIT in the Upper River region, The Gambia
Source: PLOS Glob Public Health. 2025 Jul 18;5(7):e0004903. doi: 10.1371/journal.pgph.0004903 (PMC12273942; doi:10.1371/journal.pgph.0004903)
Supplement: S40 Table — Treatment seeking costs saved from preventing malaria cases. (DOCX) [file pgph.0004903.s043.docx]

#### S40 Table: Treatment seeking costs saved from preventing malaria cases

| **Intervention Year** | **0-5 years** | **5-17 years** | **17-40 years** | **40-60 years** | **≥60 years** | **Total** |
| --- | --- | --- | --- | --- | --- | --- |
| **2** | 2,058 | 17,184 | 22,190 | 4,426 | 2,702 | 48,560 |
| **3** | 2,484 | 20,779 | 26,915 | 5,379 | 3,287 | 58,844 |
| **4** | 2,474 | 20,703 | 26,862 | 5,379 | 3,293 | 58,711 |
| **5** | 2,480 | 20,756 | 26,973 | 5,411 | 3,318 | 58,938 |
