# Supplementary material for: Evaluating the cost of malaria elimination by Anopheles gambiae precision guided SIT in the Upper River region, The Gambia
Source: PLOS Glob Public Health. 2025 Jul 18;5(7):e0004903. doi: 10.1371/journal.pgph.0004903 (PMC12273942; doi:10.1371/journal.pgph.0004903)
Supplement: S41 Table — Population Estimate and Willingness-to-pay for malaria prevention in the URR. (DOCX) [file pgph.0004903.s044.docx]

#### S41 Table: Population Estimate and Willingness-to-pay for malaria prevention in the URR

| **Year** | **Population Estimate** | **Willingness to Pay USD** |
| --- | --- | --- |
| **2022** | 300,149 | 4,541,254 |
| **2023** | 307,713 | 4,655,698 |
| **2024** | 315,467 | 4,773,016 |
| **2025** | 323,417 | 4,893,299 |
| **2026** | 331,567 | 5,016,609 |
| **2027** | 339,922 | 5,143,020 |
| **2028** | 348,488 | 5,272,623 |
| **2029** | 357,270 | 5,405,495 |
| **2030** | 366,273 | 5,541,710 |
| **2031** | 375,503 | 5,681,360 |
| **2032** | 384,966 | 5,824,536 |
| **2033** | 394,667 | 5,971,312 |
| **2034** | 404,613 | 6,121,795 |
| **2035** | 414,809 | 6,276,060 |
| **2036** | 425,262 | 6,434,214 |
| **2037** | 435,979 | 6,596,362 |
| **2038** | 446,966 | 6,762,596 |
| **2039** | 458,229 | 6,933,005 |
| **2040** | 469,777 | 7,107,726 |
