# Supplementary material for: Evaluating the cost of malaria elimination by Anopheles gambiae precision guided SIT in the Upper River region, The Gambia
Source: PLOS Glob Public Health. 2025 Jul 18;5(7):e0004903. doi: 10.1371/journal.pgph.0004903 (PMC12273942; doi:10.1371/journal.pgph.0004903)
Supplement: S42 Table — Current malaria intervention costs in The Gambia. (DOCX) [file pgph.0004903.s045.docx]

#### S42 Table: Current malaria intervention costs in The Gambia

|  | **SMC** | | | | **LLINs** | | | | **IRS** | | | | **IPTp** | | | | **Case Management** | | | |
| --- | --- | --- | --- | --- | --- | --- | --- | --- | --- | --- | --- | --- | --- | --- | --- | --- | --- | --- | --- | --- |
| **Year** | **2019** | **2020** | **2021** | **Average** | **2019** | **2020** | **2021** | **Average** | **2019** | **2020** | **2021** | **Average** | **2019** | **2020** | **2021** | **Average** | **2019** | **2020** | **2021** | **Average** |
| **Medicine & supplies** | **126,439** | **368,364** | **313,091** | **269,298** | **0** | **0** | **0** | **0** | **0** | **0** | **0** | **0** | **0** | **0** | **0** | **0** | **160,492** | **183,759** | **312,620** | **218,957** |
| **Insecticides & insecticide treated nets** | **0** | **0** | **0** | **0** | **117,217** | **13,172** | **4,750,235** | **1,626,874** | **146,401** | **85,038** | **441,871** | **224,437** | **0** | **0** | **0** | **0** | **0** | **0** | **0** | **0** |
| **Distribution remuneratio n** | **424,569** | **579,044** | **511,199** | **504,937** | **837,379** | **0** | **23,959** | **287,113** | **181,040** | **139,808** | **227,382** | **182,743** | **0** | **0** | **0** | **0** | **0** | **0** | **0** | **0** |
| **Management** | **158,226** | **0** | **0** | **52,742** | **251,865** | **258,535** | **146,990** | **219,130** | **0** | **0** | **0** | **0** | **0** | **0** | **0** | **0** | **0** | **0** | **0** | **0** |
| **Supervision** | **143,766** | **204,060** | **82,403** | **143,410** | **80,510** | **21,523** | **74,060** | **58,698** | **75,750** | **0** | **27,140** | **34,297** | **0** | **0** | **0** | **0** | **0** | **0** | **1,285** | **428** |
| **Meetings** | **0** | **11,000** | **8,689** | **9,845** | **64,539** | **0** | **0** | **21,513** | **0** | **0** | **0** | **0** | **0** | **0** | **3,189** | **1,063** | **0** | **0** | **0** | **0** |
| **Trainings** | **77,467** | **128,364** | **136,852** | **114,228** | **34,737** | **17,583** | **6,990** | **19,770** | **17,505** | **15,864** | **19,874** | **17,748** | **5,087** | **0** | **4,080** | **3,056** | **24,356** | **889** | **9,662** | **11,636** |
| **Social mobilization** | **7,722** | **20,311** | **28,850** | **18,961** | **0** | **0** | **0** | **0** | **12,659** | **14,828** | **11,824** | **13,104** | **0** | **0** | **0** | **0** | **0** | **0** | **0** | **0** |
| **Start-up costs (phones, solar systems)** | **0** | **22,659** | **13,180** | **11,946** | **244,010** | **0** | **0** | **81,337** | **27,347** | **0** | **28,178** | **18,508** | **2,855** | **0** | **3,435** | **2,097** | **0** | **0** | **0** | **0** |
| **Other (maintenance**  **, etc)** | **0** | **0** | **14,067** | **4,689** | **13,127** | **885** | **59,627** | **24,546** | **4,005** | **25,752** | **31,246** | **20,334** | **0** | **0** | **0** | **0** | **9,141** | **1,013** | **79,063** | **29,739** |
| **Total** | **938,189** | **1,333,802** | **1,108,330** | **1,130,055** | **1,643,383** | **311,698** | **5,061,862** | **2,338,981** | **464,706** | **281,290** | **787,516** | **511,171** | **7,492** | **0** | **10,704** | **6,215** | **193,989** | **185,661** | **402,631** | **260,760** |
