# Supplementary material for: Evaluating the cost of malaria elimination by Anopheles gambiae precision guided SIT in the Upper River region, The Gambia
Source: PLOS Glob Public Health. 2025 Jul 18;5(7):e0004903. doi: 10.1371/journal.pgph.0004903 (PMC12273942; doi:10.1371/journal.pgph.0004903)
Supplement: S43 Table — Cost per Case, DALY and Death Averted and Cost per Person Covered with current Interventions. These values are derived from Conteh et al and converted to 2022 USD to be directly comparable. (DOCX) [file pgph.0004903.s046.docx]

#### S43 Table: Cost per Case, DALY and Death Averted and Cost per Person Covered with current Interventions

These values are derived from Conteh et al and converted to 2022 USD to be directly comparable.

| **Intervention Method (Median Cost)** | **Cost per Case Averted** | **Cost per DALY Averted** | **Cost per Death Averted** | **Cost per Person Covered** |
| --- | --- | --- | --- | --- |
| ITN/LLIN | 7.72 | 58.75 | 1,692.23 | 1.83 |
| IRS | N/A* | 33.20^#^ | 1,109.39^#^ | 7.52 |

* The IRS spray did not have primary costing data in regards to cases averted as of the publication of Conteh et al. (2019)

^#^ Costs for these were derived from one paper and were presented as a range. The high and low estimates were averaged to simplify the comparison.
