# Supplementary material for: Evaluating the cost of malaria elimination by Anopheles gambiae precision guided SIT in the Upper River region, The Gambia
Source: PLOS Glob Public Health. 2025 Jul 18;5(7):e0004903. doi: 10.1371/journal.pgph.0004903 (PMC12273942; doi:10.1371/journal.pgph.0004903)
Supplement: S1 Text — (DOCX) [file pgph.0004903.s047.docx]

**Supporting Information for “Evaluating the cost of malaria elimination by Anopheles gambiae precision guided SIT in the Upper River region, The Gambia”**

William A. C. Gendron^1^, Robyn Raban^1^, Agastya Mondal^2^, Héctor M. Sánchez C.^2^, Andrea L. Smidler^1^, David Zilberman^3^ , Patrick G. C. Ilboudo^4,5^, Umberto D’Alessandro^6^, John M. Marshall^2,7^, Omar S. Akbari^1,^ ^†^

#### Affiliations

^1^School of Biological Sciences, Department of Cell and Developmental Biology, University of California, San Diego, La Jolla, CA 92093

^2^Divisions of Epidemiology & Biostatistics, School of Public Health, University of California, Berkeley, CA 94720, USA

^3^Department of Agricultural and Resource Economics, University of California, Berkeley, CA 94720, USA

^4^Chronic Diseases Management Unit, African Population and Health Research Center, Nairobi, Kenya

^5^Health Economics and Financing Division, Africa Centres for Disease Control and Prevention, Addis Ababa, Ethiopia, (Current Address)

^6^Medical Research Council Unit The Gambia at the London School of Hygiene and Tropical Medicine, Fajara, The Gambia

^7^Innovative Genomics Institute, University of California, Berkeley, CA 94720, USA

^†^To whom correspondence should be addressed:

Omar S. Akbari, Ph.D.

School of Biological Sciences, Department of Cell and Developmental Biology, University of California, San Diego, La Jolla, CA 92093, USA

Ph: 858-246-0640

**Email**: [oakbari@ucsd.edu](mailto:oakbari@ucsd.edu)

**Table of Contents:**

1. [**Cost estimate of implementing pgSIT in The Gambia** **4**](#_heading=h.afgymdghv23a)
   1. [Research and Development Costs 4](#_heading=h.hi30s4w8ullx)
      1. Development of pgSIT 2.0 to support COPAS sex sorting 4
      2. Field testing and small-scale release overview 5
      3. Initial cage trials and small-scale release preparation 5
      4. Small-scale field trials 6
      5. Large-scale field trials 7
      6. Regulatory approvals for large-scale deployment 8
   2. [**Mosquito rearing phases** **8**](#_heading=h.z5scchghxxy7)
      1. Maintenance phase 9
      2. Ramping phase 9
      3. Active Phase 9
   3. [**Estimating mosquito production requirements** **9**](#_heading=h.1r6zx04drjux)
      1. Production design and modifications 10
      2. Weekly egg production estimates for The Upper River region 10
      3. Colony production requirements to meet weekly egg production 11
      4. Mosquito survival 11
      5. Sorting technology impact on mosquito production 12
      6. Egg isolation and hatching methodology 12
      7. Egg isolation and hatching equipment costs 12
      8. [COPAS-related costs 13](#_heading=h.2of4berusazr)

1.3.7a COPAS overview 13

- - 1. b COPAS FP 500 Initial Cost 13
    2. c COPAS FP 500 annual cost 14
    3. a Larvae rearing rack and tray system overview 14
    4. b Larvae rearing rack and tray system costs 15
    5. a Water and larval food overview 16
    6. b Water and larval feed annual costs 17
    7. a Pupae isolation and transfer overview 18
    8. b Pupae isolation, transfer, and adult cage costs 18
    9. a Adult mosquito blood feeding overview 18
    10. b Upfront adult mosquito blood feeding and egg harvesting costs 19
    11. a Drone delivery of pgSIT eggs to the Upper River region overview 20
    12. b Costs of drone delivery of pgSIT eggs in the Upper River region, storage costs and delivery considerations 20

1.2.13a Mosquito release in the Upper River region overview 21

- - 1. b Mosquito release in the Upper River region costs 21
    2. pgSIT line stability and quality control 23
    3. a Entomological and epidemiological monitoring overview 24
    4. b Entomological and epidemiological monitoring costs 24
    5. a Facility construction and supporting systems 25
    6. Initial facility training 25
    7. Facility annual labor 26
    8. Facility costs summarized 26

1. **Predicted health benefits of implementing pgSIT in The Upper River Region of The Gambia.. 27**
   1. Life year calculations and life years saved annually 27
   2. Sick day calculations and sick days saved annually 27
2. [**Quantifying the economic benefits of pgSIT in The Gambia** **27**](#_heading=h.10p0eqwidzxw)
   1. Value of statistical life (VSL) saved per year 28
   2. Value of life years based on quality adjusted life years (QALY) saved annually 29
   3. GDP growth benefit associated with malaria prevention 29
   4. Value of sick days saved per year 30
   5. Value of medical intervention saved per year 30
   6. Value of IRS and LLINs saved per year 30
   7. Willingness-to-pay value per year 31
   8. Additional economic benefits 31
3. [Current Intervention Costs Locally and Cost per DALY Averted of Current Interventions 32](#_heading=h.h8yhy1y6shy)
   1. Current local intervention costs 32
   2. Comparing Current Interventions to pgSIT Estimates 32

[**S1 Fig. Seasonal rainfall profile for Upper River region, The Gambia** **33**](#_heading=h.ddhe6pxaf7xv)

[**S2 Fig. Phased testing pathway for genetically modified mosquitoes** **34**](#_heading=h.nopqvi30t8t7)

[**S3 Fig. Mass rearing during the facility’s active phase** **35**](#_heading=h.x0k82ei8n7r3)

**List of Supplemental Figures and Tables:**

[**S1**](https://docs.google.com/document/d/1m60RsD8x2eVAo-TNUYbVCRb3Vq2famH5uVdhOneK2NY/edit#heading=h.qpvw64q3666d) **Fig. Seasonal rainfall profile for Upper River region, The Gambia.**

[**S2**](https://docs.google.com/document/d/1m60RsD8x2eVAo-TNUYbVCRb3Vq2famH5uVdhOneK2NY/edit#heading=h.rzuczgt8zjqh) **Fig. Phased testing pathway for genetically modified mosquitoes.**

[**S3**](https://docs.google.com/document/d/1m60RsD8x2eVAo-TNUYbVCRb3Vq2famH5uVdhOneK2NY/edit#heading=h.35akpidgltd) **Fig: Mass rearing during the facility’s active phase.**

**S1 Table Parameters used in mathematical modeling**

[**S2**](https://docs.google.com/document/d/1m60RsD8x2eVAo-TNUYbVCRb3Vq2famH5uVdhOneK2NY/edit#heading=h.k6jkiqs3v1h0) **Table: Annual cost of introgression experiments**

[**S3**](https://docs.google.com/document/d/1m60RsD8x2eVAo-TNUYbVCRb3Vq2famH5uVdhOneK2NY/edit#heading=h.msn4dm23m2yu) **Table: Total budget estimate of introgression and initial cage trials**

[**S4**](https://docs.google.com/document/d/1m60RsD8x2eVAo-TNUYbVCRb3Vq2famH5uVdhOneK2NY/edit#heading=h.d08mqw43tgjz) **Table: Total budget estimate of small scale trial**

[**S5**](https://docs.google.com/document/d/1m60RsD8x2eVAo-TNUYbVCRb3Vq2famH5uVdhOneK2NY/edit#heading=h.yopkdxn2d0jj) **Table: Total budget estimate for large scale field trials**

[**S6**](https://docs.google.com/document/d/1m60RsD8x2eVAo-TNUYbVCRb3Vq2famH5uVdhOneK2NY/edit#heading=h.v8qkqca9myb8) **Table: Monitoring costs for the first 5 years of mosquito releases**

[**S7**](https://docs.google.com/document/d/1m60RsD8x2eVAo-TNUYbVCRb3Vq2famH5uVdhOneK2NY/edit#heading=h.iujlwzvfi7cc) **Table: Total adult mosquitoes required for egg production.**

[**S8**](https://docs.google.com/document/d/1m60RsD8x2eVAo-TNUYbVCRb3Vq2famH5uVdhOneK2NY/edit#heading=h.sgoe3qegk9ka) **Table: COPAS FP 500 larvae daily rearing requirements.**

[**S9**](https://docs.google.com/document/d/1m60RsD8x2eVAo-TNUYbVCRb3Vq2famH5uVdhOneK2NY/edit#heading=h.fxf8pyochnyr) **Table: Total initial costs with least expensive trials**

**S10Table: Total initial costs with more expensive trials**

[**S11**](https://docs.google.com/document/d/1m60RsD8x2eVAo-TNUYbVCRb3Vq2famH5uVdhOneK2NY/edit#heading=h.1k7t5xj85z9q) **Table: Cost of COPAS FP 500 and annual service fee**

[**S12**](https://docs.google.com/document/d/1m60RsD8x2eVAo-TNUYbVCRb3Vq2famH5uVdhOneK2NY/edit#heading=h.epydc0c80cvy) **​​Table: COPAS sex sorting rack and tray numbers, cost and expected maintenance fees**

[**S13**](https://docs.google.com/document/d/1m60RsD8x2eVAo-TNUYbVCRb3Vq2famH5uVdhOneK2NY/edit#heading=h.oi23l9lw4xe5) **Table: Annual water usage and cost**

[**S14**](https://docs.google.com/document/d/1m60RsD8x2eVAo-TNUYbVCRb3Vq2famH5uVdhOneK2NY/edit#heading=h.6i64ukamq524) **Table: Cost per liter of larval food**

[**S15**](https://docs.google.com/document/d/1m60RsD8x2eVAo-TNUYbVCRb3Vq2famH5uVdhOneK2NY/edit#heading=h.nn7wccxy7q2l) **Table: Larval food requirements and cost**

[**S16**](https://docs.google.com/document/d/1m60RsD8x2eVAo-TNUYbVCRb3Vq2famH5uVdhOneK2NY/edit#heading=h.radcwsor6bqt) **Table: Adult mosquito cage costs**

**S17 Table:Fresh blood estimate: utilizing locally sourced blood.**

[**S18**](https://docs.google.com/document/d/1m60RsD8x2eVAo-TNUYbVCRb3Vq2famH5uVdhOneK2NY/edit#heading=h.pl21ayrxt98i) **Table: Hemotek device cost and annual fees**

[**S19**](https://docs.google.com/document/d/1m60RsD8x2eVAo-TNUYbVCRb3Vq2famH5uVdhOneK2NY/edit#heading=h.f7vaf7o6wm9h) **Table: Drone costs and annual fees**

[**S20**](https://docs.google.com/document/d/1m60RsD8x2eVAo-TNUYbVCRb3Vq2famH5uVdhOneK2NY/edit#heading=h.27y0v6slyggz) **Table: The costs of rearing larvae in The Upper River region**

[**S21**](https://docs.google.com/document/d/1m60RsD8x2eVAo-TNUYbVCRb3Vq2famH5uVdhOneK2NY/edit#heading=h.nc9r4iu3zdww) **Table: Mutation rates and quality control**

[**S22**](https://docs.google.com/document/d/1m60RsD8x2eVAo-TNUYbVCRb3Vq2famH5uVdhOneK2NY/edit#heading=h.ugzi5gsae45k) **Table: Monitoring costs for the first 5 years of mosquito releases**

[**S23**](https://docs.google.com/document/d/1m60RsD8x2eVAo-TNUYbVCRb3Vq2famH5uVdhOneK2NY/edit#heading=h.j07apopiazut) **Table: Banjul Land Cost Estimate**

[**S24**](https://docs.google.com/document/d/1m60RsD8x2eVAo-TNUYbVCRb3Vq2famH5uVdhOneK2NY/edit#heading=h.2w5l4nypftnu) **Table: Minimum facility size, land, and cost**

[**S25**](https://docs.google.com/document/d/1m60RsD8x2eVAo-TNUYbVCRb3Vq2famH5uVdhOneK2NY/edit#heading=h.e06tcu2woop6) **Table: High wage annual estimate**

[**S26**](https://docs.google.com/document/d/1m60RsD8x2eVAo-TNUYbVCRb3Vq2famH5uVdhOneK2NY/edit#heading=h.ql1fd2wlsnwk) **Table: Medium wage annual estimate**

[**S27**](https://docs.google.com/document/d/1m60RsD8x2eVAo-TNUYbVCRb3Vq2famH5uVdhOneK2NY/edit#heading=h.hoty9a7b89kq) **Table: Low wage annual estimate**

[**S28**](https://docs.google.com/document/d/1m60RsD8x2eVAo-TNUYbVCRb3Vq2famH5uVdhOneK2NY/edit#heading=h.4u00rorvs46r) **Table: Total initial costs with least expensive trials**

[**S29**](https://docs.google.com/document/d/1m60RsD8x2eVAo-TNUYbVCRb3Vq2famH5uVdhOneK2NY/edit#heading=h.3lar46fqdcez) **Table: Total initial costs with more expensive trials**

**S30Table: Total annual costs**

[**S31**](https://docs.google.com/document/d/1m60RsD8x2eVAo-TNUYbVCRb3Vq2famH5uVdhOneK2NY/edit#heading=h.t7ghvudtlmy4) **Table: Estimated age stratified life years saved**

[**S32**](https://docs.google.com/document/d/1m60RsD8x2eVAo-TNUYbVCRb3Vq2famH5uVdhOneK2NY/edit#heading=h.w4jogd9j0rde) **Table: Life years saved annually**

[**S33**](https://docs.google.com/document/d/1m60RsD8x2eVAo-TNUYbVCRb3Vq2famH5uVdhOneK2NY/edit#heading=h.q4jhszehuswo) **Table: Sick days prevented by age over four years of pgSIT interventions**

[**S34**](https://docs.google.com/document/d/1m60RsD8x2eVAo-TNUYbVCRb3Vq2famH5uVdhOneK2NY/edit#heading=h.3c7vpgdsohf2) **Table: Value of statistical life calculations**

[**S35**](https://docs.google.com/document/d/1m60RsD8x2eVAo-TNUYbVCRb3Vq2famH5uVdhOneK2NY/edit#heading=h.y8dx6q97ls2) **Table: Quality adjusted life year calculations**

[**S36**](https://docs.google.com/document/d/1m60RsD8x2eVAo-TNUYbVCRb3Vq2famH5uVdhOneK2NY/edit#heading=h.icj5f0yxl7k7) **Table: GDP growth estimate**

[**S37**](https://docs.google.com/document/d/1m60RsD8x2eVAo-TNUYbVCRb3Vq2famH5uVdhOneK2NY/edit#heading=h.u5kjurn3uxeb) **Table: Value of sick days saved**

[**S38**](https://docs.google.com/document/d/1m60RsD8x2eVAo-TNUYbVCRb3Vq2famH5uVdhOneK2NY/edit#heading=h.dnfh5gt66wv) **Table: Annualized mean costs of the interventions against malaria in the Upper River Region (in 2021 USD)**

[**S39**](https://docs.google.com/document/d/1m60RsD8x2eVAo-TNUYbVCRb3Vq2famH5uVdhOneK2NY/edit#heading=h.y4mhbs3fftfa) **Table: Costs associated with malaria treatment seeking**

[**S40**](https://docs.google.com/document/d/1m60RsD8x2eVAo-TNUYbVCRb3Vq2famH5uVdhOneK2NY/edit#heading=h.n97djwbcxdxv) **Table: Treatment seeking costs saved from preventing malaria cases**

[**S41**](https://docs.google.com/document/d/1m60RsD8x2eVAo-TNUYbVCRb3Vq2famH5uVdhOneK2NY/edit#heading=h.ay9wckbvvdtt) **Table: Population Estimate and Willingness-to-pay for malaria prevention in the URR**

[**S42**](https://docs.google.com/document/d/1m60RsD8x2eVAo-TNUYbVCRb3Vq2famH5uVdhOneK2NY/edit#heading=h.7zwkbdvat5wb) **Table: Current malaria intervention costs in The Gambia**

[**S43**](https://docs.google.com/document/d/1m60RsD8x2eVAo-TNUYbVCRb3Vq2famH5uVdhOneK2NY/edit#heading=h.49ddzqa2xiaz) **Table: Cost per Case, DALY and Death Averted and Cost per Person Covered with current Interventions**

**Supporting Information Text:**

## Cost estimate of implementing pgSIT in The Gambia

#### Research and Development Costs

Prior to production, the *A. gambiae* pgSIT has to (1) integrate sex specific markers into the pgSIT 1.0 lines to facilitate COPAS sex sorting technologies (pgSIT 2.0 with fluorescent sex specific markers), (2) evaluate pgSIT 2.0 in small scale field testing and (3) obtain regulatory approvals for small- and large-scale field testing. The pgSIT system (pgSIT 1.0) currently in our lab shows 100% female-killing and 100% male sterilization among many thousands of insects tested, demonstrating proof-of-principle of the technology in the species [[1]](https://paperpile.com/c/JoQtIv/ZftFg). However, these pgSIT 1.0 lines lack a sex-selection marker making optical sorting the only available sorting technology

amenable to this system. Therefore, we will focus on releasing the pgSIT 2.0 technology, which integrates genetic sex sorting with male sterilization.

- - 1. ​Development of pgSIT 2.0 to support COPAS sex sorting

The pgSIT technology has been developed in *A. gambiae*[*[1]*](https://paperpile.com/c/JoQtIv/ZftFg), but these lines need to be integrated with sex-specific fluorescent markers to support the COPAS sex sorting technology. COPAS requires these markers to separate the sexes, a critical step for establishing the parental crosses. Fortunately, we have just developed a robust sex-sorting approach termed SEPARATOR (Sexing Element Produced by Alternative RNA-splicing of A Transgenic Observable Reporter) that exploits sex-specific alternative splicing of an innocuous reporter to ensure exclusive dominant male-specific expression [[2]](https://paperpile.com/c/JoQtIv/j4ptL). To use SEPARATOR to scale pgSIT production, we will, therefore, need to redesign our pgSIT lines to incorporate SEPARATOR, termed pgSIT 2.0, and evaluate these lines for sterile male production.

The primary cost to update this line is labor and resources for this system to be created and to confirm the efficacy of this line using standard laboratory practices. The approximate costs to cover this work correspond to the cost of one postdoctoral scholar, two or three dedicated paid research assistants, research supplies, and overhead. It may take one to two years to complete the creation and assessment of these lines.

- - 1. ​Field testing and small-scale release overview

Prior to large-scale release in The Gambia, field testing and small-scale releases will be required to obtain safety and efficacy data, regulatory approval, and community support for large-scale release. These small-scale trials also allow testing and optimization of the mass rearing technologies. There are three phases in small-scale testing: (1) initial cage trials and preparation (Phase 2, **S2 Fig**), (2) small-scale releases, and (3) functional scale release (Phase 3, **S2 Fig**). The scaling of this technology will also be tested, and stakeholders and the community will be involved in this development process.

- - 1. ​Initial cage trials and small-scale release preparation

Cage trials in The Gambia will evaluate the pgSIT technology in the local environment and genetic background by introgressing the pgSIT into local strains. These studies will provide the efficacy data required for small- and large-scale releases, community engagement opportunities to gain input and buy-in for field releases, and will evaluate and optimize rearing procedures and technologies required to scale up egg production for field releases. These initial experiments will require small-scale facilities and are dependent on procuring necessary permits and authorizations.

Preparing for the small-scale release will require engagement with local communities, The Gambian government, and other stakeholders to obtain their feedback and approval to advance the

project to field release. We plan to incorporate many of the core commitments we developed for our gene drive technologies, such as commitments towards fair partnership and transparency in the evaluation and development of the pgSIT technology and field trials, product efficacy and safety, regulatory evaluation, risk and benefit assessment, trial monitoring, and mitigation [[3]](https://paperpile.com/c/JoQtIv/4UxDP) to strive for transparency and inclusion of stakeholder perspectives in the development of our trials. We also benefit from the experience of other groups bringing other mosquito technologies to the field [[4]](https://paperpile.com/c/JoQtIv/BI07S) and from the experience of our partners working with the communities in the URR. Ultimately, these cage trials will provide the data needed to support small field releases and are an important step to obtaining feedback from the local community and other stakeholders.

The most cost effective approach to the cage trial is to contract this work to organizations with extensive expertise in *A. gambiae* rearing and has led multiple field trials across West Africa. For example, the London School of Hygiene and Tropical Medicine (LSHTM) has this expertise and a field laboratory in Basse, The Gambia. Otherwise, there may be additional infrastructure and equipment costs in a less established area. A rough estimate would be to assume the need for one postdoctoral scholar or research fellow to lead this project, laboratory technicians, and resources to complete these experiments. A research fellow at the LSHTM in The Gambia is paid at most 71,000 USD annually, and local lab technicians are paid approximately 4,500 USD annually (**S2 Table**)(per communication with LSHTM). One fellow and three technicians are required for this work. This project would take two to three years to complete introgression and cage trials(**S2 Table**).

During this stage, minimal equipment is needed for mass rearing. This equipment is one to two Wolbaki mass rearing racks and tray systems (22,500 USD each, quote procured through communication with Guangzhou Wolbaki Biotech Co., Ltd., China,**S3 Table**) and at least four large cages. Cages used for cage trials vary across papers, but it is possible that the mass rearing adult cages could be utilized for the field trials, and these cost 250 USD each (**S3 Table**)[[5]](https://paperpile.com/c/JoQtIv/UsiJ). There may be an additional cost of a fluorescent microscope to screen the mosquitoes, which can range in price from 2,000 to 20,000 USD, but a microscope at the 10,000 USD price point will likely suffice (**S3 Table**). This price was selected as secondhand microscopes go for significantly less when looking at available options at various refurbishing groups such as Microscope Central and 10,000 USD should allow for plenty of flexibility and unexpected increases in price. The cost of the raw materials would consist of mosquito feed, water, large cages for outdoor cage rearing, basic laboratory equipment such as thermocyclers, and sequencing costs for confirming species introgression. This facility will be rearing up to two racks of mosquitoes year-round, so we expect at most 3,325 USD annually in food and water costs (**S2 and S3 Tables**). There will be extensive work monitoring the introgression and to simplify the cost, we assigned a budget of 150,000 USD(based on previous experience with similar work by the co-authors) to troubleshoot problems and to perform sequencing and other assays associated with introgressing mosquitoes into the local genetic background. A conservative estimate of these total costs will be approximately 977,975 USD (**S3 Table**). These costs may be an overestimate, but the development and testing of this technology will likely be when the project encounters unknown and unaccounted for issues that may require extra funding to address.

- - 1. ​Small-scale field trials

Pending the necessary approvals and regulatory guidance, the likely next step is a small-scale trial to confirm the efficacy and safety of pgSIT sterile males in the field (Phase 3, **S2 Fig**). These studies will assess pgSIT mosquito movement, distribution, longevity, and any unexpected introgression of pgSIT genes into the local mosquito population. These studies will include preliminary research on the entomological impact of the technology, but this phase will focus on the safety and risk assessment information required for large-scale studies, which can be easily acquired with minimal releases.

Small-scale releases are also useful for the initial testing and optimizing mass scale rearing. We can evaluate the proposed equipment and procedures, for example, to obtain better survival and fecundity rate estimates, which will allow us to more accurately estimate the upfront and annual facility costs and the resources required for scaling egg production for mass release. The egg production needs of a small-scale study may be too low to necessitate sex sorting technologies, but we may be able to demo these systems or begin optimizing the sex sorting technologies in the larger-scale release phase.

Small-scale field trials can build off equipment, resources, knowledge, and expertise gained during the cage trial. The primary additions to the budget will be resources for release monitoring and potentially purchasing sex sorting devices for a total budget estimate of 983,610 USD (**S4 Table**) (Costing per communication with Union Biometrica). The small-scale field trial can utilize the mass rearing racks and cages and should take one to two years. There may be some initial monitoring experiments the year before the small-scale release to gauge the native mosquito population abundance and distribution. Pre-release monitoring can also facilitate site selection and identify controls for the pilot study.

- - 1. ​Large-scale field trials

Large-scale releases provide additional safety and essential efficacy data to evaluate pgSIT sterile male suppression of mosquito populations. During the cage studies and small-scale releases, we will build relationships with the community and other stakeholders and accumulate data from our studies, local health centers, and vector management organizations to identify multiple areas for larger-scale trials. Community members will also become more involved in the rearing of sterile male mosquitoes from eggs to adulthood.

The scale of these studies will be informed by the data collected during the cage and small field trials and data and input from local stakeholders. As the scale of these studies increases, it will require moderate scaling of egg production. A village in the URR, for example, may have a population of approximately 1,000 people, so manual mosquito sorting is no longer manageable, as about 58,000 larvae will need to be sorted daily. During this phase, a COPAS FP 500 or another sorting technology will need to be implemented to support egg production. This approach will allow us to test and optimize sex sorting technologies in the field. In particular, these sorting devices should be pushed to their limits to determine their capabilities and stress-test their hardware.

The cost of the large-scale field trial can utilize any devices purchased during the small-scale field trial. The costs are primarily the same with small changes to address the increase in scale (**S5 Table**). Additional fees will be needed to maintain the COPAS FP 500 (36,960 USD) and the monitoring and releases should continue for two years, which is accounted for in the budget (**S5 Table**)(Pricing from communication with Union Biometrica). One-to-two-month seasonal workers were also added to support the mosquito field collections.

Depending on the results of the previous studies and technical needs, a larger scale cluster randomized trial similar to the recent trial in the URR[[6]](https://paperpile.com/c/JoQtIv/rcYb) could also be implemented[[7]](https://paperpile.com/c/JoQtIv/Lltc). A large-scale cluster randomized trial in the region costs approximately 2.5 million USD. This cost is included in the **S6 Table** and would increase the initial cost of development but would not have long term effects on the annual costs. There are outstanding questions about the scale and design of the field trials, but as the pgSIT technology advances in development, we will acquire the data to address these questions.

- - 1. Regulatory approvals for large-scale deployment

Throughout the field trials and the transition to large-scale use, we will engage regulatory stakeholders to ensure compliance with the relevant laws and regulations. The Gambia has yet to release genetically engineered (GE) mosquitoes, and has not yet established laws and governance for such activities. The Gambia is expected to follow guidelines set out by the Cartagena Protocol on Biosafety. Organizations such as Target Malaria in other African regions may have paved the road for future GE mosquito releases on the continent. Still, there is also the possibility that our project may prompt the creation of a country-specific governance or a regulatory body in The Gambia. No matter the approval process, frequent engagement with regulators early and throughout this process should establish the regulatory pathway for the pgSIT technology. Other GE mosquito releases have occurred elsewhere in Africa and the rest of the world, so there is a precedent for these releases. Once the regulatory requirements are better defined, additional costs may be required to support regulatory compliance activities. Because the development of regulations can take time, we plan to engage the Gambian government early in the project planning to identify the best path toward regulatory approval.

#### Mosquito rearing phases

The pgSIT mosquitoes do not need to be constantly released throughout the year to reduce mosquito abundance and malaria transmission, since *A. gambiae* in the URR and many other regions throughout their range only have seasonally elevated populations (in The Gambia this is the rainy season beginning June 1^st^- late October). The rest of the year, the *A. gambiae* population size is too low to sustain malaria transmission. To minimize the cost of rearing mosquitoes for a large-scale facility, the facility’s year can be divided into 3 phases for consideration: Maintenance Phase (34 weeks), Ramping Phase (6 weeks), and Active Phase (12 weeks). The Maintenance Phase is designed to keep a minimal population of the parent Cas9 and gRNA lines alive and reproducing to minimize labor. The Ramping Phase is used to rapidly increase the number of mosquitoes to the numbers required to meet the egg production needs in the Active Phase while spreading the daily egg batches across three weeks to maximize the facility's efficiency. The

release schedule is based on weekly releases of eggs to parts of The Upper River. Functionally, this production will be spread throughout the week rather than shipping the eggs once a week. The Active Phase is the full sterile egg production phase for delivering sterile male pgSIT mosquitoes to the field sites. During the active phase, the facility runs near maximum capability with contingencies to account for equipment failures or distribution problems. Upon completion of the Active phase, at the end of the mosquito season, mosquitoes are culled down to Maintenance Phase numbers.

To time the releases with the beginning of the rainy season, the Active Phase egg hatching should start on May 16th, two weeks before June 1st. The Active Phase will continue to produce daily batches until 14 weeks later, on August 22nd. This last batch can be culled to Maintenance level production on approximately September 12th. There may be a need to stagger egg production by a couple of days to align the Maintenance Phase schedule to the Ramping Phase Schedule. The first Ramping Phase would need to occur nine weeks before the start of the Active Phase. March 14th will be the approximate start of the last Maintenance Phase, where there will be three egg harvesting cycles to begin the shift to the Ramping Phase. To prepare for the beginning of the Active Phase on May 16th, April 4th is the approximate date of the beginning of the Ramping Phase. This cycle will repeat annually.

- - 1. ​Maintenance phase

The Maintenance Phase was designed to minimize cost while sustaining a large, stable population for expansion during the Ramping Phase. This option has been outlined for larger scale production or if further reducing maintenance phase work is desirable. Synchronization of the mosquito life cycle in the Maintenance Phase production will necessitate a Ramping Phase to spread production over a week to minimize the burden on COPAS sex sorting and other activities. The maintenance phase minimizes the required work hours to maintain the facility and the resources used to sustain the mosquito population. Despite minimizing work hours, we assume salaried positions for all workers to maintain a trained workforce year to year.

- - 1. ​Ramping phase

When transitioning from the Maintenance to the Active Phase, a six week Ramping Phase is required to build the colony to support the egg production numbers required in the Active Phase. While this facility does not need six weeks to expand the colony to meet the production requirements for the Active Phase, the six week Ramping Phase is necessary to synchronize daily production. This ramp-up of the colony is accomplished by increasing the blood feeding and egg harvesting frequencies to spread egg production over three days per week. This strategy will provide eggs over nine 3-week cycles that can be expanded to 27 individual cycles at the end of the Ramping Phase. At this point, eggs will be produced daily, providing complete coverage for the Active Phase. To calculate rearing costs, we will treat this phase as if it were the active phase.

- - 1. ​Active Phase

The Active Phase has a new cycle (or cohort) of mosquitoes being hatched and sorted daily. Importantly, this schedule minimizes the number of COPAS sorting machines required to produce the weekly egg requirements. The COPAS is the highest cost equipment for this project and is limited in the number of larvae that can be sorted in 24 hours. Therefore, it is essential to maximize the efficiency of each machine. To this end, production is staggered across every day during the full three week cycle. A daily production schedule also reduces the number of rearing racks, cages, and drones needed to obtain production numbers.

#### Estimating mosquito production requirements

- - 1. ​Production design and modifications

*Anopheles gambiae* production plans were developed from the laboratory mass rearing protocols established by the International Atomic Energy Agency (IAEA)[[8]](https://paperpile.com/c/JoQtIv/VU3fE). We further modified the protocol to accommodate the differences between pgSIT production requirements and the standard protocols. The two most notable modifications are the use of automated fluorescent-marker based sex sorting of L1 larvae [[9]](https://paperpile.com/c/JoQtIv/ltnjc) and the release of pgSIT eggs rather than adults. Manual sorting is possible, but automating sex sorting will better support the scaling and throughput needed for mass mosquito production, PgSIT also allows for the release of eggs, which has significant advantages to mosquito fitness and delivery over releasing fragile adult mosquitoes which is the standard. With up to 200 mile flights to service the URR, delivery of adult mosquitoes may not be feasible. The IAEA production protocol is therefore adjusted for egg delivery.

When eggs are delivered to field sites they also need to be raised to adulthood, which also does not have a direct comparison as SIT, Wolbachia and RIDL interventions release adults. With a lack of data on field production of adults, we selected the two most divergent options to capture the full range of costs; 1) Fully supported mosquito production using laboratory conditions and costs or 2) local release without rearing support, which assumes that mosquitoes will survive in the local environment. Costs were calculated for both scenarios, and likely the field rearing requirements will be somewhere in between.

- - 1. ​Weekly egg production estimates for The Upper River region

Estimates of the total egg production are needed to determine the facilities and production capabilities required to meet this demand. The mathematical modeling estimates a release scenario of 32 eggs per adult *A. gambiae* mosquito per week, but this number is dependent on the total number of mosquitoes in The URR. With a population of approximately 265,000 people

[[10]](https://paperpile.com/c/JoQtIv/ZF5D5) and an estimate of ~8 mosquitoes per person on average (~2.6 per person in the dry season and ~25 per person at the peak of the rainy season), we estimated the number of adult mosquitoes required per week. The estimates of mosquitoes per person are calculated based on the malaria prevalence in the population in 2017 and the number of mosquitoes that would need to be present

to support this rate of malaria. This indirect estimate is based on the malaria rates and population size, but this is a useful approximation as the URR lacks extensive direct entomological studies [[6]](https://paperpile.com/c/JoQtIv/rcYb). These estimates give us an *A. gambiae* population of approximately 1.9 million adult mosquitoes during the beginning of the rainy season. These numbers (1.9 million mosquitoes and 32 eggs released per mosquito) are multiplied to give us a weekly requirement of 60.8 million eggs. Producing eggs simultaneously is not the most efficient use of the equipment. It puts unnecessary strain on the staff, resources, and equipment. To address this, the production of these 60.8 million eggs can be spread across the entire week. This approach gives us a daily egg requirement of just under 8.7 million eggs (**S7 Table**). The daily egg production can be used to determine the number of adult mosquitoes required to produce the requisite eggs and the infrastructure needed to support this level of mosquito production.

- - 1. ​Colony production requirements to meet weekly egg production

To meet the 8.7 million eggs daily egg production needs (Section 2.1.1, we worked backward to estimate the number of females and total mosquitoes required to achieve these numbers. We accounted for the uncertainties in fecundity (**S7 Table**), larval mortality (**S8 Table**), and sex selection technologies (**S8 Table**) to give us a range of cost estimates. For example, female *A. gambiae* in the wild can lay 800-1,000 eggs in their lifetime [[11]](https://paperpile.com/c/JoQtIv/x3SMC). This amount is unlikely achievable in mass rearing or even normal laboratory conditions. A conservative estimate of lifetime fecundity per *A. gambiae* female is 300 eggs. This estimate is calculated by multiplying the average number of eggs laid per female during the first oviposition (162 eggs), and the hatch rate of 86% and the average percentage of females that lay eggs per oviposition cycle (85%) [[12–14]](https://paperpile.com/c/JoQtIv/GYbvj%2BjJwH0%2B5dAR3). This means that at first oviposition, each female produces an average of 118 eggs. As the females age, their fecundity at each oviposition cycle decreases [[13]](https://paperpile.com/c/JoQtIv/jJwH0). Using a moderate estimate of a 15% decrease between each egg lay, the second and third oviposition cycles are expected to produce 101 and 86 eggs, respectively. The 15% decrease is a conservative estimate of *A. gambiae* female survival across the two weeks of adulthood, which is usually above 70% survival. An estimated 15% death rate between blood feedings, however, results in the conservative estimate of 60% survival to the end of the two weeks of adulthood. This results in 306 mosquitoes per adult female, which we round down to 300. With careful design and rearing experience, we expect to exceed or at least maintain this fecundity rate. However, we are also including the 30% reduced fecundity rate of 210 eggs per female to provide a worst-case scenario estimate. These calculations assume approximately a 65% initial blood feeding rate and a further 35% decline between each feeding. The ideal mating ratio is 1:1 according to the IAEA Protocol [[15]](https://paperpile.com/c/JoQtIv/JPcz), so we double the number of females to meet the daily requirement of adult mosquitoes.

- - 1. ​Mosquito survival

To produce the number of adult mosquitoes needed for egg production, we also factored in mosquito survival. In laboratory conditions, the larval survival rate can be quite high, but in a mass-rearing system where rearing densities are high, larval survival is lower. While we have not evaluated pgSIT larval survival on a mass scale, the IAEA *A. gambiae* mass rearing guidelines suggest that larval to adult survival in the high density larval mass rearing systems range from 50-75% [[15]](https://paperpile.com/c/JoQtIv/JPcz). The upper and lower range of these survival rates were included in our calculations. To

maintain the colony, a minimum of 2.2% of the eggs produced must be used to replenish the lines but to be conservative, we estimate that 3% is needed to replenish the colony. While this would be sufficient to support the colony, we also want to ensure genetic diversity in the stock and therefore we expand this estimate to 6% of the mosquito production. If producing mosquitoes in a higher quantity, 3% could be sufficient to maintain production during the Active Phase. The 2.2% estimate is derived from the least efficient production scenarios (females produce 210 eggs, 50% larvae to adult survival) (**S8 Table)**. If each female mosquito produces 210 eggs, each adult (males and females) produces 105 eggs. Including an 86% egg hatch rate and a 50% larva to adult survival rate, results in 45.15 adult mosquitoes produced per adult mosquito in mass rearing conditions [[14]](https://paperpile.com/c/JoQtIv/5dAR3). To convert this estimate to percentages, we divide 100 by the number of adult mosquitoes produced per adult mosquito to get 2.2%. Using this conservative estimate, and increasing this estimate to 6%, will ensure we meet our production goals and genetic diversity. These variables result in four different potential daily larvae production rates (**S8 Table**).

- - 1. ​Sorting technology impact on mosquito production

The sex sorting technology, used to separate the Cas9 and gRNA pgSIT lines prior to the crossing of the Cas9 males and gRNA females to generate sterile males, can also impact mosquito production. We assessed two commercially available sex sorting technologies that differ in their workflows due to the life stage at which they sort mosquitoes (**S8 Table**). COPAS FP (Union Biometrica, Holliston, MA, USA) sex-sorting strategy relies on the large-particle flow cytometry and enables sex-sorting newly emerged 1st instar (L1) larvae using genetically-encoded sex-specific fluorescent markers. The other technology from Senecio Robotics (Tel Aviv, Israel) applies optical sorting of mosquitoes as newly emerged adults. This sorting timeline creates a substantial difference in the number of larvae reared to adulthood because COPAS does this initial sorting upfront in the production process, while Senecio Robotics requires double the Cas9 and gRNA larvae production since both sexes of larvae need to be raised to adulthood before they are sorted. Therefore, the Senecio Robotics sex sorting method nearly doubles the larval production effort and labor, in addition to being slower at sorting. Another critical point to note is that Verily Life Sciences has its own proprietary sex sorting technology on a comparable scale, but it is commercially unavailable.

- - 1. ​Egg isolation and hatching methodology

Eggs are laid on a moistened surface and then drained from the water post oviposition. The adult mass-rearing cages have a convenient water trough allowing easy access to the water for this process. The eggs are then added to a strain-specific container and left in water overnight (**S3 Fig**). Within two days, larvae are separated from the eggs and sex sorted by COPAS (**S3 Fig**). Alternatively, the mosquitoes are sorted as adults by the Senecio Robotics sorting machine. Optimization of these procedures will be required to improve efficiency, but there are several options, including a straining system that separates sticky eggs and egg fragment pieces, light aversion techniques to move larvae for retrieval, or swirling or other water isolation techniques. Usage of the COPAS for *A. gambiae* specific applications has been well documented, and robust protocols have been established which can be easily scaled [[16]](https://paperpile.com/c/JoQtIv/fzV9K).

- - 1. ​Egg isolation and hatching equipment costs

Egg isolation requires minimal equipment. A simple metal filter can catch the eggs drained from the trough [[15]](https://paperpile.com/c/JoQtIv/JPcz), or the eggs can be laid on biodegradable filter paper. Eggs from colony maintenance intercrosses within the parent Cas9 and gRNA lines will be used to maintain the colony, while sterile male eggs from the Cas9 and gRNA crosses will be transferred to the drone delivery system for distribution. At peak capacity, 70-100 cages will be harvested for eggs daily, 3% of which will support colony maintenance. The IAEA and cage suppliers do not provide the exact volume of water in the trough, but looking at the dimensions provided, we estimate two liters per cage. Therefore, 4.2-6 liters of water will be harvested for colony maintenance, while the other 135.8-194 liters will contain the sterile male pgSIT eggs for processing and field release. These procedures are low cost requiring only plastic buckets, metal sieves, and optionally filter paper to load into drones [[15]](https://paperpile.com/c/JoQtIv/JPcz). Ten-liter buckets cost about 2 USD (Supplier:Taizhou Hengming Plastics & Mould Technology Co., Ltd., Model: HMTY-20L-B) , and 40 buckets would capture all the water before filtering the eggs. Therefore, the startup cost of egg isolation is quite minimal (80 or less USD). Metal sieves would also be inexpensive, with a 38µm sieve costing around 80 USD(Supplier: KimLab , Model: B07P246361). The filter paper may not be required and is a similarly insignificant cost of 1,000 USD per year (84 days of releases multiplied by 10 USD for a pack of large filter paper daily).

### COPAS-related costs

1.3.7a COPAS overview

Sex sorting is a key technology for pgSIT implementation as it is the rate-limiting step in sterile male production. Without high-throughput sex sorting technology, the facility will not be able to achieve production levels required for large scale releases. Additionally, these machines are the most expensive equipment required for the facility. Due to the importance of this technology in the pgSIT production workflow, it is important to build redundancies to account for machine malfunction, repairs, and general maintenance activities.

The COPAS sorting device (Union Biometrica, Holliston, MA, USA) separates larvae based on transgene-linked fluorescent markers. It can detect the loss of the fluorescent markers, which can be used for quality control (indicating unexpected loss of the transgene, or line contamination), or can be used for sex sorting L1 larvae to set up the Cas9 x gRNA line crosses needed to generate sterile males. During the active release phase, daily sex sorting will continually generate more production crosses. During the maintenance phase, the COPAS will periodically screen for the unexpected loss of transgenic markers or contamination. Upon completion of the COPAS sorting, the larvae will be transferred to the rearing racks and trays.

- - 1. b COPAS FP 500 Initial Cost

To maximize egg production, COPAS flow sorting will be run 24 hours a day during the Active Phase, which should meet our production numbers with the minimum number of machines (**S11 Table**). We will also have multiple sets of parts onsite for repairs and a spare machine to maintain

production levels during repairs. Union Biometrica, the producer of these machines, has given us a preliminary quote for unit cost and a contract for repair parts that will provide all common replacement parts. The COPAS FP 500 single laser system with an air compressor for sorting capability will cost 308,174 USD per unit, and the dual laser system will cost 358,898 USD per unit. The dual laser system is desirable for screening multiple fluorescent markers, which is important for the quality control of the parent lines. Union Biometrica offers an annual maintenance fee of 12% of the initial machine cost. This maintenance fee will provide all of the minor and common parts needed to make repairs and remote technical support. They also recommend having spare lasers on site as these are the most expensive components and have a longer lead time than standard parts. The laser costs are included in the annual contract, but a spare onsite laser can avoid long term disruptions in production. Spare lasers cost 46,000 USD each, and it is recommended that we have one spare laser onsite for each type of laser (e.g. two spare lasers are required for the dual laser system).

Each COPAS FP500 can sort up to 1,200,000 larvae daily, yielding approximately 600,000 of the desired genotype if run for 24 hours. Typically, these machines require cleaning between runs, so a more reasonable run time is 23 hours a day for 562,500 larvae per machine daily. To have a release ratio of 32 eggs per wild adult mosquito, the number of larvae that need to be sorted daily to meet peak Active Phase production levels varies from 81,840 to 175,394 larvae (**S8 Table**). If we have efficient fecundity (300 eggs per female), we need to sort 81,840 L1 larvae a day, which requires a fraction of the capability of a COPAS FP 500 machine. Similarly, using the same calculations, if the fecundity of the mosquitoes is more moderate (210 eggs per female) and the survival to adulthood is lower (50%), we will need to sort 175,394 L1 larvae per day, still requiring less than one COPAS FP 500 machine (**S8 Table**). Two COPAS FP 500 sorting machines will be available if a backup is needed. This cost, plus two spare lasers, results in a total cost of 948,840 USD for the low fecundity group (**S8 Table**). These estimates are based on discussions with Union Biometrica, which indicate a sorting rate of 25,000 L1 larvae per hour. During the Active Phase, we plan to maximize production and assume we can sort 575,000 L1 larvae for 23 hours per day. This production level will last the 12 weeks of the Active Phase. There is uncertainty in these production estimates, as these devices have yet to be evaluated or stress-tested at this production rate for that long duration and with that degree of uninterrupted continual use. Testing the production capabilities of the COPAS FP 500 in the early phase field trials will optimize their use and determine their long-term sorting capabilities. Total cost estimates include additional costs that factor in doubling the COPAS FP machines, and this is taken into account for initial and annual costs (**S9, S10, and S11 Tables)**.

Additional costs associated with shipping and importing equipment to The Gambia are estimated to be no more than 25% of the equipment costs by trade organizations. This cost is included in the total upfront costs estimated in the **S11** **Table**. Since this equipment is used for disease prevention, perhaps reduced import tariffs and taxes commonly afforded to medical equipment can be negotiated with the government to minimize these costs, but currently, 25% is our best estimate of these costs [[17]](https://paperpile.com/c/JoQtIv/1dnv).

- - 1. c COPAS FP 500 annual cost

The annual maintenance costs for the COPAS are 12% of the initial machine costs as per our communication with Union Biometrica. This cost includes necessary maintenance supplies and replacement parts. This service fee will allow us to keep most general parts onsite and restock as needed, except for a spare laser, which is factored into the upfront cost. Restocking of the lasers will be included in the service agreement, but to avoid production delays due to sourcing and shipping, our estimates include the cost to keep at least one onsite. The laser fee should be a one time purchase as the maintenance fee will replace lasers and maintain that spare laser availability.

- - 1. a Larvae rearing rack and tray system overview

The IAEA has developed a mass-rearing protocol that we utilized to calculate our pgSIT rearing costs. For larval rearing and feeding, the Wolbaki mass rearing rack and tray system (Guangzhou Wolbaki Biotech Co.,Ltd, Guangzhou, Guangdong Province, P.R.China) is commercially available and can rear approximately 5,000 larvae per tray and contains 100 trays per rack(Supplier: Guangzhou Wolbaki Biotech Co.,Ltd, Model: WBK-P0003-V2). These larvae will be fed daily a food mix that has been shown to be effective for rearing *A. gambiae* larvae comprising three types of commercially available fish food. A cheaper alternative food mix will be discussed later in this paper (**S14 Table**). The feeding schedule is based on the IAEA standard

[[15]](https://paperpile.com/c/JoQtIv/JPcz) but is staggered one day to allow unfed larvae to be sorted by COPAS.

- - 1. b Larvae rearing rack and tray system costs

The Wolbaki mass larvae rearing system priced its rack and tray system at approximately 22,500 USD(per preliminary quote from Guangzhou Wolbaki Biotech Co.,Ltd, 2023) (Supplier: Guangzhou Wolbaki Biotech Co.,Ltd, Model: WBK-P0003-V2). Other companies had higher prices for similar scale systems. Some other competitors likely have cost-effective rearing systems but have not been forthcoming with pricing despite communications over several months. To ensure we had multiple, cost-effective options for larval rearing, we obtained piecemeal cost estimates for individual components that comprise a larval rearing rack and tray system. The thermoplastic trays with similar dimensions sell for about 0.99 USD per unit (Supplier: Yantai Tongli Hotel Equipment & Supplies Co., Ltd., Model: MW-0001). ABS is also a common 3D printed material so if custom tray design is cost prohibitive, additive manufacturing on site for repairs could be achieved for less than 300 USD for the printer along with ABS filament (Supplier: Shenzhen Creality 3D Technology Co, Ltd., Model: Ender 3 V2). Acrylonitrile butadiene styrene (ABS) is a common, durable, inexpensive, food safe thermoplastic plastic. The racks, while a bit more complex, are aluminum framed and hold 50 trays. Similar aluminum racks have been priced at multiple distributors for around 500 USD. These racks, however, are missing a tilting mechanism that allows water and eggs to be easily removed. Hence, production estimates without this feature must consider alternatives for water removal and egg collection. A simple ratcheting device, which could be placed under the wheels, could achieve this. While a more affordable rack and tray system may become available to meet mass rearing demands, for now, we will evaluate the Wolbaki rack and tray system as this is currently commercially available. If lower cost options become available, there will be opportunities to include these options in future facility planning.

Using the 22,500 USD unit price and our known egg production needs (**S12 Table**) we can estimate the required units and their costs. To estimate the necessary racks, we first take the number of larvae reared per day and divide this by the number of larvae a rack can hold. The Wolbaki rack has 100 trays each that can hold 5,290 larvae per tray, so 529,000 larvae per rack. Larvae are in these trays for up to 8 days. To account for breakage and cleaning time, however, sufficient racks and trays for up to 9 days are beneficial. Cleaning can be done manually, or a machine washing system could be purchased. Therefore, the minimum cost to procure the rack and rearing systems is 45,000 USD. COPAS FP 500 sorting has the advantage of sorting L1 larvae rather than sorting mosquitoes at the adult stage, cutting the rearing numbers in half compared to Senecio Robotics. COPAS's USD maximum rack and tray cost assumes the lowest fecundity and survival conditions. Senecio Robotics rack costs would be double that of COPAS FP 500.

Additionally, we factored in a 1% annual maintenance cost for the rack and tray systems. Unlike the maintenance service fees provided from Union Biometrica for the COPAS Sorting technology, maintenance fees for other pieces of equipment are estimated based on a percentage of the replacement asset value (RVA). This is a percentage applied to the full cost of the item based on expected durability annually to account for the need for repairs or replacement of the equipment. For an entire factory, this is typically between 1.8% and 5% of the total costs annually, so we attempt to keep our maintenance estimates close to these values[[18]](https://paperpile.com/c/JoQtIv/PX1x). As there is a lack of data tracking required maintenance costs for these specialized pieces of equipment, we consider the equipment, the components and the most vulnerable pieces and apply a percentage accordingly. The metal rack is the most expensive but sturdy part of the system, so minimal rack repairs are expected with proper use. The trays, on the other hand, while still expected to be durable, are expected to be damaged more frequently but are inexpensive thermoplastic trays that should be replaceable at about 1 USD per tray. Bulk purchase of replacement parts could be procured to have readily available replacements and this may allow for discounts. Additive manufacturing methods on site could also be utilized to further reduce costs. Therefore, we assume a 1% annual maintenance cost to replace broken trays and repair racks (**S12 Table)**.

In the potential range in costs for the rearing system, a competitor technology produced by Vienna Scientific (Alland, Austria) can capture the high end of potential costs, as their larvae rearing racks cost 38,000 USD(Preliminary quote from Vienna Scientific)(Supplier Vienna Scientific, Model: IR-MMR-TR50) . In lieu of a confidence interval estimate, which was not feasible due to limited cost data, we used this value for our high estimate in order to capture the risk of relying on the most expensive option.

- - 1. a Water and larval food overview

Water and larval food requirements depend on the selected larvae rack and tray system. The greatest usage of these resources is during the Active Phase as described earlier in this supplemental text. Still, it is important to account for the Maintenance Phase and Ramping Phase when estimating the usage of these inputs. The Maintenance Phase represents most of the year and is when a minimal colony of the parental lines is maintained. With this in mind, we calculated using one mass rearing rack and tray system every three weeks. The mass rearing rack exceeds project requirements, but we preferred to overestimate the colony size to guarantee that we maintain some genetic diversity. For the purpose of cost estimation, we assume that the Ramping Phase uses the same amount as the Active Phase, even though this is an overestimate. In the Active Phase, production is at maximum capacity and will fill a set number of larval racks per week (**S15 Table**).

The water cost is estimated by the rate of water usage associated with each phase. There are 100 trays per rack, and each rack can hold about 530 liters of water. To calculate the usage in the Ramping and Active Phase, the total amount of water for those racks (total racks x 530 liters) is multiplied by 2 to cover water loss and other activities that require water resources (e.g. cleaning, sugar water, egg laying water, and larval feed mixing) (**S13 Table**). The Maintenance Phase is estimated to require 18,020 liters of water and was calculated by estimating the water usage in 1 rack over 34 weeks and doubling the water usage to account for water loss and other activities (**S13 Table**). These estimates are then added together and described below in the cost section.

The larval feed differs from the IAEA protocol due to the development of a more cost effective food formula for *A. gambiae* [*[19]*](https://paperpile.com/c/JoQtIv/ex1X). This alternative rearing diet selects globally available ingredients that can be affordably sourced in The Gambia (**S14 Table**). There are three ingredients for this cost-effective formula: tuna meal, brewer’s yeast and chickpea flour. Tuna meal is a byproduct from the processing of tuna that is sold in bulk for industrial farming processes typically in aquaculture or animal feeding[[20,21]](https://paperpile.com/c/JoQtIv/kjuu%2Ba5F9) (Supplier: T.C. Union Agrotech, Product: Tuna Meal). Alternative fish meal could be a substitute if tuna meal becomes less competitively priced. Brewer’s yeast is a source of vitamins and other useful nutrients for insect rearing[[19,22]](https://paperpile.com/c/JoQtIv/ex1X%2BM7OD)(Supplier: MP Biomedicals, Model: 0290331280). This ingredient is used as a replacement for bovine liver to reduce costs. It is commonly used for brewing and other industrial activities, so it is expected to be easily procurable and may be sourced locally. Chickpea flour is a cheaper alternative to bovine liver powder [[19]](https://paperpile.com/c/JoQtIv/ex1X) (Supplier: Bulk Foods, Model: 40042). Other alternatives could be explored to enable more resilience with related protein rich flour alternatives. Several of these prices are consumer directed and it is likely that discounts will be available for bulk commercial or industrial purchases. Each tray requires about 1.5 liters of this rehydrated food per week following the feed rate from the IAEA protocol [[15]](https://paperpile.com/c/JoQtIv/JPcz) and the increased size of the Wolbaki Trays. This approach results in about 150 liters of this feed per rack weekly. Using the same logic as the water calculation, the Maintenance Phase food requirement is 5,100 liters (**S15 Table**). The specific food requirements are calculated in the same manner as water usage without doubling for cleaning and other uses.

- - 1. b Water and larval feed annual costs

Water and larval feed costs are essential annual expenditures to maintain production levels. Water costs in West Africa are typically around 0.01 USD per liter [[23]](https://paperpile.com/c/JoQtIv/IvFf). Most pricing data available concerning water is bottled water and potable water for tourists which likely does not reflect the costs for industry in the region. Water is relatively plentiful in the region with the primary difficulty being *E. coli* contamination for potability[[24]](https://paperpile.com/c/JoQtIv/oGxi). A portion of this water may be usable for industrial purposes while being unfit for drinking purposes. Additionally, as further water infrastructure is developed for The Gambia, it is expected that sourcing of water will be affordable and lend some stability to this market. The market costs for the raw material for the larval food costs are approximately 0.0255 USD of dry powder per liter (**S14 Table**). To be consistent with the IAEA protocol, larval feed will often be referenced in volume rather than weight. These estimates can be used to determine the annual cost of larval feed (S15 Table).

Water pricing information for The Gambia and West Africa is limited. Our primary source water pricing data was from the neighboring country of Senegal. With the production facility based in the capital city of Banjul, local water infrastructure at municipal water prices should be suitable, especially as water use is highest during the rainy season. If municipal water is not suitable, it may be prudent to select regions with better water accessibility or partner with groups investing in regional water security.

There is limited market information on the costs of larval feed. Larval feed pricing is broken into the component parts and are not particularly expensive. Due to this being a small fraction of the annual cost, the price fluctuations in these resources are not likely to cause significant changes in costs compared to maintenance fees or salaries for the facility’s workers. Additionally, there are alternative feed formulations that could be utilized or could add substitutions if one of these raw components becomes unavailable or cost restrictive.

- - 1. a Pupae isolation and transfer overview

To isolate pupae, the trays in a rack can be poured into a bucket and then into individual Erlenmeyer flasks to isolate the pupae. Pupae can then be measured by volume using a modified 50 ml conical tube with a fine mesh lid as described in the IAEA protocol [[15]](https://paperpile.com/c/JoQtIv/JPcz). This method can estimate the pupae numbers before addition to the adult cages. There is a trough at the bottom of the cage where water and pupae can be added. The adult cages will also be outfitted with sugar feeders and Hemoteks (**S3 Fig E-F**).

- - 1. b Pupae isolation, transfer, and adult cage costs

Half-liter plastic Erlenmeyer flasks can be purchased at 5 USD per unit (Supplier: Indigo Instruments Model: 55207). Two to three racks will need pupae isolation daily, and one worker will be isolating pupae from two racks a day. We estimate that we will need two Erlenmeyer flasks per worker. These estimates result in the total cost for 4-6 Erlenmeyer Flasks at 20-30 USD, which includes costs for separate flasks for each line.

The estimated number of adult cages needed to support the proposed production ranges from 60 (if female fecundity is high, 300 eggs per female) to 90 adult cages (if female fecundity is lower, 210 eggs per female). Many of the manufacturers charge extra for these cages due to their extra features, but the minimum cost for a unit was estimated by other research groups at 250 USD a unit [[5]](https://paperpile.com/c/JoQtIv/UsiJ). At this price, 60 cages will cost 15,000 USD, and 90 cages will cost 22,500 USD (**S16 Table**). We proposed maintenance cost was 5% as this is likely less durable based on its construction materials(**S16 Table**).

In order to capture the highest expected cage costs, we included a commercial option mentioned in [[5]](https://paperpile.com/c/JoQtIv/UsiJ), which costs approximately 2,500 USD for steel commercial units. This commercial option

is unlikely to be utilized as cheaper options are available, but in an effort to capture the worst expected case scenario, these values are included.

- - 1. a Adult mosquito blood feeding overview

Adult female *A. gambiae* mosquitoes require blood feeding to produce eggs. In the wild, mosquitoes blood feed directly on humans and animals, but within a large-scale facility, this becomes untenable. Fresh blood is the optimal feed for *A. gambiae,* and is affordable when sourced locally (**S17 Table**). Successful feeding of *A. gambiae* with cow blood has been documented [[25]](https://paperpile.com/c/JoQtIv/V4ukT), which we propose as an economically viable blood source. Using artificial feeding devices with drained bovine blood is a sustainable and safer approach to blood feeding than directly on live animals. The daily blood requirement is far below what can be acquired from a slaughtered bovine. Investigation of the stability of the blood may be required investigation if bovine blood cannot be acquired daily during the Active Phase. A common brand of synthetic feeding device, the Hemotek membrane feeding system (Hemotek Ltd, Blackburn, UK), uniformly warms blood or blood substitutes within a membrane and attracts mosquitoes, allowing for life-like feeding without a host. The device will feed the mosquitoes around 60mL of blood with some minor modifications. This approach will require a simple but validated modification to the IAEA protocol to feed mass rearing cages [[15,26]](https://paperpile.com/c/JoQtIv/c0BsT%2BJPcz).

- - 1. b Upfront adult mosquito blood feeding and egg harvesting costs

The main upfront cost for blood feeding is the purchase of Hemotek devices. While there may be opportunities to reduce the costs in the future and use simpler methods, the Hemotek has been vetted for feeding in many settings, and although expensive, it is a conservative approach for our cost estimate. The basic kit for these devices costs 1,181 USD per unit, but a modified unit with an increased blood feeding surface area is required to feed such a large cage properly(Supplier: Hemotek Ltd, Model: SP2W1-1) . This modification increases the typical blood feeding receptacle from 3 mL to approximately 60 mL. This modification could likely be done in-house for a fairly minimal cost as it only requires larger heating plates and a larger plastic container for the blood meal. The facility requires 35-50 of these daily feedings, depending on mosquito fecundity. The total Hemotek equipment costs will be 2,362 to 3,543 USD (**S18 Table**).

Blood will be locally sourced, as the costs of internationally shipping blood are high, and mosquitoes are more optimally reared on fresh blood. There are local slaughterhouses near the planned facility construction site in Banjul. The blood requirement for the facility is about 0.72-1.1 liters per day during ramping or active production. Cattle in The Gambia are primarily *Bos indicus*, Zebu cattle about half the size of standard *Bos taurus* Holstein cattle in the United States. American cattle can have approximately twenty liters of blood extracted when slaughtered, so we estimate that Zebu cattle should have about ten liters each. Local butchers may have to be trained on exsanguination protocols to produce blood sufficiently clean for use in the facility. While there is not a direct cost per liter of blood in The Gambia, it is expected that the local slaughterhouses will charge at or below the US market for bovine blood, which is 2 to 4.5 USD per liter, although bovine blood is not often sold in The Gambia (Supplier: B&R Food Service, Product: “BEEF BLOOD FROZEN 6 GALLON CASE AMERICAN”) [[27]](https://paperpile.com/c/JoQtIv/ScoF) (**S17 Table**). Due to cattle producing ten-fold the daily requirement when slaughtered, exploring refrigeration options for long term storage will be desirable to increase efficiency if cattle are not being slaughtered daily. It is

expected that blood should remain usable for up to five days, but this will have to be confirmed in the context of a mosquito production facility. It is expected to cost less than 100 USD per year to source the blood needed to maintain the colonies (**S17 Table**). Other options have been explored, although this is likely suboptimal when compared to sourcing local blood. The cost of blood from non-local sources increases rapidly due to cold chain transportation requirements or freeze drying plasma, which will also not be as effective for feeding mosquitoes. Developing a local slaughterhouse was also explored, although not discussed as blood requirements are minimal and it is likely there are local businesses which could be collaborated with. In the unlikely case that bovine blood is unavailable, blood alternatives are being explored for *Aedes aegypti* and *Anopheles stephensi,* which may be suitable for *Anopheles gambiae* production [[28–30]](https://paperpile.com/c/JoQtIv/1XTz%2Borch%2BIrnb). As bovine blood is expected to be available and these alternatives are untested, we will not include estimates for blood alternative costs.

These costs are presumed to be the highest expected cost as Hemotek blood feeding devices are not necessary for blood feeding mosquitoes and bovine blood is considered primarily a waste product in Banjul.

- - 1. a Drone delivery of pgSIT eggs to the Upper River region overview

About 8,686,000 eggs need to be flown to the eastern part of the country within 48 hours of laying to ensure the eggs remain viable and fit for release [[31]](https://paperpile.com/c/JoQtIv/NHwjk). To maximize efficacy, eggs will be delivered by drone within 48 hours of harvesting. The predicted dry weight of these eggs is 37.36 grams. However, *Anopheles* eggs cannot be desiccated at any time, so they must be shipped with moisture which will add weight. Drone engineers at Arda (<https://www.ardaimpact.com/>) have advised on drone egg distribution. They concluded that long-distance drone delivery to the eastern parts of The Gambia (the URR and beyond) is feasible from the capital city of Banjul with current technology. The gasoline-powered drones they have can carry a 10 lb payload up to 560 km per trip, which means that this can serve much of the far east of the country from the capital port city of Banjul. This payload capacity allows for significant added water weight for the eggs along with any containers needed for delivery. Delays in egg delivery should also be considered in the cost estimates. While it is optimal for the facility to keep a daily schedule, inclement weather, drone malfunctions, and other issues may cause an interruption in delivery services. Fortunately, *A. gambiae* eggs tolerate cold storage at 4˚C for up to 5 days at high density [[31]](https://paperpile.com/c/JoQtIv/NHwjk). Hatched nascent larvae can survive at ambient temperature for an additional day, potentially expanding the delivery window to three days if necessary. Therefore, there is some flexibility in the delivery schedule.

- - 1. b Costs of drone delivery of pgSIT eggs in the Upper River region, storage costs and delivery considerations

The number of drones required can remain constant as these have a set weight capacity and, therefore, a set egg delivery amount per day. Additional flights can be scheduled to accommodate larger egg distributions. Each drone costs about 25,000 USD as per a quote obtained in 2023, and while these are expected to need minimal maintenance according to the vendor, we are estimating one flight a day with three drones in reserve in case of malfunctions in the primary drones or the need for doubling the delivery rate for a day. This strategy gives us an upfront cost of 100,000

USD for four drones. This estimate has factored in ground support and other features that should allow for autopilot flight and flight monitoring (**S19 Table**). There are multiple companies that could provide similar offerings, but Arda Impact was selected as they have begun collaborations with the government of The Gambia[[32]](https://paperpile.com/c/JoQtIv/A6AL).

The storage of eggs in case of delivery delays also has costs, including a large refrigerator to store the eggs at 4˚C [[31]](https://paperpile.com/c/JoQtIv/NHwjk). Commercial refrigerators cost approximately 1,000 to 3,000 USD, depending on the storage capacity required, but it is likely safe to assume an average cost of 2,000 USD (Supplier: Summit Appliance, Model: ACR45LCAL; Supplier: , Model: FFRF36ADA; Supplier: Bosch, Model: B24CB50ESS). Additionally, a gasoline generator should be procured for the facility to maintain electricity for refrigeration and other technologies in the facility. For a small factory, a 10,000-20,000 USD generator should suffice although selection for maintenance and fuel availability will be key to selection of particular models (Supplier: Generac, Models: RG02515A , XG03245ANAX, RG06045X). The facility should not require storage of more than five days of eggs as their hatch rate declines substantially after that time point [[31]](https://paperpile.com/c/JoQtIv/NHwjk).

1.2.13a Mosquito release in the Upper River region overview

Drones will deliver the eggs to release sites throughout the Upper River region where they will develop from eggs to adults. There are some native habitats where wild mosquitoes lay their eggs, but most are unlikely to support the development of such a large number of mosquitoes. The likely solution will be to engage and hire members of the local communities to support an onsite sterile male mosquito rearing system. This approach would provide an opportunity for community engagement, which correlates with public acceptance and program success [[33]](https://paperpile.com/c/JoQtIv/9Q1CT). These systems would require a large shallow water larval habitat that can be managed to prevent wild mosquitoes from entering and using the habitat.

- - 1. b Mosquito release in the Upper River region costs

Rearing at the distribution site would require several community members per site to supply larval food and maintain water levels in the larval habitat; however, the training required for these activities is minimal. Water can likely be supplied by rainfall or other local water sources. The annual costs will consist of training, worker salaries, and the cost and delivery of feed and water for rearing.

The primary upfront costs include training, networking, and rearing trays. Training costs will involve community outreach efforts to gain community feedback and acceptance of the pgSIT program and to train community members to manage their local mosquito releases. Current outreach programs for mosquito net usage and insecticide residual sprays cost approximately 9,000 USD for The URR(Table S38). As with any new technology, the training and community outreach activities for pgSIT will be more complex than other technologies employed in the region. However, pgSIT may be simpler to implement for community-wide protection, as it only requires compliance and assistance from a few community members. With these operational considerations, we include two outreach and training cost estimates. The lower estimate (18,000

USD) doubles the current 9,000 USD LLIN and IRS outreach budget. A more conservative estimate per Dr. Umberto D’Alessandro is 500,000 USD, which includes a full team of social scientists and community leaders. Both values are accounted for in the **S9 and S10 Table**, respectively. However, we expect the cost will drop significantly after validating pgSIT for large-scale suppression and initial outreach.

Another upfront cost is the mass rearing trays for the release sites in The Upper River region as releasing pgSIT eggs into wild rearing sites may not be optimal. Thermoplastic ABS trays are inexpensive, likely around 1 USD per tray when considering similar designs(Supplier: Yantai Tongli Hotel Equipment & Supplies Co., Ltd., Model: MW-0001). There is the potential to create netted troughs or small ponds for field rearing, but this will require experimentation to determine if this approach is viable. The weekly rearing requirement is linked to the weekly water requirements to rear these mosquitoes, which are calculated from the weekly egg release numbers. The estimated release schedule is just under 61 million eggs per week, only half of which will hatch because pgSIT females die. This release schedule means about 30 million larvae must be reared to adulthood in The URR weekly. Using a similar density of mass rearing, we assume a rearing density of 1 mL per mosquito larva. These field trays or ponds are expected to have sufficient rearing capacity to achieve this goal as the modeling assumes only 26% of eggs survive to adulthood, and larvae at this density are expected to have 50-75% survival to adulthood (**S1 Table**). The 26% survival is derived from the expected survival of eggs, larvae, and pupae in field conditions. Active rearing of pgSIT mosquitoes may increase survival and reduce the total required eggs delivered, but a safer estimate is to use the expected field survival rate. The URR requires 30,000 liters per week to rear the larvae. If we use a tray similar to the Wolbaki trays, we have 5.3 liters per tray, which means we need approximately 5,660 trays for the URR. While there will likely be a range of tray sizes available, and there may be more cost-effective food safe plastic bins, we can still apply this cost to assume the high range cost of 5,660 USD for rearing trays in the URR. Trays could likely be replaced with ponds or troughs with netting lowering this price.

Additional annual costs associated with delivery and rearing in the URR include water, larval feed, and labor. This quantity of water required weekly for the 12-week Active Phases is 359,976 liters. At about 0.01 USD per liter, this costs about 3,600 USD annually (**S20 Table**). The water costs will likely be lower as the release schedule coincides with the rainy season and, therefore, there will be opportunities to capture rainwater. Rain capture procedures could be established at release sites to provide ample water for rearing. Larval feed is calculated using water usage and the 28.3% food per liter of water estimate [[15]](https://paperpile.com/c/JoQtIv/JPcz). The URR would need 8,490 liters of larval feed weekly and about 101,880 liters per year. It will cost around 2,600 USD per year to feed the larval at the release sites in the Upper River and will require transport and distribution (**S20 Table**). The current distribution costs of LLINs in the URR are approximately 60,000 USD annually, and we assume a similar cost for feed distribution (**S20 Table**). The weekly weight of the food required is 84,900 grams and should be delivered as dry food. The annual food requirement is, therefore, 1,020 kilograms for the entire Upper River Region. This amount requires prior delivery to the various release sites to guarantee that they have sufficient feed for the mosquito larvae. Artificial rearing ponds and troughs may reduce larval feed requirements since natural food sources are also available. Pond and trough options are likely viable approaches, but we selected the more

conservative and expensive estimate in case a more expensive approach is required.

The egg releases in the URR will require management on the ground to rear these mosquitoes from an egg to adulthood. Although it is common for anti-malaria programs to utilize volunteer labor that often is not accounted for in budgeting, we wanted to budget these positions as this would provide better incentives for effectively managing these release sites. If these programs become governmentally run, there may be restructuring of local management to volunteer labor. One to two individuals will likely be able to manage mosquito rearing independent of the rearing density and volume. As a rough estimate, we will assume that there will be one rearing site per thousand people, with one local manager, hired part-time to manage mosquito rearing. This estimate may vary depending on human population density and the size of the release site, so this will require further investigation. For the URR, there are about 292,000 people, so we estimate at least 300 field managers are needed for mosquito rearing, or about one manager per thousand people. This job would be part-time, so we took half the monthly salary for the main facility. While the actual work will be significantly less, it would be useful to have field managers with enough time available to troubleshoot any issues that may arise. With a monthly wage of 75 USD and 14 weeks of release (3.5 months), the cost would be 78,750 USD (**S20 Table**). This calculation brings the total cost of approximately 145,000 USD to pay for pgSIT sterile male releases in the URR (**S20 Table**). Water and labor may be discounted if there are cheaper water sources during the rainy season and some volunteer community effort, but this is not guaranteed.

- - 1. ​pgSIT line stability and quality control

To sterilize mosquitoes for the pgSIT system, the Cas9 and gRNA genetic elements need to be stable and effective. In order to estimate the frequency mutations are expected to occur in these key elements, a background mutation rate from closely related *Anopheles* species in the *An. gambiae* complex can be used to estimate the *An.gambiae* mutation rate. Using the spontaneous base substitution rate of 1x10^-9^ per site per generation for *Anopheles coluzzii* [[34]](https://paperpile.com/c/JoQtIv/qkzCN), and the number of bases in the Cas9, gRNAs transgenes and target sites we can determine the likelihood of transgenes of target site mutation. The Cas9 line is inherently more vulnerable to this as it is a larger gene and mutations in most parts of this gene could deactivate this element. The gRNA lines have some resilience as they have inbuilt multiplexed gRNA redundancy (multiple gRNAs for each target gene). A mutation in one of the gRNAs would not result in inactivation of the line. For the gRNA line to be inactivated, all three gRNAs, target sites or some combination would have to be mutated sufficiently to prevent targeting. Not only are these very small sites, gRNAs can tolerate some mutation and the target sites are in critical genes that are unlikely to tolerate mutation. The likelihood of mutations becoming fixed on the allele with the original mutations is also minimal.To calculate the Cas9 mutation frequency, we multiply by the number of mosquitoes per generation to achieve the chance of a deactivating mutation occurring per generation (**S21 Table**). We would therefore expect 17 generations before a mutation would occur in a Cas9 allele. There are 11 generations in the maintenance phase, and its last generation will begin daily, increased egg production. The ramping phase is approximately 5 generations and the active phase 4-6 generations. Even though the Active Phase has daily mosquito generations, they are still on a 3 week cycle, so we would expect 17-18 generations of maintenance annually. For each of these generations, the same maintenance line amount will be utilized to maintain the stock. Based on the

1x10^-9^ per site per generation mutation rate and assuming 18 generations, we would expect about 1.08 mutations annually in the maintained Cas9 stock of mosquitoes. With minimal fitness cost in the Cas9 lines, it is assumed that these mutations will not undergo positive selection. Additionally, the fitness cost between unmutated and mutated may be especially minimal as a Cas9 protein with a single point mutation will likely have similar fitness costs. At this mutation rate, and assuming a neutral fitness cost of the mutation, Cas9 mutations should only affect 1% of the stock population at about 56 years post-production.

Further work is required to more accurately estimate the mutation rate and selection for transgene and target site mutations. However, these estimates can be improved during the earlier field trials. Selecting lines that have minimal fitness cost can also minimize the risk of loss-of-function mutations accumulating in the stock lines. Quality control procedures can also be implemented to identify and remove mutations that reach high enough frequencies to impact pgSIT performance.

Early field and mass production trials can include quality control procedures to evaluate and reduce the release of unedited eggs. The pgSIT release and production plan uses 6% more parental mosquitoes than needed for production, which is three times the maintenance requirement. Some of the extra mosquitoes can be used for quality control efficacy and mutation analysis. Cas9 function and the fidelity of the gRNAs can be evaluated by confirming the sex specificity of the sterile males with the COPAS FP 500 sex sorting procedure. To verify sterility the quality control protocols can include periodic sequencing of the editing sites and a portion of the Cas9 x gRNA offspring can be mated with wildtype females to confirm sterility. These additional quality control procedures should not have a large impact on cost.

- - 1. a Entomological and epidemiological monitoring overview

Mosquito population and malaria incidence monitoring will be necessary to determine if the pgSIT sterile males effectively reduce *A. gambiae* populations and malaria incidence. Two outcomes will determine the success of this intervention: localized suppression of wild *A. gambiae* (entomological impact) and reduction of malaria cases (epidemiological impact) in the treated area. Monitoring mosquito populations requires active efforts to capture/recapture mosquitoes to determine the abundance and composition of the mosquito populations. There are standardized methods for capturing mosquitoes through various trapping methods. Several sites will be selected throughout the region to account for the differences in landscape and relative location to other release sites. Regions near the center of the release coverage area are expected to have better population suppression due to reduced migration to these sites compared to border regions. Facilities and equipment will also be needed to conduct monitoring.

- - 1. b Entomological and epidemiological monitoring costs

The monitoring of the *A. gambiae* population and malaria rates will likely require collaboration with research groups in this region that can lead the studies to monitor the mosquito population

and malaria incidence. To avoid year-to-year variation, we expect to require at least five years of monitoring once the facility is fully operational. The preliminary field trials will provide the data to set a baseline for the entomological studies. The experimental and operational budget should be fairly low because the monitoring will consist of inexpensive insect traps and communication with the existing medical network to determine the respective entomological and epidemiological impacts (**S22 Table**). This approach is the minimum required for monitoring but can be scaled up to meet the project's needs once baseline estimates are obtained from early field studies.

Monitoring cost is dependent on the extent of monitoring. From our conversations with local experts, an extensive monitoring system could require up to 500,000 USD per year. We add this cost to the base value set in the **S22 Table**. However, smaller-scale field trials should inform the scope and costs of monitoring required to assess safety, and entomological and epidemiological impact prior to large-scale release.

- - 1. a Facility construction and supporting systems

The facility requires space to access the racks and cages while providing room to move and clean this equipment. The total space for racks and cages was estimated, and the space was squared to account for additional space required for anterooms, and washing, sorting, maneuvering, and storage spaces required in the facility. By squaring the size estimate for the racks and cages, we aim to overestimate the space requirements for this facility.

- - 1. b Facility construction and supporting systems costs

To estimate the land cost, we averaged the cost of available plots in Banjul on the AccessGambia website and calculated the square meter cost, which was 8.25 USD per square meter at the time **(S23 Table)**. For the size of the plot, we assume 50% more land space than the minimum facility space to have excess space to expand the facility **(S24 Table)**.

This facility should have many of the structural requirements of a light-duty factory. Food processing plants fall into this category, and we confirmed from two sources that the construction costs of a light factory should be approximately 1,600 USD per square meter in West Africa[[35,36]](https://paperpile.com/c/JoQtIv/qXTJ%2BaNc1). With this in mind, the cost of the facility is estimated based on the size required to house the variable number of cages and racks. We also estimated the architectural design cost, up to 14 percent of the facility size, which was used to approximate the architectural design cost, which is above the average expected fees (**S24 Table**). This preliminary estimate is based on limited data on the construction of such a facility in West Africa, so there are some unaccounted for costs associated with this construction.

- - 1. ​Initial facility training

Initial facility establishment requires a team of experts in mosquito mass rearing to plan rearing schedules and operations, supervise the initial day-to-day activities, address issues, and train local staff to troubleshoot problems at the facility. The advisors would work at the facility for up to a

year on-site, possibly broken into two separate work periods spread across two years or for an entire year, with the expectation of committing the maintenance phase to extensively train staff and to support the local team for the initial Ramping Phase, and Active Phases. Some staff may be required earlier to direct the facility purchasing and stocking or to provide logistical support to ensure the site has the resources for the Ramping and Active Phases.

The professional training staff will have several roles in the initial project. They will include scientists with experience in mass rearing insects, preferably *A. gambiae*, logistic managers specializing in operations and developing the requisite systems, and technicians with expertise in sex sorting technology. Salaries were based on average academic salaries at LSHTM. Pay is increased according to the State Department’s hardship differential, which is 20% [[37]](https://paperpile.com/c/JoQtIv/0AOjW).

First, several scientists with mosquito mass rearing and *An. gambiae* species experience should be hired to manage the mosquito racks, cages, egg laying, hatching, and feeding methods. Following colony establishment, a technician or scientist could be hired to train local staff on the basics of mosquito mass rearing and rear the Maintenance Phase of mosquitoes. We recommend three technicians/scientists and one senior scientist to oversee the initial running of the facility. The average pay for these roles was estimated from the LSHTM’s wages, so the adjusted pay scale may be overestimated. The goal for this first year is to ensure the local, permanent staff can acquire the expertise to manage the facility and pgSIT production.

The second group is logistics managers, who can develop efficient supply chains and resource sourcing and manage the complexities of running a large production facility. Ideally, it would be someone working in West Africa who is involved in similar-scale agricultural or light manufacturing work. The range of a logistic manager can range from 80,000 to 150,000 USD depending on experience level. As with the scientist role, if the logistic manager is not local, they will aim to recruit and train local managers to fulfill this role in the long term.

Finally, we require staff with expertise in the selected sex sorting technology. They need to be capable of training local staff and maintaining and addressing any issues with this technology. The average pay for a flow cytometry technologist, a similar technology to COPAS, is approximately 66,000 USD annually. However, the COPAS FP 500 has fewer technicians explicitly trained on this machine available, likely demanding greater pay. If we can only recruit flow cytometry experts, they would have to undergo training on the COPAS before beginning their year in The Gambia, resulting in similar wages. This technology is the backbone of the facility and is a pivotal component for this facility to function. The importance of this technology makes it a priority to ensure good stewardship of the machines.

- - 1. ​Facility annual labor

The initial labor force will be replaced by locals in The Gambia to improve community investment in the project and reduce labor costs. We calculated three potential wage estimates and labor costs spanning the range of expected wages needed to attract labor of sufficient skill [[38]](https://paperpile.com/c/JoQtIv/MVsi)(**S25-S27 Tables**). These salaries were estimated from available data on PayLab, an open source survey based salary collection site, as well as comparing this with the salary information provided by Umberto D’Alesandro. Both of these salary estimates were comparable and were used to provide

a range of estimates as precise salary data in The Gambia was unavailable to us. Labor costs may be further reduced as this work would be primarily seasonal, although maintaining a workforce year round would greatly benefit the facility’s stability and training. This work force could also be utilized to produce mosquito products abroad, or even other pgSIT mosquito species. These costs were estimated by looking at comparable rates from our collaborators in The Gambia (**S25-S27 Tables**).

- - 1. ​Facility costs summarized

Total costs are summarized in the **S28** and **S29 Tables**. The annual costs are summarized in the **S30 Table.** The rearing plan at the URR sites is uncertain so we varied from full cost to no cost USD (**Table 2)**. The total initial costs are summarized in the **S9** and **S10 Tables**. For the post research and development cost, the development cost was subtracted from the total cost estimate. This facility is comparable to previous SIT facilities [[39]](https://paperpile.com/c/JoQtIv/IlXnn).

1. ***​Predicted health benefits of implementing pgSIT in The Upper River Region of The Gambia***

The lives saved and cases prevented were discussed in this paper **(Table 1)** and this information can be used to derive further health estimates useful for economic analysis. In particular, deriving the life years saved and sick days saved is useful for interpreting the direct health benefits of the pgSIT technology. In this model, these epidemiological outcomes are age-stratified, which facilitates calculating the number of life years saved due to the pgSIT intervention. The life years saved can then be used to determine one of the key estimates in this paper, cost per DALY saved **(Table 2)**. The life years saved can be used for other estimates as well and the sick days are used for additional economic analysis in the supplementals.

- 1. ​Life year calculations and life years saved annually

The life years saved are calculated by subtracting the average age of the age bracket from the life expectancy in The Gambia, which is 62.6 years. For the 60 and older category, we assume that those who die of malaria in this category died one year before their time **(S31 Table)**. To calculate the number of life years saved per age group per year **(S32 Table)**, the years saved in each category is then multiplied by the deaths prevented in Table 1.

- 1. ​Sick day calculations and sick days saved annually

The number of sick days prevented is another quantifiable health outcome that can be extrapolated from the modeling data. The incapacitation rate, which divides the number of work days lost by the total number of absentees, is an effective means to estimate the average number of sick days per case of malaria. A study that monitored the economic effects of worker absences due to malaria calculated an average incapacitation rate of 3.7 days [[40]](https://paperpile.com/c/JoQtIv/GA93L). To calculate the number of sick days saved due to the pgSIT intervention, the cases prevented in Table 1 are multiplied by the average sick day rate (**S33 Table**).

Once this intervention has been applied for multiple years, approximately 48,000 sick days are saved annually due to the pgSIT intervention (**S33 Table**). Sick days have a direct economic impact on the community, but it is harder to quantify the indirect effects of sick days, such as the loss of educational opportunities for school-aged children. Notably, sick days in the 0 to 5 years and 5 to 17 years groups are also treated as an economic loss due to the assumption that parents will need to take time off to care for a sick child.

## Quantifying the economic benefits of pgSIT in The Gambia

Five predicted values can be estimated when quantifying the economic benefits of a malaria disease intervention: (3.1) the value of statistical life saved per year, (3.2) value of life per year of life saved, (3.3) estimated GDP growth increase, (3.4) the value of sick days saved, (3.5) medical intervention costs saved, (3.6) current intervention costs reduced, (3.7) value based on willingness-to-pay estimates, and (3.8) additional economic benefits. Many of these values are directly quantifiable.

- 1. *​*Value of statistical life (VSL) saved per year

As shown in the predicted health benefits of implementing pgSIT in The URR of The Gambia, approximately 230 lives would be saved annually (**Table 1**). This number then needs to be applied to the VSL value, which is the local tradeoff between the risk of death and money used to estimate local money that would be paid to prevent a death. Studies evaluate the VSL for many risks, and the USTD uses a selection of these calculations with economic adjustments to estimate the VSL in the United States. For 2022, the United States VSL is estimated to be 12.5 million USD [[41]](https://paperpile.com/c/JoQtIv/Ol8BT). Without a direct estimate of the VSL for The Gambia, we use the US VSL as a benchmark, a practice used frequently in the literature [[42]](https://paperpile.com/c/JoQtIv/QjPj9). Therefore, we used the United States Transportation Department (USTD) VSL and converted the GDP per capita purchasing power parity (PPP) in The Gambia to the United States GDP per capita [[43]](https://paperpile.com/c/JoQtIv/oaB7).

This method of VSL calculation can be used to estimate the VSL in The Gambia by comparing the GDP per capita and adjusting by PPP, a metric to equalize purchasing power between countries. This calculation is done because the per capita GDP is linked to the VSL for a region. GDP per capita adjusted by PPP affects the VSL as the amount able to invest into the cost associated with a reduction in death is limited by budget. Therefore, comparing the United States per capita GDP to The Gambia’s per capita GDP adjusted by PPP can determine the VSL for The Gambia. The Gambia’s GDP per capita is 763 USD (2021)[[44]](https://paperpile.com/c/JoQtIv/dcox). The Gambia’s GDP per capita adjusted by PPP is 2,215 USD, and the United States GDP per capita is 71,055.9 USD[[45]](https://paperpile.com/c/JoQtIv/mTAi). Utilizing these values and the previously shown United States VSL, The Gambia has a VSL of 394,139 USD. With an average of 230 lives saved yearly (**Table 1**), this pgSIT intervention saves approximately 91 million USD in VSL (**S34 Table**). When considering the cost of this program ranges from approximately 315,000 to 318,000 USD annually (**S30 Table**), the annual cost of this program would save lives at about 0.3% of the VSL.

The VSL can change with income. Income elasticity measures the percent change of VSL as income changes by 1%. Based on VSL studies in multiple countries, lower per capita income nations have a higher percentage of their income allocated to cost of living and, therefore, will

have less to spend on extra methods to reduce the risk of death and disease. Thus, VSL increases with income. To capture the variation in how this is quantified, we applied elasticity of 1.0, 1.5 and 2.0 in order to capture the range of potential VSL estimates (**S34 Table)**[[43,46,47]](https://paperpile.com/c/JoQtIv/oaB7%2B8hUA3%2BGBFUA). This approach uses The Gambian PPP adjusted GDP, dividing this by the United States’ GDP, and raising this fraction to a power based on the elasticity (1, 1.5, 2) . The VSLs for The Gambia at elasticities of 1.0, 1.5 and 2.0 will be 394,139, 69,987 and 12,428 USD, respectively. This range is extreme, however, as income elasticities of greater than 1.5 are unlikely. Direct VSL studies in sub-saharan Africa are uncommon and we could not find a study in The Gambia to compare to these estimates. Masterman (2017) suggests that an income elasticity of 1 is most appropriate for assessing most countries outside of the US and thus the age group and total estimates are appropriate at an elasticity of 1 [[43]](https://paperpile.com/c/JoQtIv/oaB7). Fortunately, even with the most extreme income elasticity, this intervention would still be cost-effective if calculating the cost of VSL saved per investment **(S34 Table)**.

- 1. *​*Value of life years based on quality adjusted life years (QALY) saved annually

In addition to calculating the economic trade off expected to save life in the URR saved via the VSL, other estimates calculate this per year of life saved. The average value for the loss of a quality adjusted life year (QALY) in the United States is 104,000 USD [[48]](https://paperpile.com/c/JoQtIv/PpWqQ). This value is calculated in an assortment of ways, but at the core, it is an estimate of the payment willingly made to increase life expectancy by one QALY. This estimate is primarily based on insurance rates, but other methods have been explored. This method of life measurement has been primarily used in the United States and Europe, but a similar approximation to VSL could be used for QALY to take into account the investment that would be made in The Gambia to gain one QALY. Again factoring in GDP differences and the PPP, as seen in the VSL section, the same equation can be used to get a QALY value of 3,280 USD. The QALY values can then be multiplied by the life years saved annually to show the value of life saved using the QALY metric (**S35 Table**).

This QALY estimate, while significantly less than the VSL estimate, will still pay off the investment in the first year of intervention. This variation is likely due to these methods differing in their means of calculating the value of human life. In the following years, the intervention will cost less than 0.8% of the average QALY saved annually (**S35 Table**).

- 1. *​*GDP growth benefit associated with malaria prevention

The effect of malaria on the growth and development of countries is commonly understood to be one of the primary sources of poverty in Africa and in other tropical regions affected by the disease [[49]](https://paperpile.com/c/JoQtIv/5Wmw2). Malaria has an estimated annual reduction in the growth of GDP by 1.3% [[50]](https://paperpile.com/c/JoQtIv/nZg5v). Additionally, there are examples where only a 10% reduction in malaria results in a 0.3% increase in GDP [[50]](https://paperpile.com/c/JoQtIv/nZg5v). This 10% reduction is convenient as the target region of The URR is 11.1% of the population, meaning that reducing malaria by more than 10% in this region would, therefore, result in at least a 0.3% increase in GDP. Accounting for growth benefits in this way is unique compared to other

methods in that it compounds annually upon deployment and results in exponential growth and benefits from this intervention. This intervention will likely be released no earlier than by 2030 as several field trials require completion before wide scale implementation. Utilizing the current 10-year average GDP growth rate of 3.5%, we predict the expected growth rate until 2030. The GDP without intervention and GDP with intervention then diverge, with the column without intervention maintaining the predicted GDP rate and the GDP with intervention a 3.8% growth rate (**S36 Table**). The annual effect of intervention is then the difference in GDP between the predictions with and without intervention. It is expected that this intervention will have an exponential effect on the economic growth of the country (**S36 Table**).

This increase in GDP may be underestimated if this system is leveraged to suppress malaria throughout the region. Without the concern of malaria infection in the URR, it is foreseeable that outside investment into this region would be considerable.

Using the growth benefit of GDP alone, the economic benefits of this intervention pay off the investment in only two years. The valuation of these methods likely has some, but not complete, overlap making the addition of these values overestimates or incomplete estimates of the benefits.

- 1. *​*Value of sick days saved per year

In addition to the VSL, there are approximately 48,000 sick days saved annually (**S33 Table**). This savings is due to preventing nearly 13,000 cases per year in the URR (**Table 1**). There are multiple ways to estimate the value per sick day, but a common approach is taking the QALY and applying this daily. We have shown this with previous estimates converted to The Gambia values. This can be converted to a daily value of 12.96 USD and then be used to calculate the total QALY saved per year by preventing malaria infections. On average, 620,000 USD is saved in QALY annually from the pgSIT intervention (**S35 Table**). While the main value saved is the VSL, it is notable that this value saved will have a more immediate impact on the economy by saving these sick days in that year, whereas the VSL value is spread across many years (**S37 Table**). The value of the sick days saved is nearly double that of the annual cost to run the pgSIT program (**S30 Table**).

- 1. *​*Value of medical intervention saved per year

There are also medical intervention costs saved by malaria case prevention. Potentially all of these medical costs can be reduced if pgSIT eliminates malaria in the region, but this is uncertain until large-scale field testing is completed. The immediate cost that should be reduced is the estimated medical intervention expenditure. For the URR, it is estimated that approximately 28,000 USD is spent on medical interventions to treat malaria (**S38 Table**). This approximation is likely an underestimate as this only accounts for governmental investment in treatment and does not include individual costs. This cost for medical intervention would likely scale with malaria cases, but even assuming this cost remains static, there is the additional cost paid by patients seeking treatment. On average, each patient pays an average of 4.55 USD for treatment in addition to lost wages and the cost of medication (**S39 Table**) [[51]](https://paperpile.com/c/JoQtIv/sivAB). This result leads to approximately 59,000 USD being saved annually (**S40 Table**). All of the medical intervention or associated costs will be immediately saved if the pgSIT intervention is as effective as predicted, as these medical interventions will not be required.

- 1. *​*Value of IRS and LLINs saved per year

The current cost of malaria interventions for the URR of The Gambia is approximately 373,000 USD when excluding the medical intervention costs (**S33 Tables**). This cost will be reduced if pgSIT alone is sufficient for mosquito suppression in The Upper River Region. The model predicts the local extinction of the mosquito population. Therefore, interventions currently being funded, including long lasting LLINs and IRS, may not be necessary in the future. With this in mind, the current intervention budget could eventually be budgeted to fund pgSIT, potentially covering 100% of the facility’s annual cost. With this budgeting, there would not be a significant increase in cost to the long term financing of mosquito suppression in the region.

- 1. *​*Willingness-to-pay value per year

“Willingness-to-pay”(WTP) is a commonly cited means to determine the amount that a person is willing to pay to avoid acquiring a disease. This measure captures the risks of the disease, such as the risk of pain and suffering, the inability to work, disability, and death. Parsing these risks in the WTP metric is difficult, but we can estimate how much the populace would be willing to pay annually to fund a pgSIT system. The average WTP metric for malaria prevention and treatment using current methods is 15.13 in 2022 USD [[52]](https://paperpile.com/c/JoQtIv/Ew7aN). As current methods are unable to achieve malaria elimination in many regions, it is foreseeable that the WTP could be increased for this intervention. Using the current population of the URR and applying the expected population growth rate, the annual WTP for malaria prevention can be predicted (**S41 Table**). There are similar studies that look at The Gambia population's willingness to pay for a national health insurance system [[53]](https://paperpile.com/c/JoQtIv/ikf0o). This study includes all medical interventions and puts the WTP at 23.27 USD on average, which corroborates the WTP for malaria interventions as it is a significant fraction of the total medical intervention cost.

The WTP estimate could also be used as a rough approximation of the value assigned by the local populace to malaria prevention interventions or as an individual cost contribution estimate for pgSIT if the citizens of the URR contributed financially to the facility as an alternative malaria intervention. This calculation shows that this project is priced to support interest from the local population (**S41 Table**). It should be noted that this WTP is based on the willingness to pay for LLIN, IRS interventions, and treatments that will reduce the individual risk of infection and treat the disease. It is foreseeable with this intervention being more effective that the WTP for GE sterile mosquitoes would be greater than the WTP we are using. Importantly, the WTP should not be expected to fund the development of this project. The WTP money is an estimate of what the affected population would pay to prevent malaria with current methods and may not be readily taxable for The Gambia to fund this effort. This WTP price suggests that the local populace would be willing to be taxed up to 15.13 USD to prevent malaria, which could indefinitely support a government funded program. The annual cost of maintaining and running the facility is 5-8% of the WTP value, which is a small fraction of that value. This percentage will consistently decrease as the population of the URR increases, and more people invest in this intervention, making the total Upper River WTP increase. These local contributions facilitate the local sustainability of the

project and would, therefore, along with any export income, limit the need for indefinite funding from external funding sources. Eventually, the project may be transferred to the local government, which would be responsible for providing the funds to support the intervention. The WTP value indicated that local financial support is likely in the long term making this project sustainable economically in the URR of The Gambia.

- 1. *​*Additional economic benefits

There are additional less quantifiable economic benefits of implementing pgSIT in the URR. Building the facility within The Gambia will have economic and security benefits for the country. All of the WHO approved LLINs and IRS insecticides are produced outside of The Gambia. LLINs are all produced in East Asia, Europe, and Tanzania, and all IRS chemicals are produced outside of Africa except for one facility in South Africa [[54]](https://paperpile.com/c/JoQtIv/taCH4). The pgSIT approach provides an additional financial benefit to The Gambia by providing investment, development, and financial input into the local economy that would typically be directed to foreign countries. There would be an initial investment in the necessary infrastructure as well as annual investment of 60,000 USD to the economy and workforce of The Gambia annually. In Tanzania, a similar benefit was seen when building a local factory to produce LLINs [[55]](https://paperpile.com/c/JoQtIv/VTtvL). There are also additional security and stability benefits. Utilizing these facilities to produce pgSIT for export can supplement the income of this facility.

#### Current Intervention Costs Locally and Cost per DALY Averted of Current Interventions

- 1. *​*Current local intervention costs

The Gambia currently uses standard practices for malaria prevention and their annual costs have been calculated from communication with various medical and aid organizations in The Gambia **(S42 Table)**. The total annual costs of the nationwide malaria prevention programs can be used to estimate the costs for the target region of the URR **(S38 Table)**. Data collection was conducted from December 5th to 9th, 2022 using structured questionnaires to estimate the values of the resources used for the prevention of malaria. The questionnaires, which were based on published costing surveys, were adapted to data collection relative to the Gambian context. The data were extracted by reviewing programmatic documents, microplanning, budget, and financial reports basing on actual expenditures and economic costs borne by institutions that supported the implementation of the prevention measures against malaria, including national malaria control program (NMCP), WHO, and partnering institutions. The cost interventions included indoor residual spraying, intermittent preventive treatment, seasonal malaria chemoprevention, and long lasting insecticide nets. Only the financial costs associated with the implementation of the mentioned interventions were collected. Costs collected included supervision, meetings, social mobilization, training, salaries and per diems, material and equipment, fuel, lubricant, maintenance, transportation, rental, catering, operating costs, other recurrent program costs as well as overall program start-up costs.

- 1. *​*Comparing Current Interventions to pgSIT Estimates

While pgSIT does not have a peer competitor technology for malaria elimination, there is an interest to compare pgSIT to current malaria interventions. It is also important to note that the benefits of current interventions and the predicted benefits of pgSIT significantly outweigh the costs when considering them as part of a Benefit Cost Analysis[[46]](https://paperpile.com/c/JoQtIv/8hUA3). To compare current interventions to pgSIT, we have selected to compare the cost effectiveness in metrics of cost per case/DALY/death averted and cost per person covered. However, current interventions cannot be directly compared to pgSIT until pgSIT is comprehensively evaluated in long-term studies. In the short term, pgSIT will be used in conjunction with current interventions. We focused on preventative vector management strategies, such as LLINs and IRS, as these are more comparable to pgSIT, which prevents malaria by targeting the vector. We used Conteh et al. (2019), which reviewed 103 primary cost studies and derived costs per DALY, death, and case averted as well as cost per person protected for current interventions. These estimates were done in 2018 USD and were inflated to 2022 USD to be comparable to our costs estimates **(S43 Table)**. These estimates are generally comparable to the pgSIT annual costs and the annual production costs. As current interventions do not include research and development costs and facility construction costs, these are the best estimates to compare to current interventions. Only the ITN cost per case prevented was notably different at a little over 50% cheaper than pgSIT. This may be due to the fact that this paper focused on ITN/LLIN campaigns to protect children and pregnant women, populations particularly vulnerable to malaria. Treating these groups is likely highly cost effective and may not be a fair comparison to pgSIT as our estimates are for the entire population.

**Supplementary Figures:**

#### S1 Fig. Seasonal rainfall profile for Upper River region, The Gambia.

Points represent mean daily rainfall measurements (in mm) for the three years between January 1st, 2017 and December 31st, 2019. The solid line represents the seasonal rainfall profile, fitted using the umbrella package in R (<https://github.com/mrc-ide/umbrella>). This is used to calculate the time-varying environmental carry capacity for larvae in the life history module of MGDrivE 3.

#### S2 Fig. Phased testing pathway for genetically modified mosquitoes.

This figure was based on guidelines and a figure by WHO [[56]](https://paperpile.com/c/JoQtIv/fhrvj). Figure generated in BioRender.com.

#### S3 Fig. Mass rearing during the facility’s active phase.

The general process of mass rearing *Anopheles gambiae* mosquitoes when the facility is actively producing mosquitoes for release is shown. Generating pgSIT sterile males (**Factory Stages)**- This begins with hatching the Cas9 and gRNA parent lines (A-B). Assuming that COPAS is used, sex sorting occurs at the L1 larval stage by sex-specific fluorescent markers (C). If Senecio Robotics (or the Verily method) is used, sorting occurs at early adult emergence (pupal isolation and adult cage D-E). Following the crossing of these lines, offspring larvae are mass-reared in trays for seven to nine days. On days seven to nine, pupae are isolated from the trays and transferred to adult-rearing cages (or to a screening cage for the Senecio Robotics technology sex sorting approach) (D-E). Males from the Cas9 line and females from the gRNA line will mate *ad libitum* and acclimate for three days (E). Mosquitoes are then blood-fed by an artificial Hemotek feeder or by a similar method (Section 2.1.3.9) (F). Two days post blood feeding, water is added to the cage trough for egg laying. The following day, the eggs are harvested and distributed to the field (G). The pure-bred lines are used to create the next generation of the parental line, and this repeats the cycle at the facility (G). Maintenance and Ramping Phases have the same Factory Stages and do not have Release Stages. (**Release Stages**)- The egg delivery to the release sites will be done by drone or other vehicles (H). Once distributed in the field, the larvae will be raised in shallow trays to adulthood, when they mate with wild female mosquitoes (I). This Active Phase production is continued for 12 weeks whereby modeling predicts localized extinction of *A. gambiae* (J). Figure generated in BioRender.com.

Literature Cited in the Supplemental Methods

1. [Smidler AL, Apte RA, Pai JJ, Chow ML, Chen S, Mondal A, et al. Eliminating Malaria Vectors with Precision Guided Sterile Males. bioRxiv.](http://paperpile.com/b/JoQtIv/ZftFg) [2023. doi:](http://paperpile.com/b/JoQtIv/ZftFg)[10.1101/2023.07.20.549947](http://dx.doi.org/10.1101/2023.07.20.549947)
2. [Weng S-C, Antoshechkin I, Marois E, Akbari OS. Efficient Sex Separation by Exploiting Differential Alternative Splicing of a Dominant](http://paperpile.com/b/JoQtIv/j4ptL) [Marker in Aedes aegypti. bioRxiv. 2023. p. 2023.06.16.545348. doi:](http://paperpile.com/b/JoQtIv/j4ptL)[10.1101/2023.06.16.545348](http://dx.doi.org/10.1101/2023.06.16.545348)
3. [Long KC, Alphey L, Annas GJ, Bloss CS, Campbell KJ, Champer J, et al. Core commitments for field trials of gene drive organisms.](http://paperpile.com/b/JoQtIv/4UxDP) [Science. 2020;370: 1417–1419.](http://paperpile.com/b/JoQtIv/4UxDP)
4. [Schairer CE, Najera J, James AA, Akbari OS, Bloss CS. Oxitec and MosquitoMate in the United States: lessons for the future of gene drive](http://paperpile.com/b/JoQtIv/BI07S) [mosquito control. Pathog Glob Health. 2021;115: 365–376.](http://paperpile.com/b/JoQtIv/BI07S)
5. [Maïga H, Mamai W, Bimbilé Somda NS, Konczal A, Wallner T, Herranz GS, et al. Reducing the cost and assessing the performance of a](http://paperpile.com/b/JoQtIv/UsiJ) [novel adult mass-rearing cage for the dengue, chikungunya, yellow fever and Zika vector, Aedes aegypti (Linnaeus). PLoS Negl Trop Dis.](http://paperpile.com/b/JoQtIv/UsiJ) [2019;13: e0007775.](http://paperpile.com/b/JoQtIv/UsiJ)
6. [Dabira ED, Soumare HM, Conteh B, Ceesay F, Ndiath MO, Bradley J, et al. Mass drug administration of ivermectin and](http://paperpile.com/b/JoQtIv/rcYb) [dihydroartemisinin-piperaquine against malaria in settings with high coverage of standard control interventions: a cluster-randomised](http://paperpile.com/b/JoQtIv/rcYb) [controlled trial in The Gambia. Lancet Infect Dis. 2022;22: 519–528.](http://paperpile.com/b/JoQtIv/rcYb)
7. [ClinicalTrials.gov. [cited 13 Nov 2024]. Available:](http://paperpile.com/b/JoQtIv/Lltc) <https://clinicaltrials.gov/study/NCT03576313>
8. [Guidelines for standardised mass rearing of Anopheles mosquitoes - Version 1.0. 21 Dec 2017 [cited 6 Aug 2024]. Available:](http://paperpile.com/b/JoQtIv/VU3fE) <https://www.iaea.org/resources/manual/guidelines-for-standardised-mass-rearing-of-anopheles-mosquitoes-version-10>
9. [Weng S-C, Antoshechkin I, Marois E, Akbari OS. Efficient sex separation by exploiting differential alternative splicing of a dominant marker](http://paperpile.com/b/JoQtIv/ltnjc) [in Aedes aegypti. PLoS Genet. 2023;19: e1011065.](http://paperpile.com/b/JoQtIv/ltnjc)
10. [Gambia Bureau of Statistics. Population and Housing Census of Gambia, 2013. In: Gambia Data Portal [Internet]. 1 Jun 2015 [cited 2 Jun](http://paperpile.com/b/JoQtIv/ZF5D5) [2023]. Available:](http://paperpile.com/b/JoQtIv/ZF5D5) <https://gambia.opendataforafrica.org/mmfoqkd/population-and-housing-census-of-gambia-2013>
11. [Clements AN. The Biology of Mosquitoes. CABI; 1992.](http://paperpile.com/b/JoQtIv/x3SMC)
12. [Yaro AS, Dao A, Adamou A, Crawford JE, Traoré SF, Touré AM, et al. Reproductive Output of Female Anopheles gambiae (Diptera:](http://paperpile.com/b/JoQtIv/GYbvj) [Culicidae): Comparison of Molecular Forms. J Med Entomol. 2006;43: 833–839.](http://paperpile.com/b/JoQtIv/GYbvj)
13. [Agyapong J, Chabi J, Ablorde A, Kartey WD, Osei JHN, de Souza DK, et al. Ovipositional Behavior of Anopheles gambiae Mosquitoes.](http://paperpile.com/b/JoQtIv/jJwH0) [Trop Med Health. 2014;42: 187–190.](http://paperpile.com/b/JoQtIv/jJwH0)
14. [Yaro AS, Dao A, Adamou A, Crawford JE, Ribeiro JMC, Gwadz R, et al. The distribution of hatching time in Anopheles gambiae. Malar J.](http://paperpile.com/b/JoQtIv/5dAR3) [2006;5: 19.](http://paperpile.com/b/JoQtIv/5dAR3)
15. [FAO/IAEA. Guidelines for Standardised Mass Rearing of Anopheles Mosquitoes—Version 1.0. FAO Rome, Italy; 2017. Available:](http://paperpile.com/b/JoQtIv/JPcz) <https://www.iaea.org/resources/manual/guidelines-for-standardised-mass-rearing-of-anopheles-mosquitoes-version-10>
16. [Marois E, Scali C, Soichot J, Kappler C, Levashina EA, Catteruccia F. High-throughput sorting of mosquito larvae for laboratory studies and](http://paperpile.com/b/JoQtIv/fzV9K) [for future vector control interventions. Malaria Journal. 2012. doi:](http://paperpile.com/b/JoQtIv/fzV9K)[10.1186/1475-2875-11-302](http://dx.doi.org/10.1186/1475-2875-11-302)
17. [Framework PS. Gambia Country Commercial Guide: Gambia - Distribution & Sales Channels. [cited 29 Jun 2023]. Available:](http://paperpile.com/b/JoQtIv/1dnv) <https://www.privacyshield.gov/article?id=Gambia-Distribution-Sales-Channels>
18. [Thomas D, Weiss B. Maintenance costs and advanced maintenance techniques in manufacturing machinery: Survey and analysis. Int J Progn](http://paperpile.com/b/JoQtIv/PX1x) [Health Manag. 2021;12. doi:](http://paperpile.com/b/JoQtIv/PX1x)[10.36001/ijphm.2021.v12i1.2883](http://dx.doi.org/10.36001/ijphm.2021.v12i1.2883)
19. [Bimbilé Somda NS, Dabiré KR, Maiga H, Yamada H, Mamai W, Gnankiné O, et al. Cost-effective larval diet mixtures for mass rearing of](http://paperpile.com/b/JoQtIv/ex1X) [Anopheles arabiensis Patton (Diptera: Culicidae). Parasit Vectors. 2017;10: 619.](http://paperpile.com/b/JoQtIv/ex1X)
20. [Kim K-D, Jang JW, Kim K-W, Lee B-J, Hur SW, Han H-S. Tuna by-product meal as a dietary protein source replacing fishmeal in juvenile](http://paperpile.com/b/JoQtIv/kjuu) [Korean rockfish Sebastes schlegeli. Fish Aquatic Sci. 2018;21. doi:](http://paperpile.com/b/JoQtIv/kjuu)[10.1186/s41240-018-0107-y](http://dx.doi.org/10.1186/s41240-018-0107-y)
21. [T.C.UNIONAGROTECH Co.,Ltd Tuna Meal, Fish Meal, ผลิตและจําหน่ายวัตถุดิบและอาหารสัตว์. [cited 13 Nov 2024]. Available:](http://paperpile.com/b/JoQtIv/a5F9) <https://www.tcunionagrotech.com/#products>
22. [Brewers Yeast. [cited 13 Nov 2024]. Available:](http://paperpile.com/b/JoQtIv/M7OD) <https://www.mpbio.com/us/brewers-yeast>
23. [AAAWater. Senegal. In: AAAWater [Internet]. 4 Apr 2019 [cited 3 Jun 2023]. Available:](http://paperpile.com/b/JoQtIv/IvFf) <https://aaawater.net/nproject/senegal/>
24. [The Gambia’s Water Paradox. In: Center for Collaborative Investigative Journalism [Internet]. 4 Jun 2021 [cited 13 Nov 2024]. Available:](http://paperpile.com/b/JoQtIv/oGxi) <https://ccij.io/article/water-paradox-in-the-gambia/>
25. [Lyimo IN, Keegan SP, Ranford-Cartwright LC, Ferguson HM. The impact of uniform and mixed species blood meals on the fitness of the](http://paperpile.com/b/JoQtIv/V4ukT) [mosquito vector Anopheles gambiae s.s: does a specialist pay for diversifying its host species diet? J Evol Biol. 2012;25: 452–460.](http://paperpile.com/b/JoQtIv/V4ukT)
26. [Damiens D, Soliban SM, Balestrino F, Alsir R, Vreysen MJB, Gilles JRL. Different blood and sugar feeding regimes affect the productivity of](http://paperpile.com/b/JoQtIv/c0BsT)

[Anopheles arabiensis colonies (Diptera: Culicidae). J Med Entomol. 2013;50: 336–343.](http://paperpile.com/b/JoQtIv/c0BsT)

1. [B&R Food Services. Beef blood frozen 6 gallon case American. [cited 8 Jun 2023]. Available:](http://paperpile.com/b/JoQtIv/ScoF) <https://www.brfood.us/product/beef-blood-frozen-6-gallon-case-american.html>
2. [Gonzales-Wartz KK, Sá JM, Lee K, Gebremicale Y, Deng B, Long CA, et al. Infectivity of Plasmodium parasites to Aedes aegypti and](http://paperpile.com/b/JoQtIv/1XTz) [Anopheles stephensi mosquitoes maintained on blood-free meals of SkitoSnack. Parasit Vectors. 2024;17: 290.](http://paperpile.com/b/JoQtIv/1XTz)
3. [Kandel Y, Mitra S, Jimenez X, Rodriguez SD, Romero A, Blakely BN, et al. Long-Term Mosquito culture with SkitoSnack, an artificial blood](http://paperpile.com/b/JoQtIv/orch) [meal replacement. PLoS Negl Trop Dis. 2020;14: e0008591.](http://paperpile.com/b/JoQtIv/orch)
4. [Gonzales KK, Rodriguez SD, Chung H-N, Kowalski M, Vulcan J, Moore EL, et al. The effect of SkitoSnack, an artificial blood meal](http://paperpile.com/b/JoQtIv/Irnb) [replacement, on Aedes aegypti life history traits and gut Microbiota. Sci Rep. 2018;8: 11023.](http://paperpile.com/b/JoQtIv/Irnb)
5. [Mazigo E, Kidima W, Myamba J, Kweka EJ. The impact of Anopheles gambiae egg storage for mass rearing and production success. Malar J.](http://paperpile.com/b/JoQtIv/NHwjk) [2019;18: 52.](http://paperpile.com/b/JoQtIv/NHwjk)
6. [Cuenca O. The Gambia to explore medical drone logistics. In: Voyageur Group [Internet]. 22 May 2023 [cited 13 Nov 2024]. Available:](http://paperpile.com/b/JoQtIv/A6AL) <https://www.airmedandrescue.com/latest/news/gambia-explore-medical-drone-logistics>
7. [Diepeveen S, Ling T, Suhrcke M, Roland M, Marteau TM. Public acceptability of government intervention to change health-related](http://paperpile.com/b/JoQtIv/9Q1CT) [behaviours: a systematic review and narrative synthesis. BMC Public Health. 2013;13: 756.](http://paperpile.com/b/JoQtIv/9Q1CT)
8. [Rashid I, Campos M, Collier T, Crepeau M, Weakley A, Gripkey H, et al. Spontaneous mutation rate estimates for the principal malaria](http://paperpile.com/b/JoQtIv/qkzCN) [vectors Anopheles coluzzii and Anopheles stephensi. Sci Rep. 2022;12: 226.](http://paperpile.com/b/JoQtIv/qkzCN)
9. [Gerhard Brümmer, Lucy McLane, Elisa Campos, Thelma Hlatshwayo. 2022/23 Property & Construction Africa Cost Guide Handbook.](http://paperpile.com/b/JoQtIv/qXTJ) [AECOM.com; 2022.](http://paperpile.com/b/JoQtIv/qXTJ)
10. [The Cost of Building in Africa. In: RLB | Americas [Internet]. Rider Levett Bucknall; 9 Apr 2022 [cited 31 May 2023]. Available:](http://paperpile.com/b/JoQtIv/aNc1) <https://www.rlb.com/americas/insight/perspective-2022-vol-1/the-cost-of-building-in-africa/>
11. [U.S. department of state. [cited 6 Jun 2023]. Available:](http://paperpile.com/b/JoQtIv/0AOjW) <https://aoprals.state.gov/web920/hardship.asp>
12. [Gendron W, Raban R, Mondal A, Sánchez C HM, Smidler A, Zilberman D, et al. Cost-effectiveness of Precision Guided SIT for Control of](http://paperpile.com/b/JoQtIv/MVsi) [Anopheles gambiae in the Upper River Region, The Gambia. bioRxiv. 2023. p. 2023.07.20.549762. doi:](http://paperpile.com/b/JoQtIv/MVsi)[10.1101/2023.07.20.549762](http://dx.doi.org/10.1101/2023.07.20.549762)
13. [Brown ZS, Jones MS, Mumford J. Economic principles and concepts in area-wide genetic pest management. The economics of integrated](http://paperpile.com/b/JoQtIv/IlXnn)

[pest management of insects. UK: CABI; 2019. pp. 96–121.](http://paperpile.com/b/JoQtIv/IlXnn)

1. [Lukwa AT, Mawoyo R, Zablon KN, Siya A, Alaba O. Effect of malaria on productivity in a workplace: the case of a banana plantation in](http://paperpile.com/b/JoQtIv/GA93L) [Zimbabwe. Malar J. 2019;18: 390.](http://paperpile.com/b/JoQtIv/GA93L)
2. [Departmental guidance on valuation of a statistical life in economic analysis. [cited 12 Jan 2024]. Available:](http://paperpile.com/b/JoQtIv/Ol8BT) [https://www.transportation.gov/office-policy/transportation-policy/revised-departmental-guidance-on-valuation-of-a-statistical-life-in-econom](https://www.transportation.gov/office-policy/transportation-policy/revised-departmental-guidance-on-valuation-of-a-statistical-life-in-economic-analysis) [ic-analysis](https://www.transportation.gov/office-policy/transportation-policy/revised-departmental-guidance-on-valuation-of-a-statistical-life-in-economic-analysis)
3. [Viscusi WK. Pricing the global health risks of the COVID-19 pandemic. J Risk Uncertain. 2020;61: 101–128.](http://paperpile.com/b/JoQtIv/QjPj9)
4. [Kip Viscusi W, Masterman CJ. Income Elasticities and Global Values of a Statistical Life. Journal of Benefit-Cost Analysis. 2017;8: 226–250.](http://paperpile.com/b/JoQtIv/oaB7)
5. [GDP per capita (current US$) - Gambia, The. In: World Bank Open Data [Internet]. [cited 13 Nov 2024]. Available:](http://paperpile.com/b/JoQtIv/dcox) <https://data.worldbank.org/indicator/NY.GDP.PCAP.CD?locations=GM>
6. [GDP per capita (current US$) - United States. In: World Bank Open Data [Internet]. [cited 13 Nov 2024]. Available:](http://paperpile.com/b/JoQtIv/mTAi) <https://data.worldbank.org/indicator/NY.GDP.PCAP.CD?locations=US>
7. [Chang AY, Horton S, Jamison DT. Benefit-Cost Analysis in Disease Control Priorities, Third Edition. The International Bank for](http://paperpile.com/b/JoQtIv/8hUA3) [Reconstruction and Development / The World Bank; 2017.](http://paperpile.com/b/JoQtIv/8hUA3)
8. [Hammitt JK, Robinson LA. The Income Elasticity of the Value per Statistical Life: Transferring Estimates between High and Low Income](http://paperpile.com/b/JoQtIv/GBFUA) [Populations. Journal of Benefit-Cost Analysis. 2011;2: 1–29.](http://paperpile.com/b/JoQtIv/GBFUA)
9. [Vanness DJ, Lomas J, Ahn H. A Health Opportunity Cost Threshold for Cost-Effectiveness Analysis in the United States. Ann Intern Med.](http://paperpile.com/b/JoQtIv/PpWqQ) [2021;174: 25–32.](http://paperpile.com/b/JoQtIv/PpWqQ)
10. [Sachs J, Malaney P. The economic and social burden of malaria. Nature. 2002;415: 680–685.](http://paperpile.com/b/JoQtIv/5Wmw2)
11. [Gallup JL, Sachs JD. The economic burden of malaria. Am J Trop Med Hyg. 2001;64: 85–96.](http://paperpile.com/b/JoQtIv/nZg5v)
12. [Broekhuizen H, Fehr A, Nieto-Sanchez C, Muela J, Peeters-Grietens K, Smekens T, et al. Costs and barriers faced by households seeking](http://paperpile.com/b/JoQtIv/sivAB) [malaria treatment in the Upper River Region, The Gambia. Malar J. 2021;20: 368.](http://paperpile.com/b/JoQtIv/sivAB)
13. [Trapero-Bertran M, Mistry H, Shen J, Fox-Rushby J. A systematic review and meta-analysis of willingness-to-pay values: the case of malaria](http://paperpile.com/b/JoQtIv/Ew7aN) [control interventions. Health Econ. 2013;22: 428–450.](http://paperpile.com/b/JoQtIv/Ew7aN)
14. [Njie H, Wangen KR, Chola L, Gopinathan U, Mdala I, Sundby JS, et al. Willingness to pay for a National Health Insurance Scheme in The](http://paperpile.com/b/JoQtIv/ikf0o) [Gambia: a contingent valuation study. Health Policy Plan. 2023;38: 61–73.](http://paperpile.com/b/JoQtIv/ikf0o)
15. [Unicef, Others. Long-lasting Insecticidal Nets: Supply Update UNICEF Supply Division. United Nations International Childrenʼs Emergency](http://paperpile.com/b/JoQtIv/taCH4) [Fund, New York, USA. 2020.](http://paperpile.com/b/JoQtIv/taCH4)
16. [Masum H, Shah R, Schroeder K, Daar AS, Singer PA. Africa’s largest long-lasting insecticide-treated net producer: lessons from A to Z](http://paperpile.com/b/JoQtIv/VTtvL) [Textiles. BMC Int Health Hum Rights. 2010;10 Suppl 1: S6.](http://paperpile.com/b/JoQtIv/VTtvL)
17. [World Health Organization. Guidance framework for testing of genetically modified mosquitoes, second edition. World Health Organization;](http://paperpile.com/b/JoQtIv/fhrvj) [2021 May. Report No.: Licence: CC BY-NC-SA 3.0 IGO. Available:](http://paperpile.com/b/JoQtIv/fhrvj) <https://www.who.int/publications/i/item/9789240025233>
18. [Service MW. Studies on sampling larval populations of the Anopheles gambiae complex. Bull World Health Organ. 1971;45: 169–180.](http://paperpile.com/b/JoQtIv/vTwZM)
19. [Killeen GF, McKenzie FE, Foy BD, Schieffelin C, Billingsley PF, Beier JC. A simplified model for predicting malaria entomologic](http://paperpile.com/b/JoQtIv/lSyJJ) [inoculation rates based on entomologic and parasitologic parameters relevant to control. Am J Trop Med Hyg. 2000;62: 535–544.](http://paperpile.com/b/JoQtIv/lSyJJ)
20. [Bayoh MN, Lindsay SW. Effect of temperature on the development of the aquatic stages of Anopheles gambiae sensu stricto (Diptera:](http://paperpile.com/b/JoQtIv/Whjyf) [Culicidae). Bull Entomol Res. 2003;93: 375–381.](http://paperpile.com/b/JoQtIv/Whjyf)
21. [Kirby MJ, Lindsay SW. Effect of temperature and inter-specific competition on the development and survival of Anopheles gambiae sensu](http://paperpile.com/b/JoQtIv/2l9vD) [stricto and An. arabiensis larvae. Acta Trop. 2009;109: 118–123.](http://paperpile.com/b/JoQtIv/2l9vD)
22. [Gething PW, Van Boeckel TP, Smith DL, Guerra CA, Patil AP, Snow RW, et al. Modelling the global constraints of temperature on](http://paperpile.com/b/JoQtIv/jaYn1) [transmission of Plasmodium falciparum and P. vivax. Parasit Vectors. 2011;4: 92.](http://paperpile.com/b/JoQtIv/jaYn1)
23. [Wu SL, Bennett JB, Sánchez C HM, Dolgert AJ, León TM, Marshall JM. MGDrivE 2: A simulation framework for gene drive systems](http://paperpile.com/b/JoQtIv/MiB4O) [incorporating seasonality and epidemiological dynamics. PLoS Comput Biol. 2021;17: e1009030.](http://paperpile.com/b/JoQtIv/MiB4O)
24. [Yé Y, Hoshen M, Kyobutungi C, Louis VR, Sauerborn R. Local scale prediction of Plasmodium falciparum malaria transmission in an](http://paperpile.com/b/JoQtIv/iRkOI) [endemic region using temperature and rainfall. Glob Health Action. 2009;2. doi:](http://paperpile.com/b/JoQtIv/iRkOI)[10.3402/gha.v2i0.1923](http://dx.doi.org/10.3402/gha.v2i0.1923)
25. [Molineaux L, Gramiccia G. The Garki Project. 1980.](http://paperpile.com/b/JoQtIv/6t8p)
26. [Soumare HM, Dabira ED, Camara MM, Jadama L, Gaye PM, Kanteh S, et al. Entomological impact of mass administration of ivermectin and](http://paperpile.com/b/JoQtIv/HZZ7c) [dihydroartemisinin-piperaquine in The Gambia: a cluster-randomized controlled trial. Parasit Vectors. 2022;15: 435.](http://paperpile.com/b/JoQtIv/HZZ7c)
27. [Pfeffer DA, Lucas TCD, May D, Harris J, Rozier J, Twohig KA, et al. malariaAtlas: an R interface to global malariometric data hosted by the](http://paperpile.com/b/JoQtIv/wcAyh)

[Malaria Atlas Project. Malar J. 2018;17: 352.](http://paperpile.com/b/JoQtIv/wcAyh)

1. [T.C.UNIONAGROTECH Co.,Ltd Tuna Meal, Fish Meal, ผลิตและจําหน่ายวัตถุดิบและอาหารสัตว์. [cited 13 Nov 2024]. Available:](http://paperpile.com/b/JoQtIv/zfZK) <https://www.tcunionagrotech.com/#contact>
